# Electrical acupoint stimulation for gastrointestinal recovery after gastrectomy for gastric cancer: a systematic review and meta-analysis

**DOI:** 10.3389/fsurg.2026.1915755

**Published:** 2026-09-16

**Authors:** Dong-Hyeon Oh, Yong-Eun Park, Hyun Dong Chae

**Affiliations:** 1 Department of Surgery, Yeungnam University College of Medicine, Daegu, Republic of Korea; 2 Department of Surgery, Daegu Catholic University School of Medicine, Daegu, Republic of Korea

**Keywords:** electrical acupoint stimulation, electroacupuncture, enhanced recovery after surgery, gastrectomy, gastric cancer, meta-analysis, gastrointestinal recovery, transcutaneous electrical acupoint stimulation

## Abstract

Delayed gastrointestinal recovery remains a challenge after gastrectomy for gastric cancer, with residual variability even within enhanced recovery after surgery (ERAS) pathways. This residual variability has prompted interest in adjunctive approaches such as electrical acupoint stimulation (EAS), but gastrectomy-specific anatomical and neural features may limit extrapolation from broader abdominal-surgery populations. We therefore synthesized randomized evidence on EAS for postoperative gastrointestinal recovery and examined variation across study-level characteristics. We searched PubMed, Embase, Scopus, CENTRAL, China National Knowledge Infrastructure, Wanfang Data, SinoMed, and four registries from inception to a 1 August 2026 cutoff. We included randomized trials in patients undergoing gastrectomy for gastric cancer when EAS was separable from other traditional or acupoint-based co-interventions. Outcomes were time to first flatus (primary), first defecation, first liquid intake, and length of hospital stay. Safety was summarized descriptively. Risk of bias was assessed with the Cochrane Risk of Bias 2 tool and certainty with GRADE; exploratory subgroup differences were examined using interaction tests. Fifteen trials were eligible; 13 contributed to the primary pairwise comparison (1,357 participants) and two to narrative synthesis only. EAS was associated with shorter times to first flatus [13 trials; mean difference (MD), −9.61 h; 95% CI, −12.81 to −6.42; *I*^2^ = 82.6%; very low certainty], first defecation (9 trials; MD, −15.88 h; 95% CI, −23.31 to −8.46; *I*^2^ = 90.3%; low certainty), and first liquid intake (2 trials; MD, −10.65 h; 95% CI, −18.20 to −3.10; *I*^2^ = 0%; low certainty). Length of hospital stay did not differ significantly (7 trials; MD, −0.63 days; 95% CI, −1.36 to 0.10; *I*^2^ = 57.0%; low certainty). Prediction intervals narrowly excluded the null for flatus but included it for defecation. For flatus, funnel-plot asymmetry and trim-and-fill attenuation raised publication-bias concerns. None of the 12 primary interaction tests across comparator design, perioperative care, stimulation modality, and frequency strategy was significant. Safety reporting was limited; no serious intervention-related adverse events were reported where assessed. Current evidence indicates a consistent directional signal favoring EAS for early postoperative gastrointestinal recovery milestones, but the magnitude, reproducibility, and clinical importance of this association remain uncertain.

**Systematic Review Registration:** https://www.crd.york.ac.uk/PROSPERO/view/CRD420261289414, identifier CRD420261289414.

## Introduction

Postoperative gastrointestinal recovery is a major component of early recovery after abdominal surgery. Delayed recovery of bowel function can impede oral intake, prolong length of hospital stay (LOS), increase healthcare use, and adversely affect patients' quality of life (1–4). In clinical studies, gastrointestinal recovery is commonly assessed using postoperative milestones such as time to first flatus, first defecation, and resumption of oral intake (5), while LOS provides a broader measure of postoperative recovery. Enhanced recovery after surgery (ERAS) protocols aim to accelerate recovery through multimodal perioperative strategies, including minimally invasive surgery, multimodal analgesia, early mobilization, and early postoperative feeding (6, 7). Nevertheless, gastrointestinal recovery remains variable even within ERAS pathways (8). This residual variability has prompted interest in adjunctive interventions that may further facilitate gastrointestinal recovery alongside established perioperative care.

Electrical acupoint stimulation (EAS), encompassing electroacupuncture (EA) and transcutaneous electrical acupoint stimulation (TEAS), is one such adjunctive approach. Its electrical stimulation parameters, including frequency and intensity, can be explicitly specified, facilitating characterization and reproducibility of treatment protocols in clinical research (9, 10). A meta-analysis of abdominal surgery reported earlier postoperative gastrointestinal recovery with electroacupuncture or transcutaneous electroacupuncture (11). More recently, a meta-analysis evaluated TEAS as an adjunctive therapy for ERAS protocols in abdominal surgery and reported earlier gastrointestinal recovery and shorter LOS (12).

Whether these findings can be extrapolated to patients undergoing gastrectomy for gastric cancer remains uncertain. Gastrectomy alters upper gastrointestinal anatomy through gastric resection and reconstruction, while postoperative function may also be influenced by the extent to which perigastric vagal innervation is disrupted or preserved (13, 14). The clinical relevance of this neural context is illustrated by a recent randomized trial in patients undergoing distal gastrectomy for early gastric cancer, in which vagus-preserving surgery was associated with a lower incidence of postsurgical gastroparesis than was observed with vagus-resecting surgery (15). These procedure-specific anatomical and physiological features provide a rationale for evaluating postoperative gastrointestinal recovery interventions specifically after gastrectomy rather than directly extrapolating findings from broader abdominal surgical populations.

Previous reviews in gastric cancer populations have evaluated acupuncture- and moxibustion-related interventions broadly, including their use for postoperative gastrointestinal recovery after surgery (16, 17). However, we did not identify a previous meta-analysis focused specifically on EAS, distinct from broader traditional or acupoint-based interventions, in patients undergoing gastrectomy for gastric cancer. Within the EAS evidence base itself, trials also differ in comparator design, perioperative care protocol, stimulation modality, and stimulation-frequency strategy. These differences raise questions about whether pooled estimates vary across these study-level characteristics.

Therefore, we conducted a systematic review and meta-analysis of randomized controlled trials (RCTs) evaluating EAS for postoperative gastrointestinal recovery in patients undergoing gastrectomy for gastric cancer. We restricted eligibility to studies in which EAS could be evaluated separately from other traditional or acupoint-based co-interventions. We synthesized time to first flatus, time to first defecation, time to first liquid intake, and LOS, and summarized safety outcomes. We also conducted exploratory subgroup analyses according to comparator design, perioperative care protocol, stimulation modality, and stimulation-frequency strategy. We assessed the certainty of the evidence and examined the sensitivity of the findings to key analytical and study-selection decisions.

## Materials and methods

### Protocol and registration

This systematic review and meta-analysis was reported in accordance with the Preferred Reporting Items for Systematic Reviews and Meta-Analyses (PRISMA) 2020 statement (18). The protocol was registered with the International Prospective Register of Systematic Reviews (PROSPERO; CRD420261289414; registered 21 January 2026, review commenced 26 January 2026). The PROSPERO record was amended in August 2026. The amendment broadened the population criterion from adults undergoing radical gastrectomy with lymphadenectomy to adults undergoing gastrectomy for gastric cancer, added an exclusion for trials enrolling patients only after an established postoperative gastrointestinal motility disorder had developed, and clarified the exclusion of separately prescribed traditional or acupoint-based co-interventions. The eligibility criteria applied in this review are detailed below.

### Data sources and search strategy

We searched seven bibliographic databases—PubMed, CENTRAL (via the Cochrane Library), Embase, Scopus, the China National Knowledge Infrastructure (CNKI), Wanfang Data, and SinoMed—and four trial registries: ClinicalTrials.gov, the World Health Organization International Clinical Trials Registry Platform (ICTRP), the Chinese Clinical Trial Registry (ChiCTR), and the International Traditional Medicine Clinical Trial Registry (ITMCTR). All sources were searched from inception, where applicable, without language restrictions, with a final search cutoff of 1 August 2026. Searches of PubMed, CENTRAL, Embase, Scopus, CNKI, Wanfang Data, and the four trial registries were initially conducted on 16 March 2026 and updated on 1 August 2026. Because the review team could not obtain access to SinoMed sufficient for a reproducible search, the database provider ran the strategy specified by the review team on 12 August 2026 and supplied the complete line-by-line search history and exported records. Database update dates in the exported records were checked, and none was later than 1 August 2026.

Search strategies were adapted to each source. In PubMed, CENTRAL, Embase, and Scopus, a gastric cancer or gastrectomy concept was combined with an acupuncture or electrical acupoint stimulation concept, using controlled vocabulary together with free-text terms where supported by the database. PubMed, Embase, and Scopus additionally included a trial-design concept, whereas CENTRAL did not. In the Chinese-language databases, condition and intervention terms were searched in the title, keyword, and abstract fields in CNKI, in the Topic field in Wanfang Data, and in the general fields in SinoMed, with a supplementary search of controlled subject headings in SinoMed. No study-design filter was applied in any of the three Chinese-language databases.

Trial registries were searched using source-specific keyword and field-based strategies, without restrictions on recruitment status and without randomized-trial terms. We also screened the reference lists of all included trials and of two topic-specific Chinese-language systematic reviews. Complete source-specific search strategies, including interface settings and record counts, are provided in Supplementary Table S1.

### Eligibility criteria

Eligibility was defined according to the PICOS framework.

1. **Participants (P)**: Eligible studies enrolled adults (aged ≥18 years) with gastric cancer undergoing gastrectomy. Eligibility did not depend on cancer stage, explicit characterization of the operation as radical or curative-intent, resection-margin status, or the performance or extent of lymph-node dissection. Any extent of gastric resection and open, laparoscopic, or robotic approaches were permitted; operative characteristics were treated as descriptive study features rather than eligibility requirements. Eligible trials evaluated EAS in the context of routine perioperative or early postoperative gastrointestinal recovery. Studies were excluded if they enrolled patients only after an established postoperative gastrointestinal motility disorder—such as postoperative ileus, intestinal paralysis, gastroparesis, or delayed gastric emptying—had developed, as these addressed treatment of an established complication rather than facilitation of routine recovery. Mixed populations were eligible only when data for patients with gastric cancer undergoing gastrectomy were separately extractable.
2. **Interventions (I)**: Eligible interventions were electroacupuncture (EA) or transcutaneous electrical acupoint stimulation (TEAS), collectively classified as electrical acupoint stimulation (EAS). EA was defined as acupuncture in which electrical stimulation was delivered through inserted needles at predefined acupoints, and TEAS as transcutaneous electrical stimulation delivered through surface electrodes positioned over predefined acupoints. The intervention had to be described sufficiently to classify it as EA or TEAS and to characterize the stimulation protocol. Protocols had to include at least one postoperative stimulation session, so that stimulation delivered exclusively before or during surgery was not eligible. Trials in which EAS was accompanied by another traditional or acupoint-based treatment prescribed separately from the EAS protocol—such as herbal medicine, moxibustion, manual acupuncture, or acupressure—were excluded even when that treatment was provided to both randomized groups, to maintain applicability to EAS added to conventional or ERAS-based perioperative care. In applying this criterion, non-electrified needles used within the same prescribed EA session as part of the acupuncture protocol were considered part of the EA intervention, whereas needling prescribed separately from the EA protocol was not considered part of the EA intervention. Conventional perioperative co-interventions, including usual care, ERAS pathways, analgesia, nutritional support, and mobilization, were permitted.
3. **Comparisons (C)**: Eligible comparators included sham or placebo-like acupuncture or stimulation, conventional or usual perioperative care without active EAS, or the same ERAS-based perioperative pathway without active EAS.
4. **Outcomes (O)**: The primary outcome was time to first flatus. Secondary outcomes were time to first defecation, time to first liquid intake, and length of hospital stay. Studies reporting at least one of these outcomes with sufficient information for quantitative or narrative synthesis were eligible. Time to first liquid intake was defined as the time from surgery to first postoperative water intake or initiation of sips of water; initiation or tolerance of a liquid or soft diet was not considered equivalent.
5. **Study design (S)**: Randomized controlled trials with results reported in full-text journal articles were eligible, without language restriction. Non-randomized studies and ineligible publication types, including case reports, reviews, animal studies, errata, letters, conference abstracts, theses or dissertations, published protocols, and trial-registration records without results, were excluded. Ongoing or awaiting-results studies were recorded separately and were not included in the evidence synthesis.

### Study selection process

Following initial deduplication, two reviewers (YEP and DHO) independently screened all records identified through database, registry, and reference-list searching. Full-text reports or complete registry records of potentially eligible studies were then assessed independently for final eligibility. Non-English full-text reports were translated as needed before eligibility assessment. Disagreements at either stage were resolved through discussion or, when necessary, consultation with a third reviewer (HDC).

Seven additional records were recognized as duplicates only after screening had begun. For PRISMA counting, these records were retrospectively classified as duplicates and removed from the final count of unique records screened. Multiple reports describing the same underlying randomized trial were linked and treated as a single study. For each such study, one report was designated as the report of record, and all extracted data were taken from that report. Additional linked reports were not counted as separate studies and were used only for contextual information where explicitly indicated.

In multi-arm trials, the eligibility of individual intervention and comparator arms was assessed separately. Arms that did not meet the eligibility criteria did not contribute to the analyses; in Gu et al. 2018 (19), stimulation in the short-duration arm was delivered exclusively during surgery, so only the long-duration stimulation and control arms contributed. For trials contributing to the pairwise meta-analysis, when both a sham-control comparison and another eligible comparison were available, the sham-control comparison was selected for the primary analysis, and the alternative eligible comparison was examined in a sensitivity analysis. Accordingly, in Pei et al. (20), the electroacupuncture-vs.-sham-electroacupuncture comparison was used in the primary analysis, whereas the standard-care comparison was evaluated in a sensitivity analysis (see Statistical analysis).

When reports appeared potentially related because of shared or closely related trial-registration or ethics identifiers, institutions, investigators, or recruitment periods, we compared registration and ethics information, centers and investigators, recruitment periods and participant flow, and intervention and outcome profiles, and contacted investigators when necessary to clarify whether the randomized samples were independent (Supplementary Table S6). When the independence of a reported randomized sample from another report could not be adequately established, that report was not treated as independent evidence and was excluded from the review. For potentially related reports retained as independent studies, residual uncertainty was additionally examined in a sensitivity analysis (see Statistical analysis).

One review author (HDC) is a co-author of one included trial, Jung et al. (21). To manage this potential competing interest, HDC did not participate in the eligibility assessment, data extraction, outcome-specific risk-of-bias assessment, or adjudication for that trial; these activities were completed by YEP and DHO.

### Data collection process

For each included study, data extraction was based on the designated report of record. Two reviewers (YEP and DHO) independently extracted study characteristics (first author, publication year, and country); participant and operative characteristics (randomized and analyzed sample sizes, age, sex, cancer stage, surgical approach, gastrectomy type, reconstruction type, and extent of lymphadenectomy, when reported); perioperative care; intervention and comparator characteristics (stimulation modality, comparator design, acupoints, stimulation frequency, waveform and intensity, timing of treatment initiation, session duration, number of sessions, and practitioner background); postoperative gastrointestinal recovery outcomes (time to first flatus, time to first defecation, time to first liquid intake, and LOS); and safety outcomes, including intervention-related adverse events and postoperative complications. Reported outcome measures were mapped to the review outcomes according to the definitions specified above. When a report described a first feeding or oral intake milestone without establishing that it represented first postoperative water intake or initiation of sips of water, that measure was not mapped to time to first liquid intake. This applied to Cai et al. (22), Ding and Huang (23), and Huang and Li (24). For LOS, when a report distinguished postoperative from total hospital stay, the postoperative measure was used for synthesis; otherwise, the hospital-stay measure reported by the trial was retained. Any discrepancies in extracted data were resolved through discussion between YEP and DHO or, when necessary, consultation with HDC, except for Jung et al. (21), for which HDC was recused as described above.

In a *post hoc* clinical-outcome assessment, each included trial was additionally reviewed for an explicit trial-defined definition or threshold for prolonged postoperative ileus (POI) and for related indicators of postoperative gastrointestinal dysfunction, including nasogastric-tube reinsertion, reinstitution of fasting, initiation or reinstitution of parenteral nutrition, and other rescue interventions when explicitly reported as management for ileus, inability to tolerate oral intake, postoperative nausea or vomiting, abdominal distension, and composite clinical definitions of POI or gastrointestinal recovery. These additional outcomes were considered for quantitative synthesis only when their definitions and reporting formats were sufficiently comparable; otherwise, they were summarized narratively.

### Risk of bias assessment

Risk of bias was independently assessed by two reviewers (YEP and DHO) using the Cochrane Risk of Bias 2 (RoB 2) tool (25), with the effect of assignment to intervention as the effect of interest. Assessments were conducted separately for each study–outcome result for time to first flatus, time to first defecation, time to first liquid intake, and LOS. Outcome-specific results from the two trials included only in the narrative synthesis were assessed using the same framework; the synthesis to which each result contributed and the corresponding domain-level and overall judgments are summarized in Supplementary Table S3. For the four-arm trial by He et al. (26), two comparisons were assessed for each relevant outcome: stimulation added to a fast-track pathway vs. that pathway alone, and stimulation added to conventional care vs. conventional care alone.

For each study–outcome result, five domains were evaluated: (1) bias arising from the randomization process, (2) bias due to deviations from intended interventions, (3) bias due to missing outcome data, (4) bias in measurement of the outcome, and (5) bias in selection of the reported result. Overall risk-of-bias judgments were derived from the domain-level judgments following the RoB 2 algorithm; where more than one domain raised some concerns, the combined implications for that result were considered rather than applying a fixed rule. Domain 2 was judged on the basis of documented deviations from the intended interventions arising because of the trial context, imbalances in co-interventions, and the analysis used to estimate the effect of assignment. Absence of blinding alone was not considered sufficient to raise concerns in this domain; the potential influence of knowledge of treatment assignment on outcome ascertainment was assessed separately in Domain 4. Full signaling-question responses, supporting evidence, and judgment rationales are provided in Supplementary Table S4. Disagreements between YEP and DHO were resolved through discussion or, when necessary, consultation with HDC, subject to the recusal described above.

### Study-level categorization for subgroup analyses

The final subgroup-analysis framework was not fully prespecified in PROSPERO and was treated as exploratory. It examined four study-level characteristics: comparator design, perioperative care protocol, stimulation modality, and stimulation-frequency strategy.

#### Comparator design

Trials were classified as sham-controlled or no-sham. Sham-controlled trials used a sham or placebo-like acupuncture or stimulation comparator, whereas no-sham trials compared EAS with conventional or usual perioperative care or with the same ERAS-based pathway without active EAS. This classification reflected comparator design and was not interpreted as evidence of successful masking.

#### Perioperative care protocol

Perioperative care was classified as ERAS-based or conventional care from the practices described as actually implemented in both randomized groups, rather than from the authors' use of ERAS, fast-track, or rapid-recovery terminology. Reported practices were mapped to eight review-defined domains: preoperative optimization or prehabilitation, abbreviated fasting or carbohydrate loading, intraoperative stress reduction, multimodal or opioid-sparing analgesia, early oral or enteral intake, early mobilization, avoidance or early removal of nasogastric, drainage, or urinary tubes, and protocolized functional discharge criteria; the last five were treated as postoperative-recovery domains. A trial was classified as ERAS-based when at least three distinct qualifying domains were described as implemented, including at least one postoperative-recovery domain, and as conventional care otherwise. This threshold was an operational rule for the present exploratory analysis rather than an ERAS Society definition. Trial-level domain assessments and the resulting classifications are provided in Supplementary Table S7.

#### Stimulation modality

Trials were classified as EA or TEAS according to the intervention definitions specified in the eligibility criteria.

#### Stimulation-frequency strategy

Stimulation protocols were classified according to whether the stimulation frequency varied over time during a treatment session. Fixed-frequency protocols used a frequency that remained constant over time at each stimulated site, including protocols in which different fixed frequencies were assigned to different acupoints. Alternating-frequency protocols switched between two frequencies over time during stimulation, such as 2/100 Hz or 20/100 Hz.

### Statistical analysis

All statistical analyses were performed in R (version 4.6.1) using the metafor package (version 5.0-1) (27), and forest plots were generated using the forestploter package (version 1.1.4). Pooled effects for continuous outcomes were expressed as mean differences (MDs) with 95% confidence intervals (CIs). MDs were calculated as EAS minus comparator, so that negative values favored EAS, corresponding to earlier gastrointestinal recovery or shorter LOS. Gastrointestinal recovery-time outcomes reported in days by Jung et al. (21), Ding and Huang (23), and Huang and Li (24) were converted to hours using 24 h per day, whereas LOS was retained in days. For Cai et al. (22), means and standard deviations for time to first flatus and LOS were estimated from the reported medians and interquartile ranges using the method of Wan et al. (28); the original summary statistics and derived values are provided in Supplementary Table S5.

Time to first flatus, time to first defecation, and time to first liquid intake were synthesized as continuous elapsed-time outcomes because arm-level means and standard deviations, or summary statistics convertible to them, were the only data reported consistently enough to support the same effect measure across trials. Pei et al. (20) additionally reported an extractable hazard ratio for time to first flatus, the only such estimate among the included trials. Because hazard ratios and mean differences represent different estimands, hazard ratios were not pooled; the estimate from Pei et al. was reported separately.

Because elapsed-time outcomes are bounded below by zero and may be right-skewed, we conducted a *post hoc* distributional plausibility assessment of the summary statistics used in the quantitative synthesis. For each outcome, and for every study arm in the primary pairwise comparisons with an available mean and standard deviation, whether reported directly or derived from a median and interquartile range, we calculated the percentage of a normal distribution with that mean and standard deviation that would fall below zero, an impossible value for an elapsed-time outcome (the fitted-normal probability below zero). This diagnostic was used only to identify gross incompatibility between the summary statistics used in the meta-analysis and a normal approximation; it did not establish that the underlying distributions were normal or exclude right-skewness. For values derived from medians and interquartile ranges, the diagnostic also did not independently verify the conversion. For Cai et al. (22), we additionally assessed within-IQR asymmetry by calculating the relative position of the median within the interquartile range as (median−Q1)/(Q3−Q1), with 0.50 indicating equal distances from Q1 and Q3. Because the reported quartiles were asymmetric for some outcome–arm combinations, the converted means and standard deviations were treated as approximations, and their influence was examined by excluding the trial in a sensitivity analysis.

A random-effects model was used as the primary analytical approach because clinical and methodological heterogeneity was anticipated; model choice was not based on the result of a heterogeneity test. Between-study variance (*τ*^2^) was estimated using restricted maximum likelihood (REML). When *τ*^2^ was greater than zero and more than two trials contributed to an analysis, confidence intervals and tests of pooled effects used the Hartung–Knapp–Sidik–Jonkman (HKSJ) method (29), based on the t distribution with k−1 degrees of freedom, where k denotes the number of contributing trials. Wald-type inference was used otherwise.

For each primary meta-analysis, statistical heterogeneity was summarized using *τ*^2^ with its 95% Q-profile confidence interval, Cochran's Q with its degrees of freedom and *P* value, and *I*^2^. For analyses with at least five contributing trials, 95% prediction intervals were calculated as θ^^^ ± t_k−1, 0.975_ × √(τ^^^^2^ + SE^2^), where k denotes the number of contributing trials, θ^^^ is the pooled estimate, and SE is its Hartung–Knapp-adjusted standard error. Prediction intervals were not calculated when fewer than five trials contributed.

Exploratory subgroup analyses were conducted for time to first flatus, time to first defecation, and LOS according to comparator design, perioperative care protocol, stimulation modality, and stimulation-frequency strategy. Within each characteristic, *τ*^2^ was estimated separately for each subgroup, and the conditional rule for HKSJ vs. Wald-type inference described above was applied to the subgroup-specific pooled effects. Differences between subgroups were assessed primarily using Knapp–Hartung *F* tests from random-effects meta-regression models assuming a common *τ*^2^ across the subgroups being compared (30); conventional Q tests for subgroup differences based on subgroup-specific *τ*^2^ estimates were reported as a secondary comparison. Because relatively few trials contributed to each outcome and to individual subgroups, estimation of subgroup-specific heterogeneity and evaluation of the common-*τ*^2^ assumption underlying the meta-regression were limited; both interaction approaches were therefore regarded as exploratory. Effect modification was evaluated from the interaction tests; subgroup-specific *τ*^2^ and *I*^2^ values and the statistical significance of individual subgroup estimates were treated as descriptive and were not considered evidence of effect modification. Subgroup analyses were not performed for time to first liquid intake because only two trials contributed; both used EA with fixed-frequency stimulation, while comparator design and perioperative care protocol each comprised one trial per subgroup.

Sensitivity to analytical choices was examined by varying the between-study variance estimator and confidence-interval method and by fitting a common-effect model. Alternative random-effects specifications included a modified HKSJ interval incorporating the *ad hoc* variance correction (29), with the full set of model specifications reported in Table 4, Panel A. To assess the influence of individual trials, leave-one-out analyses were performed for outcomes with at least three contributing trials, with complete results provided in Supplementary Table S9.

Additional *post hoc* sensitivity analyses examined robustness to study inclusion, outcome-specific risk of bias, data interpretation, and comparator choice. For each applicable outcome, pooled estimates were recalculated after excluding study–outcome results judged to be at high overall risk of bias, trials without a sham comparator, and Cai et al. (22), for which means and standard deviations were derived from medians and interquartile ranges. Additional analyses retained only Pei et al. (20) among the three investigator- and institution-related trials, excluded the largest trial, and excluded Gu et al. 2018 (19), for which the reported time unit required interpretation. In a separate comparator-choice analysis, the standard-care arm of Pei et al. was substituted for its sham arm. No pooled estimate was calculated when only one trial remained. These analyses are reported in Table 4, Panel B.

Small-study effects and funnel-plot asymmetry were assessed for outcomes with at least 10 contributing trials using a contour-enhanced funnel plot (Supplementary Figure S3A), the classical Egger regression test with the standard error as the predictor (weighted linear regression with multiplicative dispersion) (31), and the Begg–Mazumdar rank-correlation test (32). Among the review outcomes, only time to first flatus met this threshold. The trim-and-fill procedure was applied as a sensitivity analysis using the L0 estimator, with the side determined automatically by the function (33); the resulting augmented dataset was refitted using REML with the same conditional HKSJ/Wald-type inference rule as in the primary analyses.

Safety outcomes were summarized descriptively rather than quantitatively pooled because outcome definitions, ascertainment, and reporting formats varied across trials. Participant-level event counts and corresponding denominators were tabulated where they could be reliably reconstructed.

### Certainty of evidence assessment

The certainty of evidence for each quantitative synthesis of time to first flatus, time to first defecation, time to first liquid intake, and LOS was assessed using the Grading of Recommendations Assessment, Development and Evaluation (GRADE) approach (34). Risk-of-bias judgments for each outcome were informed by the corresponding outcome-specific RoB 2 assessments of the results contributing to that synthesis. Two reviewers (YEP and DHO) independently evaluated certainty across five domains: risk of bias, inconsistency, indirectness, imprecision, and publication bias. Disagreements were resolved through discussion; no third-reviewer adjudication was required.

Because all contributing studies were randomized trials, certainty started at high and was downgraded by one or two levels when serious or very serious concerns, respectively, were identified in any of the five domains. Publication bias was judged from the totality of the available evidence, including formal small-study-effect assessments where applicable, rather than from any single statistical test.

Imprecision was assessed using both the 95% confidence interval in relation to the applicable threshold of clinical importance and the optimal information size (OIS). If the confidence interval included effects on both sides of the threshold, imprecision was judged serious regardless of the OIS. When it did not, the OIS was also required to be met to avoid downgrading. The OIS criterion was evaluated using the median control-group standard deviation, while the value based on the largest observed control-group standard deviation was reported as an upper-bound estimate within the observed SD range. OIS was calculated as the total sample size required for a two-group comparison with equal group sizes, a two-sided *α* of 0.05, and 80% power, using the median and the largest observed control-group standard deviations among the trials contributing to that outcome. The required sample size per group was rounded up to the next whole participant before doubling to obtain the total OIS. Because no validated minimal important difference was identified for the gastrointestinal recovery milestones, a difference of 8 h was applied *post hoc* as a pragmatic threshold to each of the three recovery milestones, chosen to represent a within-day shift capable of altering the timing of subsequent postoperative management, and a difference of 1 day was used for LOS, reflecting the avoidance of an additional hospital night. Thresholds of 6 and 12 h were examined in sensitivity assessments for the recovery milestones.

## Results

### Study selection

The database and registry searches identified 1,176 records, comprising 959 records from bibliographic databases and 217 from trial registries. After removal of 432 duplicate records, 744 unique records were screened by title and abstract. Of these, 604 were excluded at this stage and two were identified as ongoing or awaiting results. Reports were sought for the remaining 138 records; all were retrieved and assessed for eligibility. At this stage, 115 reports were excluded (Supplementary Table S8) and a further five were identified as ongoing or awaiting results, leaving 18 reports representing 15 unique randomized trials. Three of the 18 reports were linked reports of trials already represented by another included report and were therefore not counted as separate studies (Supplementary Table S6). Seven records corresponding to ongoing or awaiting-results studies were documented separately in the PRISMA flow diagram and were not counted among the exclusion reasons. Reference-list searching of all 15 included trials and two topic-specific Chinese-language systematic reviews identified four additional records; all four were screened and none met the eligibility criteria. Thirteen trials contributed to the quantitative synthesis, and two were included in the narrative synthesis only. The study selection process is presented in Figure 1.

**Figure 1:**
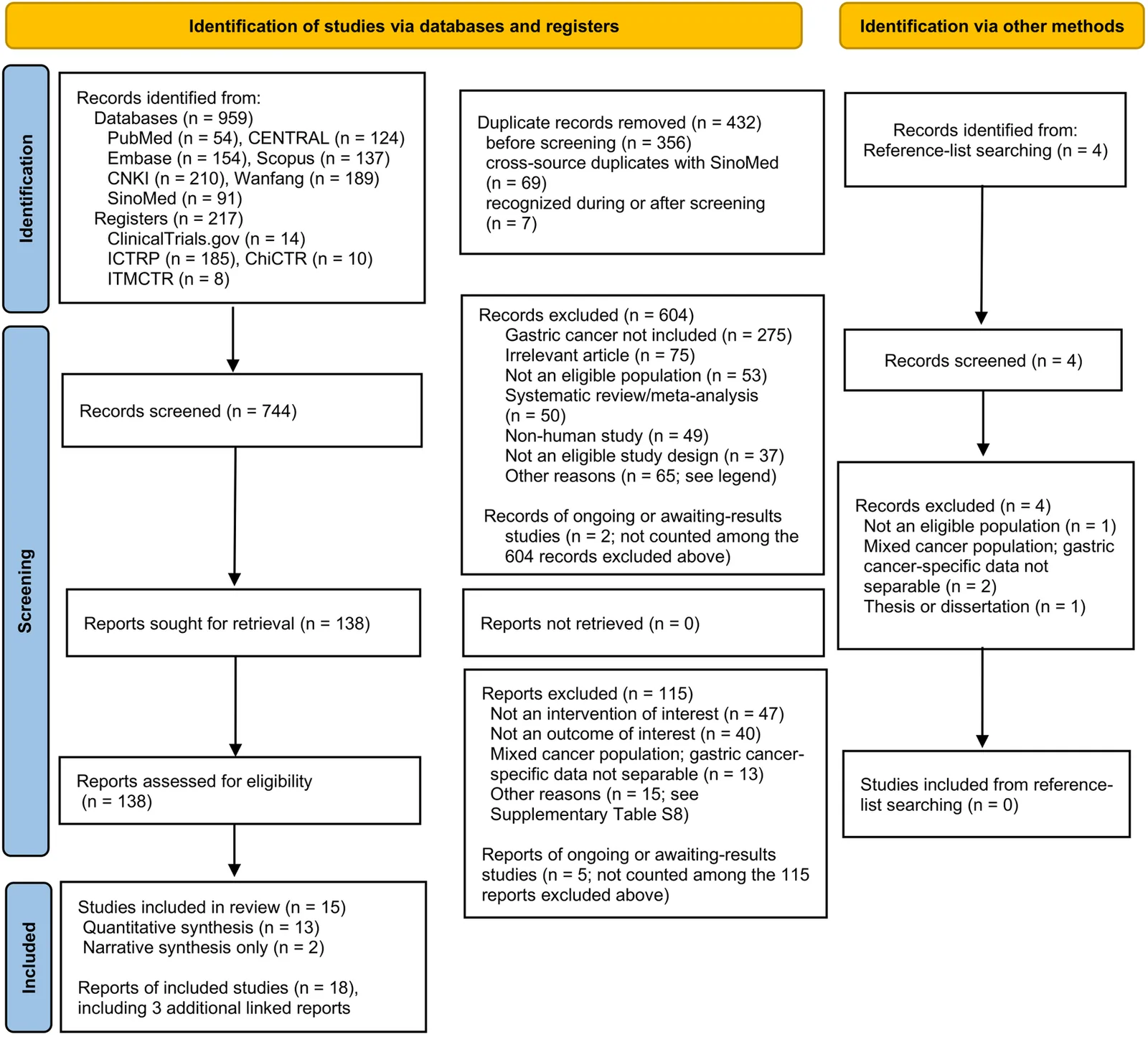
PRISMA 2020 flow diagram of study identification, screening, and inclusion. Database and registry sources were searched from inception, where applicable, through 1 August 2026. The SinoMed search was executed on 12 August 2026 while the review cutoff remained 1 August 2026; the database update dates recorded in the exported records were checked, and none was later than 1 August 2026. Full search strategies are provided in Supplementary Table S1. Other reasons for exclusion at title-and-abstract screening were erratum, letter, conference abstract, or case report (*n* = 23); not an intervention of interest (*n* = 12); not an outcome of interest (*n* = 9); intervention administered exclusively before or during surgery (*n* = 8); published protocol or trial-registration record only (*n* = 8); intervention not separable from a co-intervention (*n* = 4); and mixed cancer population with gastric cancer-specific data not separable (*n* = 1). Reasons for reports excluded at full-text assessment are provided in Supplementary Table S8, and relationships among reports of the same study are provided in Supplementary Table S6. CENTRAL, Cochrane Central Register of Controlled Trials; ChiCTR, Chinese Clinical Trial Registry; CNKI, China National Knowledge Infrastructure; ICTRP, WHO International Clinical Trials Registry Platform; ITMCTR, International Traditional Medicine Clinical Trial Registry; SinoMed, Chinese Biomedical Literature Service System.

### Study characteristics

The baseline and surgical characteristics of the 13 trials contributing to the quantitative synthesis (19–24, 35–41) are summarized in Table 1. These RCTs were published between 2007 and 2026; 12 were conducted in China and one in Korea. They contributed 1,357 analyzed participants to the primary pairwise comparison, with analyzed comparison samples ranging from 36 to 383 participants per trial. Reported arm-level mean ages ranged from 50.0 to 72.4 years, and most participants were male. Laparoscopic gastrectomy was the predominant reported surgical approach. Two trials primarily involved open surgery, one trial included both laparoscopic and robotic gastrectomy, and five did not report the surgical approach. Guan et al. (40) included a single analyzed participant with stage IV disease. Cancer stage could not be harmonized to the I/II/III/IV categories in three trials and was not reported in three. Surgical approach and gastrectomy type were each not reported in five trials, reconstruction was not reported in six, and none of the 13 trials reported the extent of lymphadenectomy.

**Table 1:** Baseline and surgical characteristics of the trials contributing to the quantitative synthesis.

Study	Country	Randomized *n* (I/C)	Analyzed *n* (I/C)^a^	Sex (M/F)	Age, years (mean ± SD)	BMI, kg/m^2^ (mean ± SD)	Cancer stage (I/II/III/IV)	Surgical approach (open/lap/robotic)	Type of gastrectomy (TG/PG/DG)	Reconstruction (B-I/B-II/R-Y/other)
Zheng et al. (35)	China	20/20	20/20	I: 12/8 C: 11/9	I: 50 ± 4 C: 50 ± 4	NR	NR	NR	NR	NR
Chen et al. (36)	China	33/30	33/30	I: 22/11 C: 24/6	I: 63.0 ± 9.7 C: 59.0 ± 8.3	I: 22.3 ± 3.2 C: 21.9 ± 3.2	NR^d^	I: 23/10/0^c^ C: 23/7/0	NR	I: 10/11/12/0 C: 11/13/6/0
Gu et al. 2018 (19)	China	60/60^e^	60/60	I: 33/27 C: 28/32	I: 60.2 ± 7.6 C: 58.8 ± 8.7	I: 22.5 ± 2.3 C: 22.9 ± 2.5	NR^d^	I: 0/60/0 C: 0/60/0	I: 24/0/36 C: 21/0/39	I: 11/15/34/0 C: 12/10/35/0^f^
Gu et al. 2019 (37)	China	60/60	58/59	I: 31/27 C: 29/30	I: 57.6 ± 7.3 C: 56.7 ± 6.2	I: 22.7 ± 2.5 C: 21.8 ± 2.8	NR^d^	I: 0/58/0 C: 0/59/0	I: 22/18/18 C: 23/16/20	I: 16/20/22/0 C: 15/21/23/0
Zhou et al. (38)	China	41/41	41/40	I: 27/14 C: 28/12	I: 58.7 ± 9.3 C: 60.8 ± 9.1	I: 23.9 ± 2.8 C: 23.9 ± 2.7	I: 18/7/16/0 C: 16/8/16/0	I: 0/27/14 C: 0/23/17	I: 11/0/30 C: 5/0/35	I: 0/5/36/0 C: 0/10/30/0
Pei et al. (20)	China	195/190^b^	194/189	I: 128/66 C: 134/55	I: 59.7 ± 10.6 C: 60.4 ± 10.1	I: 22.8 ± 3.6 C: 23.2 ± 3.4	I: 65/59/70/0 C: 81/54/54/0	I: 0/194/0 C: 0/189/0	I: 55/10/129 C: 66/14/109	NR
Guan et al. (40)	China	22/22	19/18	I: 11/8 C: 12/6	I: 61.3 ± 12.1 C: 60.2 ± 11.0	I: 23.0 ± 2.9 C: 23.0 ± 3.5	I: 4/9/5/1 C: 9/3/6/0	NR	NR	I: 2/11/6/0 C: 3/4/11/0
Jung et al. (21)	Korea	20/20	18/18	I: 16/2 C: 16/2	I: 60.9 ± 9.4 C: 60.1 ± 13.2	I: 24.8 ± 3.9 C: 22.4 ± 3.6	I: 10/4/4/0 C: 7/6/5/0	I: 15/3/0 C: 16/2/0	I: 0/0/18 C: 0/0/18	I: 18/0/0/0 C: 18/0/0/0
Cai et al. (22)	China	51/50	50/48	I: 32/18 C: 28/20	I: 64.0 ± 9.8 C: 64.2 ± 11.0	I: 23.7 ± 3.3 C: 23.9 ± 2.8	I: 16/17/17/0 C: 13/14/21/0	I: 0/50/0 C: 0/48/0	I: 19/8/23 C: 16/8/24	I: 0/23/19/8^g^ C: 0/24/16/8
Ding and Huang (23)	China	30/30	30/30	I: 18/12 C: 17/13	I: 54.2 ± 8.0 C: 55.6 ± 10.6	I: 22.6 ± 3.4 C: 23.6 ± 5.7	NR	NR	I: 0/0/30 C: 0/0/30	NR
Wang et al. (41)	China	45/45	45/45	I: 35/10 C: 31/14	I: 60 ± 6 C: 59 ± 6	I: 22.2 ± 2.7 C: 22.5 ± 2.6	I: 15/22/8/0 C: 16/25/4/0	I: 0/45/0 C: 0/45/0	I: 0/11/34 C: 0/14/31	NR
Huang and Li (24)	China	26/26	26/26	I: 17/9 C: 15/11	I: 54.32 ± 5.43 C: 54.43 ± 5.54	NR	NR	NR	NR	NR
Shi et al. (39)	China	90/90	90/90	I: 50/40 C: 52/38	I: 72.40 ± 7.16 C: 71.28 ± 7.58	NR	I: 52/38/0/0 C: 54/36/0/0	NR	NR	NR

B-I, Billroth I; B-II, Billroth II; BMI, body mass index; C, control group; DG, distal gastrectomy; F, female; I, intervention group; lap, laparoscopic; M, male; NR, not reported; PG, proximal gastrectomy; R-Y, Roux-en-Y; SD, standard deviation; TG, total gastrectomy.

Participant and surgical characteristics are reported for the analyzed population of each trial and, where a trial was described in more than one publication, are taken from the designated report of record (Supplementary Table S6). Counts within each categorical variable sum to the analyzed group size unless otherwise stated. NR denotes data that were not reported or could not be harmonized to the categories used here.

a Analyzed numbers are those contributing to time to first flatus; group sizes for each outcome are shown in Figure 2.

b Pei et al. randomized 585 participants across three arms; five post-randomization exclusions could not be assigned to their original groups, so arm-specific randomized denominators could not be reconstructed and the displayed group sizes are those reported after these exclusions. Only the electroacupuncture and sham groups used in the primary analysis are shown.

c The open and laparoscopic counts for Chen et al. follow the Results text of that report; its baseline table presents them in reverse order.

d Stage could not be harmonized for three trials: Chen et al. reported pT and pN separately, whereas Gu et al. 2018 and Gu et al. 2019 used grouped stage categories that did not map directly to the I/II/III/IV categories used here.

e Gu et al. 2018 randomized 180 participants in equal numbers to three groups; only the long-duration stimulation and control groups are shown.

f The reconstruction counts for the control group of Gu et al. 2018 sum to 57 rather than 60 and are shown as published.

g Reconstruction counts for Cai et al. were derived from the reported procedure distribution using that trial's stated mapping of gastrectomy types to reconstruction methods; Giraffe reconstruction was classified as “other.”

The key intervention characteristics of these 13 trials are summarized in Table 2. Detailed information on stimulation parameters, needling/delivery methods, and treatment schedules is provided in Supplementary Table S2. By comparator design, seven trials were sham-controlled and six were no-sham. By perioperative care protocol, six trials were classified as ERAS-based and seven as conventional care. By stimulation modality, five trials used EA and eight used TEAS. By stimulation-frequency strategy, eight trials used alternating-frequency stimulation and five used fixed-frequency protocols. Across the stimulation protocols, Zusanli (ST36) was used in all 13 trials, followed by Neiguan (PC6) in 10.

**Table 2:** Key intervention characteristics and perioperative care protocols of the trials contributing to the quantitative synthesis.

Study	Modality	Control design	Acupoints used	Frequency setting	ERAS status
Zheng et al. (35)	TEAS	No-sham (the control group received no stimulation)	Unilateral LI4–PC8 electrode pair; bilateral ST36	Alternating 2/100 Hz	No
Chen et al. (36)	TEAS^a^	No-sham	PC6, ST36	Acupoint-specific fixed frequencies (ST36 25 Hz; PC6 100 Hz)^b^	No
Gu et al. 2018 (19)	TEAS	Sham (electrodes applied at the same acupoints and connected to the stimulator, which was not switched on)	Bilateral ST36 and PC6	Alternating 2/100 Hz	No
Gu et al. 2019 (37)	TEAS	Sham (stimulator output wire broken, no current delivered)	Bilateral ST36 and PC6	Alternating 2/100 Hz	No
Zhou et al. (38)	TEAS	No-sham	LI4, PC6, BL21, BL27, ST36, ST37	Alternating 20/100 Hz	Yes
Pei et al. (20)^c^	EA	Sham (no skin penetration, no current)	Bilateral PC6, ST36, ST37	Fixed 50 Hz	Yes^d^
Guan et al. (40)	EA	Sham (adhesive surface electrodes at the same acupoints without needle insertion; the device was switched on with the indicator light visible to the patient but delivered no current)	Bilateral PC6, LI4, ST36, LI11	Alternating 2/100 Hz	Yes
Jung et al. (21)	EA	No-sham	Bilateral ST36, SP6, LI4, TE6, LV3, LI11; unilateral GV20, EX-HN3, GV26, CV24	Fixed 100 Hz	No
Cai et al. (22)	EA	Sham (same needling setup without current)	Bilateral ST36, SP6, LI4	Fixed 10 Hz	Yes
Ding and Huang (23)	EA	Sham (electrode pads at non-acupoints 2 cm from the true points, without skin penetration; lead wires severed so that no current was delivered)	GV20 (single midline point); PC6 (unilateral); ST25 and ST36 (bilateral)	Alternating 2/100 Hz	No
Wang et al. (41)	TEAS	No-sham (parenteral nutrition alone, without acupoint stimulation)	Bilateral ST36, PC6, LI4	Alternating 2/100 Hz	No
Huang and Li (24)	TEAS	No-sham (fast-track nursing alone, without acupoint stimulation)	LI4, PC6, ST36, SP10, SP6; additional points individualized	Fixed 10 Hz	Yes
Shi et al. (39)	TEAS	Sham (same transcutaneous procedure; stimulator not switched on)	ST36, PC6, LI4	Alternating 2/100 Hz	Yes

EA, electroacupuncture; ERAS, enhanced recovery after surgery; Hz, hertz; TEAS, transcutaneous electrical acupoint stimulation; BL, Bladder; CV, Conception Vessel; EX-HN, Extra points of Head and Neck; GV, Governor Vessel; LI, Large Intestine; LV, Liver; PC, Pericardium; SP, Spleen; ST, Stomach; TE, Triple Energizer.

Qualifying domains supporting each ERAS classification are listed in Supplementary Table S7. Stimulation parameters, delivery method, treatment schedule and practitioner background are given in Supplementary Table S2.

a Although termed “transcutaneous electroacupuncture” in the report, the intervention was classified as TEAS because stimulation was delivered through surface electrodes without needle insertion.

b The report interchanged the labels for ST36 and PC6; acupoints were coded according to the anatomical descriptions, while the reported frequency assignments were retained.

c Three-arm trial; the sham arm is shown here (standard-care comparison, Table 4, Panel B).

d Classified as ERAS-based although the perioperative pathway was called standard care in the original report, because five qualifying enhanced-recovery domains were described as implemented in both groups (Supplementary Table S7).

Three of these trials—Pei et al. (20), Guan et al. (40), and Cai et al. (22)—shared investigators and had related registration, ethics, or recruitment information. Their intervention protocols and study characteristics differed, but the published information alone could not exclude participant overlap. Investigator correspondence indicated that the randomized samples in the three reports were mutually non-overlapping. No unpublished outcome data or participant-level screening or randomization records were obtained. The three trials were therefore retained as independent studies, and because participant-level source records were unavailable for independent verification, a conservative sensitivity analysis retaining only Pei et al. among these three related trials was also performed. Detailed study-family, registration, ethics, and recruitment information is provided in Supplementary Table S6.

#### Trials included in the narrative synthesis only

Two additional randomized trials met the eligibility criteria but were included only in the narrative synthesis because the relevant gastrointestinal recovery outcomes were presented graphically, without numerical summaries suitable for pooling; numerical data requested from the corresponding authors were not provided. Guo et al. (42) reported that 102 participants were randomized to transcutaneous electrical acupoint stimulation, sham stimulation, or control and found no statistically significant difference in time to first flatus among the three groups; group-specific analyzed numbers for this outcome were not reported. He et al. (26) randomized 184 participants equally to conventional care, low-frequency electrical acupoint stimulation added to conventional care, fast-track surgery, or fast-track surgery plus stimulation; seven participants were removed after randomization, leaving 177 analyzed. Compared with conventional care, stimulation significantly shortened both time to first flatus and time to first defecation. Within the fast-track pathway, adding stimulation significantly shortened time to first flatus, whereas time to first defecation did not differ significantly from fast-track surgery alone. Outcome-specific risk-of-bias assessments for these results are reported in Supplementary Tables S3, S4.

### Overall effects of EAS on gastrointestinal recovery and LOS

The main results are presented in Figure 2. For the primary outcome, time to first flatus, 13 trials contributed 1,357 analyzed participants to the selected pairwise comparisons (EAS, *n* = 684; control, *n* = 673); these counts refer to the participants included in those comparisons rather than all participants randomized in the original trials. Time to first flatus was shorter with EAS (MD, −9.61 h; 95% CI, −12.81 to −6.42; *P* < 0.001; *τ*^2^ = 16.29, 95% Q-profile CI, 4.70 to 79.71; *Q* = 49.13, df = 12, *P* < 0.001; *I*^2^ = 82.6%), and the 95% prediction interval narrowly excluded the null (−18.97 to −0.25).

**Figure 2:**
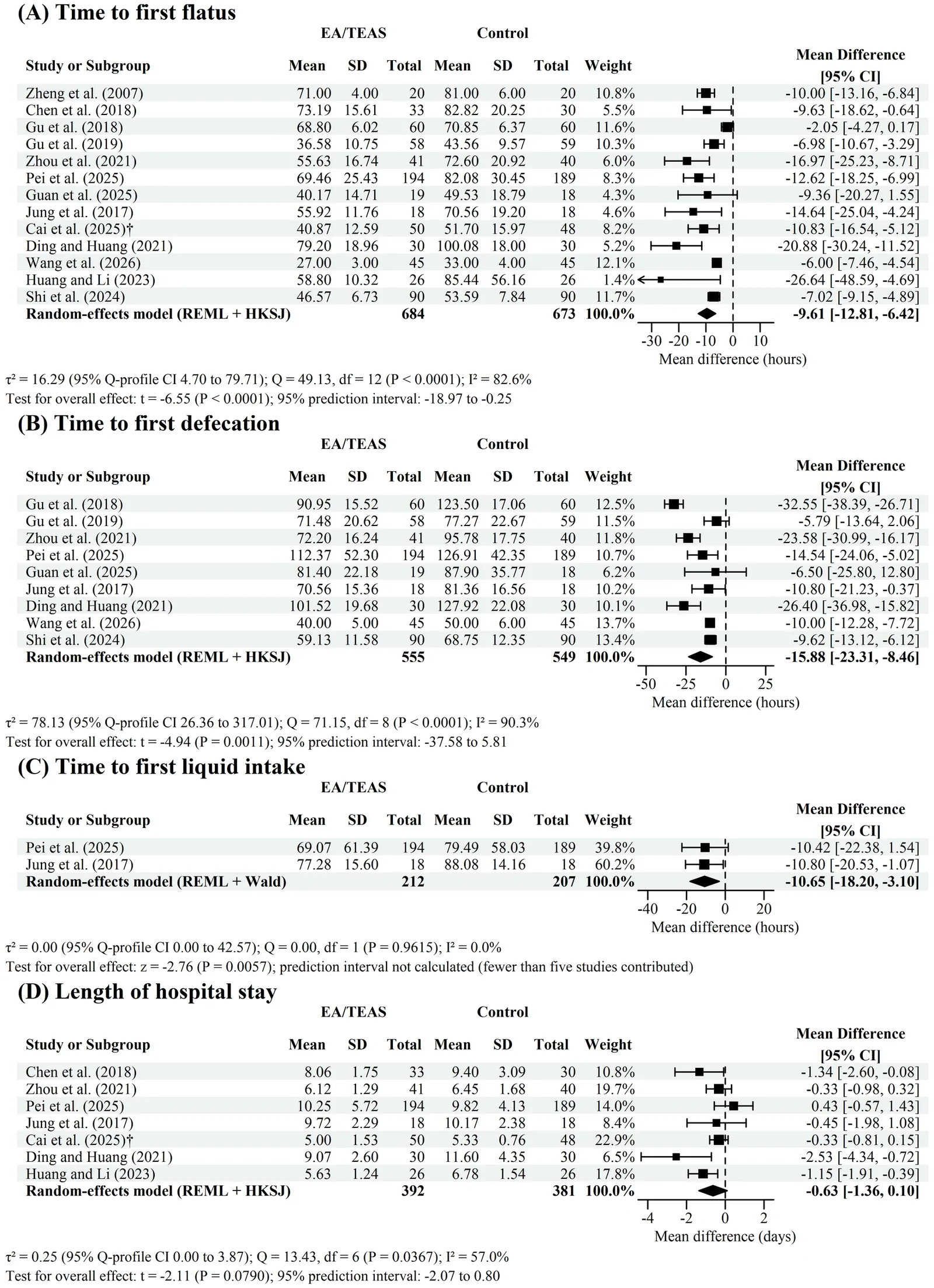
Forest plots of the effects of electrical acupoint stimulation on gastrointestinal recovery and length of hospital stay. **(A)** Time to first flatus; **(B)** time to first defecation; **(C)** time to first liquid intake; and **(D)** length of hospital stay. Diamonds represent pooled mean differences and their 95% confidence intervals. Between-study variance (*τ*^2^) was estimated using restricted maximum likelihood, and its 95% confidence interval was calculated using the Q-profile method. Hartung–Knapp–Sidik–Jonkman confidence intervals were used when *τ*^2^ was greater than zero and more than two trials contributed; otherwise, Wald-type confidence intervals were used. Ninety-five percent prediction intervals were calculated for outcomes to which at least five trials contributed. Study weights are inverse-variance random-effects weights. †For Cai et al. (22), means and standard deviations were estimated from reported medians and interquartile ranges using the method of Wan et al. (28). CI, confidence interval; EA, electroacupuncture; HKSJ, Hartung–Knapp–Sidik–Jonkman; REML, restricted maximum likelihood; SD, standard deviation; TEAS, transcutaneous electrical acupoint stimulation.

Pei et al. (20), the only trial reporting an extractable hazard ratio for time to first flatus, also found earlier flatus with electroacupuncture than with sham electroacupuncture in a Cox model adjusted for enrollment sites and family history of gastric cancer (hazard ratio, 1.56; 95% CI, 1.27–1.91; *P* < 0.001), directionally consistent with the mean-difference analysis.

For time to first defecation, nine trials contributed 1,104 analyzed participants (EAS, *n* = 555; control, *n* = 549). Time to first defecation was shorter with EAS (MD, −15.88 h; 95% CI, −23.31 to −8.46; *P* = 0.001; *τ*^2^ = 78.13, 95% Q-profile CI, 26.36 to 317.01; *Q* = 71.15, df = 8, *P* < 0.001; *I*^2^ = 90.3%), although the 95% prediction interval included the null (−37.58 to 5.81).

For time to first liquid intake, two trials contributed 419 analyzed participants (EAS, *n* = 212; control, *n* = 207). Time to first liquid intake was shorter with EAS (MD, −10.65 h; 95% CI, −18.20 to −3.10; *P* = 0.006; *τ*^2^ = 0, 95% Q-profile CI, 0.00 to 42.57; *Q* = 0.002, df = 1, *P* = 0.96; *I*^2^ = 0%). Under the conditional inference rule, Wald-type inference was used because only two trials contributed and *τ*^2^ was estimated as 0; no prediction interval was calculated because fewer than five trials contributed.

For LOS, seven trials contributed 773 analyzed participants (EAS, *n* = 392; control, *n* = 381). The difference was not statistically significant (MD, −0.63 days; 95% CI, −1.36 to 0.10; *P* = 0.079; *τ*^2^ = 0.25, 95% Q-profile CI, 0.00 to 3.87; *Q* = 13.43, df = 6, *P* = 0.04; *I*^2^ = 57.0%), and the 95% prediction interval included the null (−2.07 to 0.80).

The *post hoc* distributional plausibility assessment was applied to all study arms contributing to the selected pairwise comparisons for which a mean and standard deviation were available. The maximum fitted-normal probability below zero was 6.41% for time to first flatus (26 arms), occurring in the control arm of Huang and Li (24), and 1.58% for time to first defecation (18 arms). For time to first liquid intake, the corresponding probabilities in the electroacupuncture and sham-electroacupuncture arms of Pei et al. (20) were 13.03% and 8.54%, respectively, and the maximum for LOS was 3.66% (14 arms). In Cai et al. (22), for which means and standard deviations were derived from medians and interquartile ranges, the median positions within the interquartile range in the electroacupuncture and sham-electroacupuncture arms, respectively, were 0.67 and 0.15 for time to first flatus and 0.50 and 0.00 for LOS (Supplementary Table S5). Separately, the two trials contributing to time to first liquid intake had markedly different reported variances; Jung et al. (21), which contributed 36 of the 419 analyzed participants, received 60.2% of the random-effects weight.

### Trial-defined prolonged postoperative ileus and related outcomes

Two trials reported prolonged POI using explicit but different trial-defined thresholds. Chen et al. (36) defined prolonged POI as failure to pass flatus for more than 5 postoperative days and reported events in 0 of 33 patients in the TEAS group and 3 of 30 (10.0%) in the no-treatment control group. Pei et al. (20) defined it as failure to pass flatus for more than 72 h and reported events in 77 of 194 patients (39.7%) in the EA group and 113 of 189 (59.8%) in the sham-EA group. Because these trial-defined thresholds were not clinically equivalent, the results were not pooled.

Other related clinical indicators were reported heterogeneously. Guan et al. (40) reported good tolerance of an oral enteral nutrition suspension in 16 of 19 participants (84.2%) in the EA group and 9 of 18 (50.0%) in the placebo-EA group (*P* < 0.05). Pei et al. (20) reported postoperative prokinetic use in 24 of 194 EA and 21 of 189 sham-EA participants, and Cai et al. (22) reported no between-group difference in pro-motility agent use without numerical data; neither report established such use as rescue treatment specifically for ileus. No included trial explicitly reported nasogastric-tube reinsertion, reinstitution of fasting, initiation or reinstitution of parenteral nutrition as rescue treatment specifically for ileus, or a composite clinical definition of POI. Postoperative nausea, vomiting, abdominal distension, and related gastrointestinal symptoms were reported heterogeneously in four trials (19, 37, 38, 41); Wang et al. (41) reported a grouped “adverse reactions” outcome, with an overall reported incidence of 3/45 in the stimulation group and 11/45 in the control group; whether these totals represented unique participants or could include more than one symptom in the same participant could not be determined from the report. These related outcomes were not pooled because their definitions and reporting formats were not sufficiently comparable.

### Safety assessment

Safety outcomes are summarized in Table 3 using participant-level counts and corresponding denominators where these could be reconstructed. Reporting of intervention-related adverse events and general postoperative complications was incomplete and heterogeneous across trials.

**Table 3:** Patient-level safety outcomes reported in the trials contributing to the quantitative synthesis.

Safety outcome/Study	Comparison	EAS, n/N	Control, n/N
Participants with ≥1 intervention-related adverse event			
Chen et al. (36)	TEAS/no-sham	1/33^a^	Not applicable^b^
Zhou et al. (38)	TEAS/no-sham	0/41^c^	Not applicable^b^
Pei et al. (20)	EA/sham	11/194	3/189
Jung et al. (21)	EA/no-sham	0/18^d^	Not applicable^b^
Serious intervention-related adverse events			
Chen et al. (36)	TEAS/no-sham	0/33	Not applicable^b^
Zhou et al. (38)	TEAS/no-sham	0/41^c^	Not applicable^b^
Pei et al. (20)	EA/sham	0/194	0/189
Jung et al. (21)	EA/no-sham	0/18^d^	Not applicable^b^
Ding and Huang (23)	EA/sham	0/30^e^	0/30^e^
Minor intervention-related reactions			
Chen et al. (36)—bruising at the electrode site	TEAS/no-sham	1/33	Not applicable^b^
Pei et al. (20)—subcutaneous hematoma	EA/sham	8/194	0/189
Pei et al. (20)—dizziness	EA/sham	1/194	1/189
Pei et al. (20)—nausea	EA/sham	1/194	0/189
Pei et al. (20)—sweating	EA/sham	1/194	0/189
Pei et al. (20)—fatigue	EA/sham	0/194	2/189
Clavien–Dindo grade ≥ II complications			
Zhou et al. (38)	TEAS/no-sham	1/41	4/40
Guan et al. (40)	EA/sham	3/19	4/18
Cai et al. (22)	EA/sham	0/50	3/48
Clavien–Dindo grade ≥ III complications			
Zhou et al. (38)	TEAS/no-sham	Not reconstructable^f^	Not reconstructable^f^
Guan et al. (40)	EA/sham	1/19	0/18
Cai et al. (22)	EA/sham	0/50	0/48
Intervention discontinuation due to an adverse event			
Chen et al. (36)	TEAS/no-sham	1/33^a^	Not applicable^b^
Gu et al. 2018 (19)	TEAS/sham	0/60^h^	0/60^h^
Zhou et al. (38)	TEAS/no-sham	0/41	1/41^i^
Guan et al. (40)	EA/sham	0/22^g^	0/22
Ding and Huang (23)	EA/sham	0/30^e^	0/30^e^

EA, electroacupuncture; EAS, electrical acupoint stimulation; TEAS, transcutaneous electrical acupoint stimulation.

Counts are numbers of participants, not events. Denominators are the populations for which the corresponding safety outcome could be reconstructed. For intervention discontinuation, randomized or allocated group sizes are used; denominators for the other outcomes correspond to the population in which that outcome was ascertained. “Not applicable” denotes a control group that received no device application, in which intervention-related device or needling events could not arise. Studies not listed under a safety outcome did not report usable participant-level data for that outcome. No statistical tests are shown because reporting definitions and ascertainment differed across trials.

Withdrawals were classified as adverse-event related only when the source report established that relationship. Gastrointestinal symptoms not attributed to EAS and the ungraded postoperative complication categories reported by Huang and Li (24), for which participant overlap was not reported, are described in the Results; Shi et al. (39) reported no usable adverse-event or postoperative-complication counts.

a The report retained the randomized group size of 33 in its analyses; the handling of the participant who withdrew is described in the Results and was not specified in the report.

b See the note above on control groups without device application.

c Zhou et al. (38) stated that no adverse event was reported.

d Jung et al. (21) stated that no complication or adverse event related to acupuncture was reported.

e Ding and Huang (23) stated that no dropout and no serious adverse reaction occurred in the 60-participant trial; with 30 participants in each randomized arm, this corresponds to 0/30 in each arm for serious adverse reactions and discontinuations.

f A Clavien–Dindo grade above II was a withdrawal criterion in Zhou et al. (38); such participants were not included in the analysis, so grade ≥ III incidence in the randomized cohort cannot be reconstructed.

g Seven participants were excluded from the analysis after randomization in Guan et al. (40); none of these exclusions was attributed to an adverse event, so the randomized group sizes are used as the denominators for discontinuation.

h All 180 randomized participants in Gu et al. 2018 (19) completed the trial.

i In Zhou et al. (38), one control participant withdrew because of pulmonary infection and had received no stimulation.

#### Intervention-related adverse events

Pei et al. (20) provided the most complete reporting: 11 of 194 participants in the EA group and 3 of 189 in the sham-EA group experienced at least one intervention-related adverse event, corresponding to 12 and 3 events, respectively; the difference mainly reflected subcutaneous hematoma at the needling sites (8/194 vs. 0/189). No serious intervention-related adverse event occurred in either group. Chen et al. (36) reported bruising at the wrist electrode site in 1 of 33 participants, who withdrew from the study, and stated that no severe adverse event occurred. Zhou et al. (38) and Jung et al. (21) reported no intervention-related adverse events, while Ding and Huang (23) stated that no dropout and no serious adverse reaction occurred in the trial as a whole. Usable participant-level data on intervention-related adverse events were not reported for the remaining trials. No serious intervention-related adverse event was reported where assessed.

#### General postoperative complications

General postoperative complications were reported using varying definitions and formats. Three trials reported Clavien–Dindo classifications. Grade ≥ II complications occurred in 1/41 vs. 4/40 participants in Zhou et al. (38), 3/19 vs. 4/18 in Guan et al. (40), and 0/50 vs. 3/48 in Cai et al. (22). Grade ≥ III complications occurred in 1/19 vs. 0/18 participants in Guan et al. and 0/50 vs. 0/48 in Cai et al.; these counts could not be reconstructed for Zhou et al. (38). The single grade IV event in Guan et al. was a pulmonary embolism in the EA group; the report did not attribute the event to electroacupuncture. Huang and Li (24) reported ungraded postoperative complications comprising wound infection (1/26 vs. 3/26), digestive-system symptoms (1/26 vs. 3/26), pulmonary infection (0/26 vs. 2/26), and lower-limb deep-vein thrombosis (1/26 vs. 2/26); participant overlap across these categories was not reported, so a unique participant-level complication total could not be derived. Because definitions and reporting formats varied, these outcomes were not pooled.

### Sensitivity analysis

Sensitivity analyses are summarized in Table 4, with complete leave-one-out analyses presented in Supplementary Table S9. Leave-one-out analyses for outcomes with at least three contributing trials showed that time to first flatus and time to first defecation remained statistically significant after omission of any single trial, with pooled estimates ranging from −10.20 to −8.71 h and from −17.21 to −13.36 h, respectively. For time to first flatus, the prediction interval included the null in 8 of the 13 leave-one-out analyses. For LOS, the pooled estimate remained in the same direction after every omission but reached statistical significance only when the largest trial, Pei et al. (20), was omitted (MD, −0.76 days; 95% CI, −1.47 to −0.06; *P* = 0.038). Heterogeneity was most influenced by Gu et al. 2018 (19), the trial for which the reported time unit required interpretation; omitting that trial reduced *I*^2^ from 82.6% to 68.4% for time to first flatus and from 90.3% to 80.1% for time to first defecation.

**Table 4:** Sensitivity analyses.

Scenario	Time to first flatus, hours MD (95% CI)	Time to first defecation, hours MD (95% CI)	Time to first liquid intake, hours MD (95% CI)	Length of hospital stay, days MD (95% CI)
Panel A. Sensitivity to the between-study variance estimator and the confidence interval method				
Primary analysis: REML with the conditional inference rule	−9.61 (−12.81 to −6.42) *k* = 13; *τ*^2^ = 16.29; *I*^2^ = 82.6%; *P* < 0.001	−15.88 (−23.31 to −8.46) *k* = 9; *τ*^2^ = 78.13; *I*^2^ = 90.3%; *P* = 0.001	−10.65 (−18.20 to −3.10) *k* = 2; *τ*^2^ = 0.00; *I*^2^ = 0.0%; *P* = 0.006	−0.63 (−1.36 to 0.10) *k* = 7; *τ*^2^ = 0.25; *I*^2^ = 57.0%; *P* = 0.079
REML, Wald-type confidence interval	−9.61 (−12.41 to −6.81) *k* = 13; *τ*^2^ = 16.29; *I*^2^ = 82.6%; *P* < 0.001	−15.88 (−22.34 to −9.42) *k* = 9; *τ*^2^ = 78.13; *I*^2^ = 90.3%; *P* < 0.001	−10.65 (−18.20 to −3.10) *k* = 2; *τ*^2^ = 0.00; *I*^2^ = 0.0%; *P* = 0.006	−0.63 (−1.16 to −0.11) *k* = 7; *τ*^2^ = 0.25; *I*^2^ = 57.0%; *P* = 0.019
REML, modified Hartung–Knapp with the *ad hoc* variance correction	−9.61 (−12.81 to −6.42) *k* = 13; *τ*^2^ = 16.29; *I*^2^ = 82.6%; *P* < 0.001	−15.88 (−23.48 to −8.28) *k* = 9; *τ*^2^ = 78.13; *I*^2^ = 90.3%; *P* = 0.001	−10.65 (−59.59 to 38.29) *k* = 2; *τ*^2^ = 0.00; *I*^2^ = 0.0%; *P* = 0.221	−0.63 (−1.36 to 0.10) *k* = 7; *τ*^2^ = 0.25; *I*^2^ = 57.0%; *P* = 0.079
DerSimonian–Laird, Wald-type confidence interval	−9.21 (−11.62 to −6.81) *k* = 13; *τ*^2^ = 10.63; *I*^2^ = 75.6%; *P* < 0.001	−15.90 (−21.95 to −9.85) *k* = 9; *τ*^2^ = 66.63; *I*^2^ = 88.8%; *P* < 0.001	−10.65 (−18.20 to −3.10) *k* = 2; *τ*^2^ = 0.00; *I*^2^ = 0.0%; *P* = 0.006	−0.63 (−1.14 to −0.11) *k* = 7; *τ*^2^ = 0.24; *I*^2^ = 55.3%; *P* = 0.017
Paule–Mandel, Wald-type confidence interval	−9.69 (−12.59 to −6.80) *k* = 13; *τ*^2^ = 17.85; *I*^2^ = 83.9%; *P* < 0.001	−15.89 (−22.20 to −9.58) *k* = 9; *τ*^2^ = 73.78; *I*^2^ = 89.7%; *P* < 0.001	−10.65 (−18.20 to −3.10) *k* = 2; *τ*^2^ = 0.00; *I*^2^ = 0.0%; *P* = 0.006	−0.66 (−1.27 to −0.05) *k* = 7; *τ*^2^ = 0.41; *I*^2^ = 68.2%; *P* = 0.034
Sidik–Jonkman, Wald-type confidence interval	−9.95 (−13.19 to −6.71) *k* = 13; *τ*^2^ = 24.12; *I*^2^ = 87.5%; *P* < 0.001	−15.89 (−22.24 to −9.53) *k* = 9; *τ*^2^ = 75.08; *I*^2^ = 89.9%; *P* < 0.001	−10.65 (−18.20 to −3.10) *k* = 2; *τ*^2^ = 0.00; *I*^2^ = 0.0%; *P* = 0.006	−0.68 (−1.35 to −0.01) *k* = 7; *τ*^2^ = 0.55; *I*^2^ = 74.0%; *P* = 0.048
Maximum likelihood, Wald-type confidence interval	−9.47 (−12.12 to −6.82) *k* = 13; *τ*^2^ = 13.95; *I*^2^ = 80.2%; *P* < 0.001	−15.90 (−22.00 to −9.80) *k* = 9; *τ*^2^ = 67.92; *I*^2^ = 88.9%; *P* < 0.001	−10.65 (−18.20 to −3.10) *k* = 2; *τ*^2^ = 0.00; *I*^2^ = 0.0%; *P* = 0.006	−0.59 (−1.04 to −0.15) *k* = 7; *τ*^2^ = 0.13; *I*^2^ = 40.4%; *P* = 0.008
Common-effect (fixed-effect) model	−6.66 (−7.57 to −5.74) *k* = 13; *τ*^2^ = 0 by assumption; *I*^2^ not reported; *P* < 0.001	−12.72 (−14.36 to −11.07) *k* = 9; *τ*^2^ = 0 by assumption; *I*^2^ not reported; *P* < 0.001	−10.65 (−18.20 to −3.10) *k* = 2; *τ*^2^ = 0 by assumption; *I*^2^ not reported; *P* = 0.006	−0.52 (−0.82 to −0.21) *k* = 7; *τ*^2^ = 0 by assumption; *I*^2^ not reported; *P* < 0.001
Panel B. Sensitivity to study inclusion, data interpretation and comparator choice				
Primary analysis (all eligible trials)	−9.61 (−12.81 to −6.42) *k* = 13; *τ*^2^ = 16.29; *I*^2^ = 82.6%; *P* < 0.001 95% PI −18.97 to −0.25	−15.88 (−23.31 to −8.46) *k* = 9; *τ*^2^ = 78.13; *I*^2^ = 90.3%; *P* = 0.001 95% PI −37.58 to 5.81	−10.65 (−18.20 to −3.10) *k* = 2; *τ*^2^ = 0.00; *I*^2^ = 0.0%; *P* = 0.006 95% PI NC	−0.63 (−1.36 to 0.10) *k* = 7; *τ*^2^ = 0.25; *I*^2^ = 57.0%; *P* = 0.079 95% PI −2.07 to 0.80
Excluding results judged to be at high overall risk of bias	−11.68 (−20.15 to −3.22) *k* = 4; *τ*^2^ = 15.91; *I*^2^ = 66.3%; *P* = 0.022 95% PI NC	−15.16 (−40.79 to 10.48) *k* = 3; *τ*^2^ = 83.68; *I*^2^ = 79.0%; *P* = 0.126 95% PI NC	NE (*k* = 1)	−0.67 (−1.58 to 0.23) *k* = 6; *τ*^2^ = 0.36; *I*^2^ = 67.7%; *P* = 0.115 95% PI −2.46 to 1.11
Excluding trials without a sham comparator	−8.92 (−14.03 to −3.81) *k* = 7; *τ*^2^ = 20.95; *I*^2^ = 84.6%; *P* = 0.005 95% PI −21.23 to 3.39	−16.44 (−28.25 to −4.64) *k* = 6; *τ*^2^ = 109.21; *I*^2^ = 89.0%; *P* = 0.016 95% PI −45.79 to 12.90	NE (*k* = 1)	−0.63 (−4.18 to 2.91) *k* = 3; *τ*^2^ = 1.45; *I*^2^ = 85.5%; *P* = 0.523 95% PI NC
Excluding the trial for which means and standard deviations were converted from a median and interquartile range	−9.62 (−13.17 to −6.08) *k* = 12; *τ*^2^ = 18.43; *I*^2^ = 84.9%; *P* < 0.001 95% PI −19.71 to 0.47	—	—	−0.75 (−1.71 to 0.21) *k* = 6; *τ*^2^ = 0.43; *I*^2^ = 61.6%; *P* = 0.102 95% PI −2.69 to 1.19
Conservative analysis retaining only Pei et al. (20) among the three investigator- and institution-related trials	−9.74 (−13.63 to −5.84) *k* = 11; *τ*^2^ = 20.36; *I*^2^ = 87.1%; *P* < 0.001 95% PI −20.52 to 1.05	−16.51 (−24.61 to −8.41) *k* = 8; *τ*^2^ = 81.63; *I*^2^ = 91.6%; *P* = 0.002 95% PI −39.36 to 6.34	—	−0.75 (−1.71 to 0.21) *k* = 6; *τ*^2^ = 0.43; *I*^2^ = 61.6%; *P* = 0.102 95% PI −2.69 to 1.19
Excluding the largest trial	−9.38 (−12.85 to −5.90) *k* = 12; *τ*^2^ = 16.97; *I*^2^ = 83.8%; *P* < 0.001 95% PI −19.09 to 0.33	−16.03 (−24.63 to −7.43) *k* = 8; *τ*^2^ = 90.21; *I*^2^ = 92.0%; *P* = 0.003 95% PI −40.08 to 8.02	NE (*k* = 1)	−0.76 (−1.47 to −0.06) *k* = 6; *τ*^2^ = 0.16; *I*^2^ = 46.0%; *P* = 0.038 95% PI −2.01 to 0.48
Excluding Gu et al. 2018 (19), for which the reported time unit required interpretation	−10.11 (−12.95 to −7.26) *k* = 12; *τ*^2^ = 8.97; *I*^2^ = 68.4%; *P* < 0.001 95% PI −17.29 to −2.93	−13.36 (−19.49 to −7.22) *k* = 8; *τ*^2^ = 35.05; *I*^2^ = 80.1%; *P* = 0.001 95% PI −28.64 to 1.93	—	—
Standard-care arm substituted for the sham arm in Pei et al. (20)	−11.17 (−15.30 to −7.03) *k* = 13; *τ*^2^ = 34.53; *I*^2^ = 90.9%; *P* < 0.001 95% PI −24.62 to 2.29	−16.80 (−24.44 to −9.15) *k* = 9; *τ*^2^ = 82.80; *I*^2^ = 90.7%; *P* < 0.001 95% PI −39.13 to 5.54	−12.49 (−19.89 to −5.10) *k* = 2; *τ*^2^ = 0.00; *I*^2^ = 0.0%; *P* < 0.001 95% PI NC	−0.64 (−1.33 to 0.05) *k* = 7; *τ*^2^ = 0.19; *I*^2^ = 48.6%; *P* = 0.062 95% PI −1.90 to 0.62

CI, confidence interval; MD, mean difference; NC, not calculated; NE, not estimable; PI, prediction interval.

Effect estimates are mean differences with 95% confidence intervals; negative values favor electrical acupoint stimulation. The primary analyses used restricted maximum likelihood with Hartung–Knapp–Sidik–Jonkman intervals and tests when *τ*^2^ was greater than zero and more than two trials contributed, and Wald-type inference otherwise.

The common-effect model fixes *τ*^2^ at zero by assumption; *I*^2^ is not reported for that row, to avoid implying that the observed between-study heterogeneity was absent. *P* values throughout are for the test of the pooled mean difference and are not the *P* values from Cochran's *Q* test of heterogeneity.

Prediction intervals are shown in Panel B for analyses to which at least five trials contributed and are otherwise marked NC. A scenario is shown only for the outcomes to which it applies; an em dash indicates that the excluded trial did not contribute to that outcome, so the scenario would reproduce the primary analysis.

Where only one trial remained, no pooled estimate could be calculated (NE).

A conservative analysis retaining only Pei et al. (20) was performed to assess robustness to the shared investigator and institutional context of the three trials (Supplementary Table S6).

Complete leave-one-out analyses are presented in Supplementary Table S9; leave-one-out analysis was not applicable to time to first liquid intake because only two trials contributed.

Alternative between-study variance estimators and confidence-interval methods yielded similar random-effects point estimates for time to first flatus and time to first defecation, ranging from −9.95 to −9.21 h and from −15.90 to −15.88 h, respectively. For time to first flatus, the common-effect estimate was attenuated to −6.66 h (95% CI, −7.57 to −5.74). For time to first liquid intake, every between-study variance estimator returned *τ*^2^ = 0, and analyses applying the conditional inference rule produced the same estimate and confidence interval; the modified Hartung–Knapp sensitivity analysis, however, yielded an MD of −10.65 h (95% CI, −59.59 to 38.29; *P* = 0.221). For LOS, the point estimates were similar across random-effects estimators, but statistical significance varied across analytical specifications: the primary HKSJ analysis and the modified Hartung–Knapp analysis were not statistically significant, whereas the Wald-type random-effects analyses and the common-effect model yielded confidence intervals that excluded the null.

Among the study-inclusion sensitivity analyses, excluding results judged to be at high overall risk of bias left four trials for time to first flatus and three for time to first defecation. The reduction in time to first flatus remained statistically significant (MD, −11.68 h; 95% CI, −20.15 to −3.22; *P* = 0.022), whereas the estimate for time to first defecation was no longer statistically significant (MD, −15.16 h; 95% CI, −40.79 to 10.48; *P* = 0.126). Restricting the analyses to sham-controlled trials retained statistically significant reductions in time to first flatus (MD, −8.92 h; 95% CI, −14.03 to −3.81) and time to first defecation (MD, −16.44 h; 95% CI, −28.25 to −4.64). For time to first liquid intake, excluding high-risk results, restricting to sham-controlled trials, or excluding the largest trial each left only one contributing trial, so no pooled estimate could be calculated. Excluding Cai et al. (22), for which means and standard deviations were derived from medians and interquartile ranges, left the pooled estimate for time to first flatus statistically significant and that for LOS non-significant. In the conservative analysis retaining only Pei et al. (20) among the three investigator- and institution-related trials, time to first flatus (MD, −9.74 h; 95% CI, −13.63 to −5.84) and time to first defecation (MD, −16.51 h; 95% CI, −24.61 to −8.41) remained statistically significant, whereas LOS remained non-significant (MD, −0.75 days; 95% CI, −1.71 to 0.21).

When the standard-care arm of Pei et al. (20) was substituted for its sham arm, the pooled reductions remained statistically significant for time to first flatus (MD, −11.17 h; 95% CI, −15.30 to −7.03), time to first defecation (MD, −16.80 h; 95% CI, −24.44 to −9.15), and time to first liquid intake (MD, −12.49 h; 95% CI, −19.89 to −5.10), whereas LOS remained non-significant (MD, −0.64 days; 95% CI, −1.33 to 0.05). Heterogeneity for time to first flatus increased from *I*^2^ = 82.6% to 90.9%. The sham-controlled comparison remained the primary analysis.

### Exploratory subgroup analyses

The results of the exploratory subgroup analyses are presented in Table 5. None of the 12 primary Knapp–Hartung interaction tests was statistically significant. The limited numbers of trials, particularly in some subgroups, constrained the precision of the interaction analyses.

**Table 5:** Exploratory subgroup analyses by comparator design, perioperative care protocol, stimulation modality, and stimulation-frequency strategy.

Outcome	Subgroup	*k*	Analyzed *n* (I/C)	MD (95% CI)	*τ* ^2^	*I*^2^, %	CI method	*P* for pooled effect	*P* interaction, *Q*	*P* interaction, KH
Time to first flatus (h)	Comparator design								0.538	0.489
	Sham	7	501/494	−8.92 (−14.03 to −3.81)	20.95	84.6	HKSJ	0.005		
	No-sham	6	183/179	−10.73 (−16.20 to −5.25)	13.78	70.7	HKSJ	0.004		
	Perioperative care protocol								0.394	0.333
	ERAS	6	420/411	−11.01 (−15.82 to −6.19)	9.40	50.8	HKSJ	0.002		
	Conventional	7	264/262	−8.63 (−13.87 to −3.39)	21.03	88.4	HKSJ	0.007		
	Stimulation modality								0.017	0.063
	EA	5	311/303	−12.96 (−16.27 to −9.65)	0.00	0.0	Wald	<0.001		
	TEAS	8	373/370	−7.64 (−11.57 to −3.71)	10.85	82.6	HKSJ	0.002		
	Stimulation-frequency strategy								0.167	0.207
	Alternating	8	363/362	−8.62 (−13.25 to −3.99)	19.85	89.8	HKSJ	0.003		
	Fixed	5	321/311	−12.11 (−15.52 to −8.70)	0.00	0.0	Wald	<0.001		
Time to first defecation (h)	Comparator design								0.779	0.812
	Sham	6	451/446	−16.44 (−28.25 to −4.64)	109.21	89.0	HKSJ	0.016		
	No-sham	3	104/103	−14.62 (−33.54 to 4.31)	47.17	81.8	HKSJ	0.080		
	Perioperative care protocol								0.691	0.701
	ERAS	4	344/337	−14.45 (−25.95 to −2.95)	37.66	71.0	HKSJ	0.028		
	Conventional	5	211/212	−17.05 (−31.62 to −2.48)	124.47	92.7	HKSJ	0.031		
	Stimulation modality								0.940	0.908
	EA	4	261/255	−15.73 (−28.76 to −2.70)	31.71	47.9	HKSJ	0.031		
	TEAS	5	294/294	−16.22 (−30.19 to −2.24)	117.15	95.8	HKSJ	0.032		
	Stimulation-frequency strategy								0.481	0.644
	Alternating	7	343/342	−16.70 (−26.73 to −6.68)	101.83	93.5	HKSJ	0.007		
	Fixed	2	212/207	−12.84 (−19.87 to −5.81)	0.00	0.0	Wald	<0.001		
Length of hospital stay (days)	Comparator design								0.861	0.587
	Sham	3	274/267	−0.63 (−4.18 to 2.91)	1.45	85.5	HKSJ	0.523		
	No-sham	4	118/114	−0.78 (−1.57 to 0.02)	0.09	28.4	HKSJ	0.053		
	Perioperative care protocol								0.097	0.183
	ERAS	4	311/303	−0.39 (−1.31 to 0.53)	0.15	55.1	HKSJ	0.269		
	Conventional	3	81/78	−1.35 (−3.72 to 1.03)	0.20	25.3	HKSJ	0.135		
	Stimulation modality								0.598	0.520
	EA	4	292/285	−0.52 (−2.30 to 1.27)	0.67	70.6	HKSJ	0.426		
	TEAS	3	100/96	−0.84 (−2.19 to 0.51)	0.15	44.4	HKSJ	0.117		
	Stimulation-frequency strategy								0.525	0.585
	Alternating	2	71/70	−1.26 (−3.39 to 0.87)	1.94	80.0	Wald	0.246		
	Fixed	5	321/311	−0.54 (−1.39 to 0.31)	0.24	55.0	HKSJ	0.150		

CI, confidence interval; EA, electroacupuncture; ERAS, enhanced recovery after surgery; HKSJ, Hartung–Knapp–Sidik–Jonkman; I/C, intervention/control; k, number of contributing trials; KH, Knapp–Hartung; MD, mean difference; TEAS, transcutaneous electrical acupoint stimulation.

MD is expressed in hours for the recovery times and in days for length of hospital stay; *τ*^2^, a variance, is expressed in h^2^ and days^2^ respectively. Analyzed *n* refers to the participants contributing to the corresponding outcome analysis. Within every subgroup *τ*^2^ was estimated separately; confidence intervals and tests of pooled effects used the HKSJ method when *τ*^2^ exceeded zero and more than two trials contributed, and Wald-type inference otherwise, as indicated in the “CI method” column. Subgroup definitions follow the Methods and Table 2. The frequency-strategy classification was also consistent with the source-reported stimulation waveforms and schedules summarized in Supplementary Table S2.

*P* for pooled effect is the effect within that subgroup and is not a test of effect modification; subgroup-specific *I*^2^ and *τ*^2^ are descriptive. *P* interaction, KH is the primary test (random-effects meta-regression assuming a common *τ*^2^, Knapp–Hartung *F* test); *P* interaction, Q is a secondary conventional Cochran's *Q* test using subgroup-specific *τ*^2^. Interaction *P* values were exploratory and were not adjusted for multiple testing.

Perioperative care was classified from the co-interventions described as actually implemented in both randomized groups (Supplementary Table S7). Guan et al. (40) was the only trial that met exactly three qualifying domains. Reclassifying that trial as conventional care did not materially change the interaction tests for perioperative care (time to first flatus, KH *P* = 0.316; time to first defecation, KH *P* = 0.973); length of hospital stay was unaffected because that trial did not contribute to it.

Time to first liquid intake is not shown because only two trials contributed. Stimulation modality and stimulation-frequency strategy did not vary between them, and the comparator-design and perioperative-care classifications would each have produced single-trial strata.

#### Comparator design

For time to first flatus, the pooled estimates were −8.92 h (95% CI, −14.03 to −3.81; 7 trials) in sham-controlled trials and −10.73 h (95% CI, −16.20 to −5.25; 6 trials) in no-sham trials (*P* for interaction = 0.489). For time to first defecation, the corresponding estimates were −16.44 h (95% CI, −28.25 to −4.64; 6 trials) and −14.62 h (95% CI, −33.54 to 4.31; 3 trials), respectively (*P* for interaction = 0.812). For LOS, they were −0.63 days (95% CI, −4.18 to 2.91; 3 trials) and −0.78 days (95% CI, −1.57 to 0.02; 4 trials), respectively (*P* for interaction = 0.587).

#### Perioperative care protocol

For time to first flatus, the estimates were −11.01 h (95% CI, −15.82 to −6.19; 6 trials) in the ERAS subgroup and −8.63 h (95% CI, −13.87 to −3.39; 7 trials) in the conventional-care subgroup (*P* for interaction = 0.333). For time to first defecation, they were −14.45 h (95% CI, −25.95 to −2.95; 4 trials) and −17.05 h (95% CI, −31.62 to −2.48; 5 trials), respectively (*P* for interaction = 0.701). For LOS, they were −0.39 days (95% CI, −1.31 to 0.53; 4 trials) and −1.35 days (95% CI, −3.72 to 1.03; 3 trials), respectively (*P* for interaction = 0.183).

#### Stimulation modality

For time to first flatus, the estimates were −12.96 h (95% CI, −16.27 to −9.65; 5 trials) with EA and −7.64 h (95% CI, −11.57 to −3.71; 8 trials) with TEAS. The secondary Cochran Q interaction test was nominally significant (*P* = 0.017), whereas the primary Knapp–Hartung interaction test was not (*P* = 0.063). For time to first defecation, the corresponding estimates were −15.73 h (95% CI, −28.76 to −2.70; 4 trials) and −16.22 h (95% CI, −30.19 to −2.24; 5 trials), respectively (*P* for interaction = 0.908). For LOS, they were −0.52 days (95% CI, −2.30 to 1.27; 4 trials) and −0.84 days (95% CI, −2.19 to 0.51; 3 trials), respectively (*P* for interaction = 0.520).

#### Stimulation-frequency strategy

For time to first flatus, the estimates were −8.62 h (95% CI, −13.25 to −3.99; 8 trials) with alternating-frequency stimulation and −12.11 h (95% CI, −15.52 to −8.70; 5 trials) with fixed-frequency stimulation (*P* for interaction = 0.207). For time to first defecation, they were −16.70 h (95% CI, −26.73 to −6.68; 7 trials) and −12.84 h (95% CI, −19.87 to −5.81; 2 trials), respectively (*P* for interaction = 0.644). For LOS, they were −1.26 days (95% CI, −3.39 to 0.87; 2 trials) and −0.54 days (95% CI, −1.39 to 0.31; 5 trials), respectively (*P* for interaction = 0.585).

### Risk of bias assessment

Outcome-specific RoB 2 assessments were conducted for the 31 study–outcome results from the 13 trials contributing to the four quantitative outcomes and for five additional results from the two trials included in the narrative synthesis only, with the effect of assignment to intervention as the effect of interest throughout (Supplementary Tables S3, S4). Among the 31 results contributing to the quantitative synthesis, 17 were judged to be at high risk of bias and 14 raised some concerns; none was judged to be at low risk overall. Among the five narrative-synthesis results, four were at high risk and one raised some concerns.

For the primary outcome, time to first flatus, nine of 13 results were at high risk of bias and four raised some concerns (Figure 3). For time to first defecation, six of nine results were at high risk and three raised some concerns (Supplementary Figure S1). For time to first liquid intake, one of two results was at high risk and one raised some concerns. For LOS, one of seven results was at high risk and six raised some concerns (Supplementary Figure S2).

**Figure 3:**
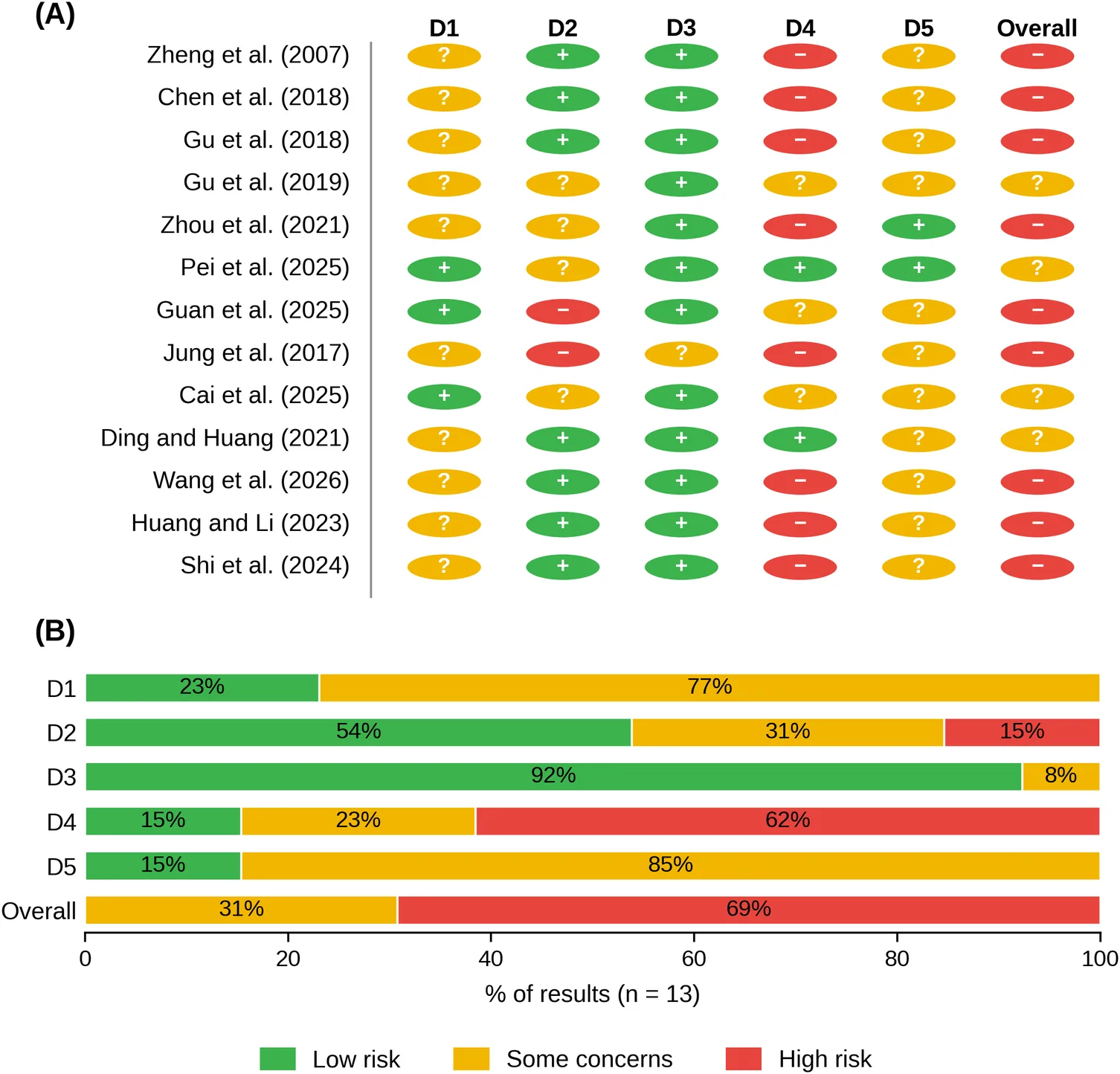
Risk-of-bias assessment for the primary outcome, time to first flatus. **(A)** Traffic-light plot showing the domain-level and overall risk-of-bias judgments for each of the 13 results contributing to the analysis; **(B)** summary plot showing the distribution of judgments across domains. Assessments were made with the Cochrane Risk of Bias 2 tool for each study–outcome result, with the effect of assignment to intervention as the effect of interest. Corresponding plots for time to first defecation and length of hospital stay are shown in Supplementary Figures S1, S2. No corresponding plot is presented for time to first liquid intake because only two results contributed; their judgments are reported in Supplementary Table S3. D1, randomization process; D2, deviations from intended interventions; D3, missing outcome data; D4, measurement of the outcome; D5, selection of the reported result. Full signaling-question responses and supporting evidence are provided in Supplementary Table S4.

Across the 31 quantitative results, Domain 1 was judged to be at low risk for eight results and raised some concerns for 23, principally because allocation concealment could not be verified. Domain 2 was at low risk for 14 results, raised some concerns for 11, and was at high risk for six; all six high-risk judgments arose from Guan et al. (40), which excluded seven of 44 randomized participants from the analyses, and Jung et al. (21), which excluded four of 40 randomized participants. Domain 3 was at low risk for 27 results and raised some concerns for four, all from Jung et al. (21). Domain 4 accounted for most high-risk domain judgments: 14 results were at high risk, 10 raised some concerns, and seven were at low risk. The high-risk judgments involved participant- or clinician-dependent gastrointestinal recovery milestones assessed under conditions in which knowledge of treatment assignment could plausibly influence outcome ascertainment. In Domain 5, seven results were at low risk and 24 raised some concerns, mainly because prospective registration, protocols, or analysis plans could not be verified.

Overall, high-risk judgments were driven predominantly by measurement of gastrointestinal recovery milestones under conditions susceptible to influence by treatment knowledge and, in Guan et al. (40) and Jung et al. (21), by analyses that excluded randomized participants; incomplete reporting of allocation concealment and prospective prespecification accounted for most judgments of some concerns.

### Certainty of evidence

The certainty of evidence for the quantitative syntheses is summarized in Table 6. The certainty for time to first flatus was rated as very low, with serious concerns regarding risk of bias, inconsistency, imprecision, and publication bias. Risk of bias was driven mainly by measurement of participant- or clinician-dependent recovery milestones under conditions susceptible to influence by treatment knowledge and by analyses that excluded randomized participants. Inconsistency was judged serious (*I*^2^ = 82.6%; *τ*^2^ = 16.29, 95% Q-profile CI, 4.70 to 79.71); although the 95% prediction interval narrowly excluded the null (−18.97 to −0.25), substantial between-trial heterogeneity remained. Imprecision was also judged serious because the 95% confidence interval represented an improvement of 6.42 to 12.81 h and therefore included effects both smaller and larger than the 8-hour threshold. The optimal information size was met using the median control-group SD (160 participants) but not using the largest observed control-group SD (1,548 participants); the imprecision judgment rested on the confidence interval crossing the threshold.

**Table 6:** Evidence profile and summary of findings.

Outcome	EAS/Control (Studies)	Risk of bias	Inconsistency	Indirectness	Imprecision	Publication bias	MD [95% CI]	GRADE certainty	Importance
Time to first flatus (hours)	684/673 (13 RCTs)	Serious^a^	Serious^b^	Not serious	Serious^c^	Serious^d^	−9.61 [−12.81, −6.42]	VERY LOW	Critical
Time to first defecation (hours)	555/549 (9 RCTs)	Serious^a^	Serious^e^	Not serious	Not serious^f^	Not assessed^g^	−15.88 [−23.31, −8.46]	LOW	Critical
Time to first liquid intake (hours)	212/207 (2 RCTs)	Serious^a^	Not serious^h^	Not serious	Serious^i^	Not assessed^g^	−10.65 [−18.20, −3.10]	LOW	Important
Length of hospital stay (days)	392/381 (7 RCTs)	Serious^a^	Not serious^j^	Not serious	Serious^k^	Not assessed^g^	−0.63 [−1.36, 0.10]	LOW	Important

CI, confidence interval; EAS, electrical acupoint stimulation; GRADE, Grading of Recommendations Assessment, Development and Evaluation; *I*^2^, inconsistency statistic; MD, mean difference; OIS, optimal information size; RCT, randomized controlled trial; SD, standard deviation.

Effect estimates are the primary random-effects estimates and correspond to Figure 2 and Table 4, Panel A. Certainty ratings apply only to the quantitative syntheses; the two trials included in the narrative synthesis only were not incorporated (Supplementary Tables S3, S4). Imprecision was assessed against pragmatic thresholds of 8 h for the gastrointestinal recovery milestones and 1 day for LOS, together with the optimal information size, as described in the Methods.

a Downgraded one level for risk of bias: none of the 31 contributing results was at low overall risk (17 high risk, 14 some concerns; Supplementary Tables S3, S4).

b Downgraded one level for inconsistency (*I*^2^ = 82.6%; *τ*^2^ = 16.29). The 95% prediction interval (−18.97 to −0.25) excluded the null only narrowly.

c Downgraded one level for imprecision: the 95% confidence interval included effects both smaller and larger than the 8-hour threshold.

d Downgraded one level for publication bias: Egger regression indicated funnel-plot asymmetry (*P* = 0.006), whereas the Begg–Mazumdar rank-correlation test was not statistically significant; the L0 trim-and-fill analysis attenuated the pooled estimate to −6.74 h (Supplementary Figure S3).

e Downgraded one level for inconsistency (*I*^2^ = 90.3%; *τ*^2^ = 78.13); the 95% prediction interval (−37.58 to 5.81) included the null.

f Not downgraded for imprecision: the entire 95% confidence interval lay at or beyond the 8-hour threshold and the optimal information size was met.

g Fewer than 10 trials contributed; small-study effects were not formally assessed.

h Not downgraded for inconsistency (*I*^2^ = 0%; *τ*^2^ = 0). With only two contributing trials, heterogeneity could not be estimated reliably, so this agreement is not itself evidence of consistency.

i Downgraded one level for imprecision: the 95% confidence interval crossed the 8-hour threshold and the optimal information size was not met.

j Not downgraded for inconsistency: heterogeneity was moderate (*I*^2^ = 57.0%; *τ*^2^ = 0.25 days^2^), with substantial overlap among the confidence intervals of the contributing trials; Cochran's Q was statistically significant (*Q* = 13.43, df = 6; *P* = 0.04), and the 95% prediction interval (−2.07 to 0.80 days) crossed the null.

k Downgraded one level for imprecision: the 95% confidence interval included both no effect and a reduction exceeding the 1-day threshold, although the optimal information size was met.

For time to first defecation, the certainty of evidence was rated as low after downgrading for serious risk of bias and serious inconsistency (*I*^2^ = 90.3%; *τ*^2^ = 78.13, 95% Q-profile CI, 26.36 to 317.01; 95% prediction interval, −37.58 to 5.81). Imprecision was not judged serious because the entire 95% confidence interval represented an improvement of 8.46 to 23.31 h, lying at or beyond the 8-hour threshold, and the optimal information size was met using both the median and largest observed control-group SDs. For time to first liquid intake, the certainty was rated as low after downgrading for serious risk of bias and serious imprecision; the 95% confidence interval represented an improvement of 3.10 to 18.20 h and therefore crossed the 8-hour threshold, while the optimal information sizes of 640 and 1,652 participants based on the median and largest observed control-group SDs, respectively, both exceeded the 419 participants analyzed. For LOS, the certainty was also rated as low owing to serious risk of bias and serious imprecision; the 95% confidence interval included both no effect and a reduction exceeding the 1-day threshold. The optimal information size was met using both the median and largest observed control-group SDs, so the imprecision judgment was based on the span of the confidence interval rather than information size. No serious concerns regarding indirectness were identified for any outcome.

Small-study effects were formally assessed only for time to first flatus, to which 13 trials contributed. The Egger regression test indicated funnel-plot asymmetry (t = −3.40, df = 11, *P* = 0.006), whereas the Begg–Mazumdar rank-correlation test was not statistically significant (Kendall's *τ* = −0.33, *P* = 0.129). The L0 trim-and-fill procedure selected right-side filling and added six filled-in study estimates (Supplementary Figure S3B); refitting the augmented dataset with REML and HKSJ inference yielded an attenuated MD of −6.74 h (95% CI, −10.52 to −2.96), compared with −9.61 h in the primary analysis. The four results with the smallest sampling variances were not those of the four largest trials, and their control-group coefficients of variation were lower than those of the remaining trials (7.4%–14.6% vs. 18.0%–65.7%), providing an alternative explanation related to within-study variance. Nevertheless, the observed asymmetry and attenuation in the trim-and-fill analysis were considered sufficient to judge publication bias as serious. Because the certainty for time to first flatus had already reached very low after downgrading for risk of bias, inconsistency, and imprecision, this additional domain judgment did not further change the overall certainty category. For the remaining outcomes, fewer than 10 trials contributed and small-study effects were not formally assessed.

In threshold sensitivity assessments, the imprecision judgment for time to first flatus was not serious at a 6-hour threshold, whereas that for time to first defecation became serious at a 12-hour threshold. The judgment for time to first liquid intake remained serious at all three thresholds examined.

## Discussion

### Main findings and clinical interpretation

The direction of the estimates was consistent for the two bowel-function milestones: all 13 trials reporting time to first flatus and all nine reporting time to first defecation favored EAS. Nevertheless, the trial-level estimates varied substantially in magnitude, ranging from approximately 2 to 27 h for time to first flatus and from approximately 6 to 33 h for time to first defecation. This directional consistency did not extend to length of hospital stay, for which individual trial estimates included both shorter and longer stays with EAS and the pooled estimate was not statistically significant. Taken together, these findings identify a consistent directional signal for earlier postoperative gastrointestinal recovery milestones after gastrectomy for gastric cancer, but not a confirmed clinical benefit.

The clinical importance of these earlier recovery milestones remains uncertain. Time to first flatus and defecation are commonly used indicators of postoperative bowel recovery, but they should not be equated with prevention of clinically important postoperative gastrointestinal dysfunction (5, 43). Only two trials reported prolonged POI using explicit thresholds, and their definitions were not sufficiently comparable for quantitative synthesis. Evidence for time to first liquid intake was limited to two trials, and this outcome captured the timing of initial liquid intake rather than subsequent tolerance or progression of oral intake. Thus, the available evidence does not establish that earlier bowel-function milestones translate into a lower incidence of prolonged POI, improved tolerance or progression of oral intake, reduced need for rescue treatment, or earlier hospital discharge.

### Reproducibility and sources of uncertainty

The directional signal for the two bowel-function milestones was not dependent on any single trial, but its magnitude and reproducibility were less stable. Time to first flatus and time to first defecation remained statistically significant after omission of any individual trial; however, the prediction interval for time to first flatus included the null in eight of the 13 leave-one-out analyses. Heterogeneity was also sensitive to individual studies, with omission of Gu et al. 2018 (19) reducing *I*^2^ for both time to first flatus and time to first defecation. In addition, when results judged to be at high overall risk of bias were excluded, the pooled estimate for time to first defecation was no longer statistically significant, whereas that for time to first flatus remained significant. The LOS finding was similarly sensitive to analytical and study-inclusion choices: the estimate became statistically significant only after omission of the largest trial in the leave-one-out analysis, and its statistical significance varied across analytical specifications.

The exploratory subgroup analyses did not identify a convincing study-level explanation for the observed heterogeneity. None of the 12 primary Knapp–Hartung interaction tests was statistically significant across comparator design, perioperative care protocol, stimulation modality, or stimulation-frequency strategy. Interpretation of all four comparisons is further limited because study-level characteristics varied together and several subgroups contained only a small number of trials, so the analyses could not separate the examined characteristics from accompanying differences in trial size or methodological quality. The only nominally significant secondary interaction was the Cochran Q test comparing EA with TEAS for time to first flatus, but this finding was not confirmed by the primary Knapp–Hartung test, indicating that it was sensitive to the inferential method. Comparator design also requires cautious interpretation: the absence of a significant interaction between sham-controlled and no-sham trials does not establish that expectation- or measurement-related bias was absent, because the comparison classified control design rather than successful masking, and sham acupuncture cannot necessarily be assumed to be physiologically inert (44, 45). Similarly, the ERAS subgroup analysis compared studies with different perioperative pathways and therefore could not directly determine whether EAS provides incremental benefit when added to a common standardized ERAS program. He et al. (26) provided the closest within-trial examination of this question: compared with conventional care, stimulation was associated with earlier first flatus and defecation, whereas within the fast-track pathway, adding stimulation was associated with earlier first flatus but time to first defecation did not differ significantly from fast-track surgery alone. These findings were at high overall risk of bias and were reported only graphically, limiting their interpretability. Overall, these subgroup findings should therefore be regarded as hypothesis-generating and do not provide convincing evidence that the observed association differs according to comparator design, perioperative care, stimulation modality, or frequency strategy.

For time to first flatus, the credibility of the pooled magnitude was further limited by funnel-plot asymmetry and by the sensitivity of the pooled estimate to assumptions about small-study effects. The Egger regression test indicated asymmetry, whereas the Begg–Mazumdar rank-correlation test was not statistically significant. Selective publication was not the only plausible explanation for this pattern: the four results with the smallest sampling variances were not those of the four largest trials, indicating that differences in within-study variance may also have contributed to the apparent precision pattern. In the trim-and-fill sensitivity analysis, the L0 procedure selected right-side filling and added six filled-in study estimates, attenuating the pooled mean difference from −9.61 to −6.74 h, a reduction of approximately 30%. The resulting point estimate was below the review's *post hoc* pragmatic 8-hour threshold, which was not a validated minimal important difference, indicating that the apparent magnitude of the association was sensitive to assumptions about small-study effects. Accordingly, the observed asymmetry and attenuation supported a serious concern for publication bias, although they do not establish selective publication as the sole cause of the pattern.

Beyond uncertainty in the magnitude of the association, the internal validity of the observed directional signal was also limited. None of the quantitative study–outcome results was judged to be at low overall risk of bias, and high-risk judgments were driven predominantly by measurement of participant- or clinician-dependent gastrointestinal recovery milestones under conditions in which knowledge of treatment assignment could plausibly influence ascertainment. Additional concerns arose from analyses that excluded randomized participants in two trials. The uniform direction of the milestone estimates should therefore not be interpreted as evidence of an unbiased causal effect. Safety evidence was also limited: no clear serious intervention-related safety signal was identified where safety was assessed, but incomplete and heterogeneous adverse-event reporting precludes firm conclusions about safety, particularly uncommon harms.

### Strengths and limitations

Several features of this review strengthen the interpretability of these findings despite the limitations of the underlying evidence. Eligibility was restricted to patients undergoing gastrectomy for gastric cancer, avoiding the pooling of EAS estimates across different abdominal operations, and trials combining EAS with separately prescribed traditional or acupoint-based treatments were excluded to isolate EAS within conventional or ERAS-based perioperative care. The search covered international databases as well as CNKI, Wanfang Data, and SinoMed, together with trial registries and citation searching. Reports were mapped to their underlying randomized trials, and potentially related reports were evaluated before being treated as independent evidence. Risk of bias was assessed separately for each study–outcome result, including those from trials that could contribute only to the narrative synthesis; certainty was evaluated by outcome using GRADE, and random-effects analyses incorporated prediction intervals where appropriate. Multiple sensitivity analyses further examined the influence of key analytical and study-selection decisions.

The generalizability and completeness of the evidence base were also limited. The quantitative evidence was geographically concentrated, with 12 of the 13 contributing trials conducted in China and one in Korea. Operative and oncologic characteristics needed to assess applicability and potential effect modification were incompletely reported: cancer stage could not be harmonized in three trials and was not reported in three, surgical approach and gastrectomy type were each unreported in five, reconstruction was unreported in six, and none of the 13 trials reported the extent of lymphadenectomy. These gaps limited assessment of whether associations differed according to operative characteristics such as the extent of gastric resection or reconstruction. Reporting completeness posed a separate limitation. Two additional eligible randomized trials, Guo et al. (42) and He et al. (26), could contribute only to the narrative synthesis because relevant gastrointestinal recovery outcomes were presented graphically and numerical summaries suitable for pooling could not be obtained despite requests to the authors.

The available summary data also imposed limitations on the synthesis of elapsed-time outcomes using means and standard deviations. In the *post hoc* distributional plausibility assessment, the fitted-normal probability below zero reached 6.41% in the control arm of Huang and Li (24) for time to first flatus, illustrating the limitations of the normal approximation in at least one contributing arm. Cai et al. (22) required conversion of medians and interquartile ranges to means and standard deviations for time to first flatus and LOS, introducing additional approximation into those estimates. A different concern arose for time to first liquid intake, for which the much smaller of the two contributing trials received the majority of the random-effects weight because of its comparatively small reported variance. These observations do not establish non-normality or determine the direction of any resulting bias, but they illustrate how the form and reported precision of the available summary data can affect quantitative synthesis. Consistent with these concerns and the methodological limitations discussed above, GRADE certainty was very low for time to first flatus and low for time to first defecation, time to first liquid intake, and LOS.

### Future research

Future randomized trials should evaluate EAS within a clearly prespecified perioperative pathway that is applied consistently to both randomized groups. When the specific effect of EAS is the question, the comparator should use a credible sham that supports masking while minimizing unintended active stimulation. Outcome assessment should be masked or objective where feasible, and analyses should preserve randomized assignment. In addition to gastrointestinal recovery milestones, trials should incorporate standardized, clinically defined outcomes such as prolonged POI, tolerance and progression of oral intake, and readiness for discharge, drawing where appropriate on outcome definitions and frameworks developed in broader gastrointestinal surgical populations (5, 46), with sample-size justification based on the chosen clinically relevant endpoint.

Future studies should also be prospectively registered with prespecified analysis plans and should provide complete numerical outcome data together with detailed operative and stimulation characteristics. Replication in more geographically diverse populations and healthcare settings is needed to assess whether the observed associations extend beyond the settings represented in the current evidence. Where optimization of the stimulation protocol is the primary question, head-to-head trials could directly compare modalities or frequency strategies while holding other treatment conditions as similar as possible.

## Conclusions

The current evidence indicates a consistent directional signal favoring EAS for early postoperative gastrointestinal recovery milestones in patients undergoing gastrectomy for gastric cancer, but the magnitude, reproducibility, and clinical importance of this association remain uncertain. Future randomized trials should incorporate masked or objective outcome assessment where feasible, standardized and clinically defined recovery endpoints, greater geographic diversity, and complete numerical reporting.

## Data Availability

The original contributions presented in the study are included in the article/Supplementary Material. The extraction workbook and analysis scripts were not deposited in a public repository; further inquiries can be directed to the corresponding author.

## Supplementary material

Supplementary Figure S1 Risk-of-bias assessment for time to first defecation. (A) Traffic-light plot showing the domain-level and overall risk-of-bias judgments for each of the nine results contributing to the analysis; (B) summary plot showing the distribution of judgments across domains. Assessments were made with the Cochrane Risk of Bias 2 tool for each study-outcome result, with the effect of assignment to intervention as the effect of interest. D1, randomization process; D2, deviations from intended interventions; D3, missing outcome data; D4, measurement of the outcome; D5, selection of the reported result. Full signaling-question responses and supporting evidence are provided in Supplementary Table S4.

Supplementary Figure S2 Risk-of-bias assessment for length of hospital stay. (A) Traffic-light plot showing the domain-level and overall risk-of-bias judgments for each of the seven results contributing to the analysis; (B) summary plot showing the distribution of judgments across domains. Assessments were made with the Cochrane Risk of Bias 2 tool for each study-outcome result, with the effect of assignment to intervention as the effect of interest. D1, randomization process; D2, deviations from intended interventions; D3, missing outcome data; D4, measurement of the outcome; D5, selection of the reported result. Full signaling-question responses and supporting evidence are provided in Supplementary Table S4.

Supplementary Figure S3 Assessment of small-study effects for time to first flatus. (A) Contour-enhanced funnel plot of the 13 trials contributing to the meta-analysis. Open circles denote observed trial estimates. Shaded regions denote the 90%, 95%, and 99% confidence regions centered on the null value, and the vertical dotted line indicates a mean difference of zero. (B) Contour-enhanced funnel plot after application of the trim-and-fill procedure using the L0 estimator. Gray-filled circles denote the six filled-in estimates added by the procedure; these estimates do not represent identified trials.
