# Supplementary material for: Electrical acupoint stimulation for gastrointestinal recovery after gastrectomy for gastric cancer: a systematic review and meta-analysis
Source: Front Surg. 2026 Sep 16;13:1915755. doi: 10.3389/fsurg.2026.1915755 (PMC13625762; doi:10.3389/fsurg.2026.1915755)
Supplement: Supplementary file 2 [file Table1.docx]

**Supplementary Table S1. Full search strategies for bibliographic databases and trial registries.**

| **Source** | **Date searched** | **Search strategy** | **Records retrieved** |
| --- | --- | --- | --- |
| PubMed | 1 August 2026 | ("Stomach Neoplasms"[MeSH Terms] OR "gastric cancer"[Title/Abstract] OR "stomach cancer"[Title/Abstract] OR "gastric tumor"[Title/Abstract] OR "gastrectomy"[Title/Abstract] OR "gastric resection"[Title/Abstract]) AND ("Acupuncture Therapy"[MeSH Terms] OR "Acupuncture"[MeSH Terms] OR "Electroacupuncture"[MeSH Terms] OR "electroacupuncture"[Title/Abstract] OR "acupoint*"[Title/Abstract] OR "transcutaneous electrical acupoint stimulation"[Title/Abstract]) AND ("Randomized Controlled Trial"[Publication Type] OR "Controlled Clinical Trial"[Publication Type] OR "randomized"[Title/Abstract] OR "placebo"[Title/Abstract]) | 54 |
| CENTRAL (via the Cochrane Library) | 1 August 2026 | Advanced Search, Search Manager #1 [mh "Stomach Neoplasms"] #2 ("gastric cancer" OR "stomach cancer" OR "gastric tumor" OR "gastrectomy" OR "gastric resection"):ti,ab,kw #3 #1 OR #2 #4 [mh "Acupuncture Therapy"] #5 ("electroacupuncture" OR acupoint* OR "transcutaneous electrical acupoint stimulation"):ti,ab,kw #6 #4 OR #5 #7 #3 AND #6 | 124 |
| Embase (embase.com; institutional proxy access) | 1 August 2026 | ('stomach neoplasm'/exp OR 'gastrectomy'/exp OR 'stomach cancer':ti,ab OR 'gastric cancer':ti,ab OR 'gastric neoplasm':ti,ab OR 'gastric tumor':ti,ab OR 'distal gastrectomy':ti,ab OR 'total gastrectomy':ti,ab OR 'subtotal gastrectomy':ti,ab OR 'gastric resection':ti,ab) AND ('acupuncture'/exp OR 'electroacupuncture'/exp OR 'acupuncture therapy':ti,ab OR 'electroacupuncture therapy':ti,ab OR 'electro needle acupuncture':ti,ab OR 'acupoint':ti,ab OR 'acupoints':ti,ab OR 'acupoint stimulation':ti,ab OR 'acupoint treatment':ti,ab OR 'transcutaneous electrical acupoint stimulation':ti,ab) AND ('randomized controlled trial'/exp OR 'clinical trial'/exp OR 'randomized':ti,ab OR 'placebo':ti,ab) | 154 |
| Scopus | 1 August 2026 | TITLE-ABS-KEY("stomach neoplasm" OR "stomach cancer" OR "gastric cancer" OR "gastric tumor" OR "gastrectomy" OR "gastric resection") AND TITLE-ABS-KEY("acupuncture" OR "electroacupuncture" OR "acupoint*" OR "transcutaneous electrical acupoint stimulation") AND TITLE-ABS-KEY("randomized controlled trial" OR "clinical trial" OR "randomized" OR "placebo") | 137 |
| China National Knowledge Infrastructure (CNKI; global web interface via cnki.net) | 1 August 2026 | Professional Search; Chinese Academic Journals only. #1: (TI='胃癌' OR TI='胃肿瘤' OR TI='胃恶性肿瘤' OR TI='胃切除' OR TI='胃切除术' OR TI='胃癌根治术' OR KY='胃癌' OR KY='胃肿瘤' OR KY='胃恶性肿瘤' OR KY='胃切除' OR KY='胃切除术' OR KY='胃癌根治术' OR AB='胃癌' OR AB='胃肿瘤' OR AB='胃恶性肿瘤' OR AB='胃切除' OR AB='胃切除术' OR AB='胃癌根治术'); #2: (TI='电针' OR TI='电针疗法' OR TI='电针刺激' OR TI='经皮穴位电刺激' OR TI='经皮穴位电刺激疗法' OR TI='穴位电刺激' OR TI='经皮电针' OR TI='TEAS' OR KY='电针' OR KY='电针疗法' OR KY='电针刺激' OR KY='经皮穴位电刺激' OR KY='经皮穴位电刺激疗法' OR KY='穴位电刺激' OR KY='经皮电针' OR KY='TEAS' OR AB='电针' OR AB='电针疗法' OR AB='电针刺激' OR AB='经皮穴位电刺激' OR AB='经皮穴位电刺激疗法' OR AB='穴位电刺激' OR AB='经皮电针' OR AB='TEAS'); #3: #1 AND #2. No study-design filter or automatic term expansion was applied. | 210 |
| Wanfang Data (searched via wanfangdata.com.cn using the Advanced Search function [高级检索]) | 1 August 2026 | Advanced Search; journal articles (期刊论文), Chinese literature (中文文献). #1 主题 = 胃癌 OR 胃肿瘤 OR 胃恶性肿瘤 OR 胃切除 OR 胃切除术 OR 胃癌根治术; #2 主题 = 电针 OR 电针疗法 OR 电针刺激 OR 经皮穴位电刺激 OR 经皮穴位电刺激疗法 OR 穴位电刺激 OR 经皮电针 OR TEAS; #3 #1 AND #2. Fuzzy matching (模糊) was applied to both lines; 中英文扩展 and 主题词扩展 were disabled. No study-design filter was applied. | 189 |
| SinoMed (China Biology Medicine, CBM; sinomed.ac.cn) | 12 August 2026 | Quick Search (快速检索), Chinese biomedical literature database. #1 general fields (常用字段) = 胃癌 OR 胃肿瘤 OR 胃恶性肿瘤 OR 胃切除 OR 胃切除术 OR 胃癌根治术 (144,728); #2 general fields = 电针 OR 电针疗法 OR 电针刺激 OR 经皮穴位电刺激 OR 经皮穴位电刺激疗法 OR 穴位电刺激 OR 经皮电针 OR 经皮电刺激 OR TEAS (32,629); #3 = #1 AND #2 (90). Supplementary subject-heading search (主题检索, unweighted with expansion [不加权:扩展]): #4 = 胃肿瘤 OR 胃切除术 (124,793); #5 = 电针疗法 OR 电针 OR 经皮神经电刺激疗法 OR 经皮神经电刺激 OR (针刺穴位 AND (电刺激 OR 电刺激疗法)) (25,739); #6 = #5 AND #4 (42); #7 = #6 OR #3 (91); #8 = #7 AND − 2026[日期] (91). No study-design filter was applied. The interface date limit − 2026[日期], which restricts retrieval to records up to and including 2026, was applied to lines #1, #2 and to the final combined set (#8) | 91 |
| ClinicalTrials.gov | 1 August 2026 | Expert Search. Search A: AREA[ConditionSearch]("Stomach Neoplasms" OR "Stomach Neoplasm" OR "Stomach Cancer" OR "Gastric Cancer" OR "Gastric Neoplasm" OR "Gastric Tumor") AND AREA[InterventionSearch](Acupuncture OR Electroacupuncture OR Acupoint OR TEAS OR "Transcutaneous electrical acupoint stimulation" OR "Transcutaneous electroacupuncture" OR "Electrical acupoint stimulation" OR "Acupoint electrical stimulation") AND AREA[StudyType](INTERVENTIONAL). Search B: AREA[BasicSearch](Gastrectomy OR "Gastric Resection" OR "Stomach Resection") AND AREA[InterventionSearch](Acupuncture OR Electroacupuncture OR Acupoint OR TEAS OR "Transcutaneous electrical acupoint stimulation" OR "Transcutaneous electroacupuncture" OR "Electrical acupoint stimulation" OR "Acupoint electrical stimulation") AND AREA[StudyType](INTERVENTIONAL). All study statuses were included, with no restrictions based on study results, phase, country, age, or sex. No outcome-related or randomized-trial terms were used. Results from the two searches were combined and deduplicated by NCT number (17 records before deduplication; 3 duplicates removed). | 14 |
| WHO International Clinical Trials Registry Platform (ICTRP) | 1 August 2026 | Standard Search: (gastric OR stomach OR gastrectomy) AND (acupuncture OR electroacupuncture OR acupoint OR TEAS). Supplementary Advanced Search A: Condition = Stomach Neoplasms OR Stomach Neoplasm OR Stomach Cancer OR Gastric Cancer OR Gastric Neoplasm OR Gastric Tumor; Intervention = Acupuncture OR Electroacupuncture OR Acupoint OR TEAS. Supplementary Advanced Search B: Title = Gastrectomy OR Gastric Resection OR Stomach Resection; Intervention = Acupuncture OR Electroacupuncture OR Acupoint OR TEAS. Quotation marks and truncation were not used. Automatic synonym searching was retained. No restrictions were applied according to recruitment status, results availability, phase, country, or registration date. No randomized-trial or outcome-related terms were used. Results from the three searches were combined and internally deduplicated using TrialID and registry identifiers listed in the Secondary ID field. | 185 |
| Chinese Clinical Trial Registry (ChiCTR; Chinese-language interface) | 1 August 2026 | To complement the English-language registry data available through the WHO ICTRP portal, ChiCTR was searched directly in Chinese using its native-language title fields. Each search combined one intervention term (电针, 电针疗法, 电针刺激, 经皮穴位电刺激, 经皮穴位电刺激疗法, 穴位电刺激, or 经皮电针) with one disease or procedure term (胃癌, 胃切除, 胃肿瘤, or 胃癌根治术) across the Registered title (注册题目) and Scientific title (正式科学名) fields. The field assignments were reversed in a second search. The Target disease field was left blank, and study type, recruitment status, and registration date were left unrestricted. Results from all searches were combined and deduplicated by ChiCTR registration number. | 10 |
| International Traditional Medicine Clinical Trial Registry (ITMCTR; English interface) | 1 August 2026 | English interface; Trial search, with expanded search fields displayed using "More." Because retrieval through the Intervention and Target disease fields was inconsistent during calibration, source-adapted searches were conducted using the Public title and Scientific title fields. The intervention terms electroacupuncture, TEAS, 电针, 经皮穴位电刺激, and 穴位电刺激 were paired separately with gastric, gastrectomy, 胃癌, and 胃切除, with each pair searched in both field directions. In addition, gastric cancer was paired with electroacupuncture and 经皮穴位电刺激 in both field directions as sensitivity searches. No restrictions were applied by registration status, study type, recruitment status, or registration date. Retrieved records were combined, deduplicated by ITMCTR registration number, and manually screened for gastric cancer or gastrectomy, electrical acupoint stimulation, randomized allocation, and other eligibility criteria. | 8 |

All sources except SinoMed were searched from inception through 1 August 2026. The SinoMed search was executed on 12 August 2026, while the review search cutoff remained 1 August 2026. No language restriction was applied; source-specific interface settings are given in the table. The searches were re-run in full on 1 August 2026 after the initial search of 16 March 2026. For the Chinese-language searches, CNKI was searched across the title, keyword and abstract fields and Wanfang Data using the Topic field; SinoMed was searched in the general fields and, additionally, using its controlled subject headings. No study-design filter was applied in any of the three databases.

Records retrieved are the counts after removal of duplicates arising within the same source, which occurred where a source was searched with more than one strategy or interface. ClinicalTrials.gov was searched with two expert searches that together returned 17 records, of which three were retrieved by both searches, leaving 14 unique registrations. The WHO International Clinical Trials Registry Platform returned 247 rows across the standard and two supplementary searches, corresponding to 185 unique trials. The Chinese Clinical Trial Registry returned 52 hits across 56 search combinations, corresponding to 10 unique registrations, and the International Traditional Medicine Clinical Trial Registry returned 54 cumulative hits corresponding to 8 unique registrations. The four English-language bibliographic databases together contributed 469 records, the three Chinese-language databases 490, and the four registries 217, giving 1,176 records at the identification stage of the PRISMA flow diagram (Figure 1). Duplicates arising across sources were removed subsequently.

Because the review team could not obtain access sufficient for a reproducible search, the database provider carried out the SinoMed search on our behalf on 12 August 2026 using the strategy reported above and supplied the complete line-by-line search history and exported records. Of the 91 SinoMed records, 69 matched records retrieved from other sources; 22 were new, of which 20 were excluded at title-and-abstract screening and two met the eligibility criteria. The database update dates in those records were checked, and none was later than 1 August 2026, so the later execution date did not introduce records beyond the review search cutoff.
