# Supplementary material for: Electrical acupoint stimulation for gastrointestinal recovery after gastrectomy for gastric cancer: a systematic review and meta-analysis
Source: Front Surg. 2026 Sep 16;13:1915755. doi: 10.3389/fsurg.2026.1915755 (PMC13625762; doi:10.3389/fsurg.2026.1915755)
Supplement: Supplementary file 3 [file Table2.docx]

**Supplementary Table S2. Detailed intervention delivery characteristics of the trials contributing to the quantitative synthesis.**

| **Study** | **Stimulation parameters** | **Needling or delivery method** | **Treatment schedule** | **Practitioner background** |
| --- | --- | --- | --- | --- |
| Zheng et al. (35) | Dense-disperse wave alternating between 2 and 100 Hz; 8–10 mA while the patient was awake, increased to 20–30 mA after induction of general anesthesia | Surface electrodes (Han’s LH-202); one electrode pair connecting LI4 and PC8 on the same side, and one pair on bilateral ST36. The control group received no stimulation | Continuous from 30 min before induction until the end of surgery; after postoperative PCEA was established, 30-min TEAS was given three times at 3-h intervals starting 3–4 h after the loading dose, then once daily on each of the following 2 days (5 postoperative sessions) | Not reported |
| Chen et al. (36) | ST36: 25 Hz, 0.5-ms pulse width, 2 s on and 3 s off; PC6: 100 Hz, 0.25-ms pulse width, 0.1 s on and 0.4 s off; 2–6 mA, below the pain threshold. Waveform not reported | Surface electrodes placed over PC6 and ST36 | 60 min, twice daily, from POD1 until first flatus or POD5 | Study doctors, all of whom had been trained in the technique at the Pace Translational Medical Research Center (Ningbo, China); the number, license and clinical experience of the operators were not reported |
| Gu et al. 2018 (19) | Dense-disperse wave at 2/100 Hz; pulse width 0.2–0.6 ms; highest intensity tolerated by the patient, generally 8–12 mA | Surface electrodes applied to bilateral ST36 and PC6 (Han's LH402). In the control group the electrodes were applied and connected to the stimulator in the same way, but the device was not switched on | Long-duration group: continuous from 1 h before induction until 30 min after the end of surgery, then 30 min every 8 h for 2 postoperative days. The short-duration group received stimulation during surgery only and did not contribute to the review analyses | Not reported |
| Gu et al. 2019 (37) | Dense-disperse wave at 2/100 Hz; 5–30 mA, titrated to a de qi sensation, in the active group; in the control group the output wires of the stimulator were severed and the intensity display was set to 20 mA | Surface electrode tabs applied to bilateral ST36 and PC6 and connected to the stimulator identically in both groups (Han’s LH-202); control participants were told in advance that this form of acupuncture may produce no sensation | Continuous from 30 min before anesthetic induction in the holding area until 30 min after the operation in the surgical intensive care unit, then 30 min at 08:00, 13:00 and 18:00 on each of the 2 postoperative days (7 sessions) | Research nurses based in the operating room and the surgical intensive care unit, all of whom had completed 1 year of training in TEAS and were qualified members of the research team |
| Zhou et al. (38) | 20/100 Hz, programmed as 20 Hz for 5 s followed by 100 Hz for 10 s; intensity at the patient-tolerated upper limit. Waveform not reported | Surface electrode pads applied over the selected acupoints | 30 min, twice daily, on POD1–3 (6 sessions) | Not reported. The report names the investigators responsible for allocation and for data recording, but does not describe who delivered the stimulation |
| Pei et al. (20) | Continuous wave at 50 Hz; intensity adjusted to elicit slight needle-handle shivering without pain | Active EA: Hwato disposable needles (0.30 × 40 mm) inserted through insulating adhesive pads to approximately 15 mm at PC6 and 30 mm at ST36/ST37; de qi elicited manually; paired electrodes attached transversely to the needle handles at bilateral ST36 and ST37 (SDZ-V stimulator, Suzhou Medical Instrument), so that PC6 was needled without current. Sham EA: blunt placebo needles (0.30 × 25 mm) placed at the same acupoints through adhesive pads without skin penetration, with treatment procedures and electrode placements matched to the active group but without electrical stimulation or needle manipulation | 30 min at 2, 24, 48, and 72 h postoperatively (4 sessions) | Nine licensed acupuncturists, each with more than 5 years of clinical experience, who received centralized training before the trial |
| Guan et al. (40) | Dense-disperse wave at 2/100 Hz; pulse width 0.2–0.6 ms; intensity within patient tolerance after de qi | Active EA: disposable sterile 0.30 × 40 mm needles inserted perpendicularly to prespecified depths (PC6 5–10 mm, LI4 5–8 mm, LI11 10–15 mm, ST36 10–20 mm); de qi obtained; electrodes connected between LI11 and ST36 on the same side (SDZ-V stimulator); needles retained 30 min. Sham: disposable adhesive surface electrodes applied at the same acupoints without needle insertion; the device was switched on with the indicator light visible to the patient but delivered no current, and patients were informed in advance that they might feel no sensation | 30 min once daily from 1 h after surgery until first flatus | Physicians of traditional Chinese medicine from the Department of Acupuncture and Moxibustion, holding the professional rank of associate chief physician or above |
| Jung et al. (21) | 100 Hz. Waveform, pulse width and current intensity not reported | Single-use sterile needles inserted to the muscle layer; extremity points inserted perpendicularly to about 20 mm; head points inserted obliquely to about 5 mm; electrical stimulation applied at bilateral ST36, SP6, LI4, and TE6 (ES-160, Ito Co., Ltd.) | 25–30 min once daily on POD1–5 (5 sessions) | Korean medicine doctors at the study center; the number, license and clinical experience of the operators were not reported |
| Cai et al. (22) | Continuous wave at 10 Hz; 2–5 mA, within the range tolerable to the patient | Disposable sterile needles inserted 2–4 cm at bilateral ST36, SP6, and LI4; electrical stimulation applied between ST36 and SP6 on the same side (Hwatuo SDZ-V); the sham used the same setup without current | 30 min at 2 h after surgery, then on the mornings of POD1 and POD2 (3 sessions) | Not reported. The report indicates only that the acupuncturists could not be blinded, and gives no qualification, training or experience |
| Ding and Huang (23) | Dense-disperse wave at 2/100 Hz; intensity adjusted to patient tolerance after de qi | Active EA: needles inserted at GV20 (2 mm), unilateral PC6 (10 mm), and bilateral ST25 and ST36 (20 mm each), with de qi elicited before stimulation; the electrical connection was made between bilateral ST25 and ST36 (KWD-808I). Sham: adhesive electrode pads placed at non-acupoints 2 cm from the true points, without skin penetration, with the lead wires severed so that no current was delivered | 20 min per session, once on the day before surgery and once daily on POD1–5 (6 sessions in total) | Physicians of traditional Chinese medicine holding the professional rank of attending physician or above |
| Wang et al. (41) | Dense-disperse wave at 2/100 Hz, the disperse component at 2 Hz and the dense component at 100 Hz; pulse width 0.1–0.2 ms; 8–12 mA | Surface electrodes applied over bilateral ST36, PC6, and LI4 (SD-RBT-SJD-4). The control group received parenteral nutrition alone without acupoint stimulation | 30 min per session, twice daily, for 1 week from POD1 | A practitioner described as experienced and proficient in the procedure; the profession, license, training and years of experience were not reported |
| Huang and Li (24) | Continuous wave at 10 Hz; 20 mA, adjusted according to the individual patient response | Surface electrode pads were applied firmly over the selected acupoints using a G6805-2a transcutaneous stimulator. LI4, PC6 and ST36 were used for analgesia and gastrointestinal recovery, and SP10, ST36 and SP6 for prevention of lower-limb deep-vein thrombosis; additional points could be individualized according to the patient’s constitution and reaction points. The control group received the same fast-track nursing program without acupoint stimulation | 20 min per session, twice daily, on POD1–3 (6 sessions) | Not reported |
| Shi et al. (39) | Dense-disperse wave at 2/100 Hz; pulse width 0.2–0.6 ms; maximum current 8–12 mA | Transcutaneous stimulation using an LH402 device at ST36, PC6 and LI4; the control group underwent the same procedure with the stimulator not switched on | 30 min every 12 h from 1 day before surgery through POD3 | Not reported |

Abbreviations: EA, electroacupuncture; POD, postoperative day; TEAS, transcutaneous electrical acupoint stimulation.

Interventions are reported in the format recommended by the STRICTA extension of the CONSORT statement. Stimulation waveform, frequency, pulse width or duty cycle, and current intensity are given in this table where the source report specified them; unreported parameters were not inferred, and waveform was not inferred from the frequency setting. Several Chinese-language reports described the alternating waveform as 疏密波; published English reports rendered this waveform as both dense-disperse and disperse-dense wave, and for consistency these are standardized here as dense-disperse wave. Proprietary device and manufacturer names are given as spelled in each source report. The acupoints used, the frequency-strategy classification and the design of each comparator are given in Table 2; the perioperative co-interventions received by both groups of each trial are given in Supplementary Table S7.
