# Supplementary material for: Electrical acupoint stimulation for gastrointestinal recovery after gastrectomy for gastric cancer: a systematic review and meta-analysis
Source: Front Surg. 2026 Sep 16;13:1915755. doi: 10.3389/fsurg.2026.1915755 (PMC13625762; doi:10.3389/fsurg.2026.1915755)
Supplement: Supplementary file 4 [file Table3.docx]

**Supplementary Table S3. Outcome-specific risk-of-bias judgments for each study–outcome result.**

Panel A. Results contributing to the quantitative meta-analyses (n = 31)

| **Study (comparison)** | **Outcome** | **D1** | **D2** | **D3** | **D4** | **D5** | **Overall** |
| --- | --- | --- | --- | --- | --- | --- | --- |
| Zheng et al. (2007) | Time to first flatus | Some concerns | Low risk | Low risk | High risk | Some concerns | High risk |
| Chen et al. (2018) | Time to first flatus | Some concerns | Low risk | Low risk | High risk | Some concerns | High risk |
| Chen et al. (2018) | Length of hospital stay | Some concerns | Low risk | Low risk | Some concerns | Some concerns | Some concerns |
| Gu et al. (2018) | Time to first flatus | Some concerns | Low risk | Low risk | High risk | Some concerns | High risk |
| Gu et al. (2018) | Time to first defecation | Some concerns | Low risk | Low risk | High risk | Some concerns | High risk |
| Gu et al. (2019) | Time to first flatus | Some concerns | Some concerns | Low risk | Some concerns | Some concerns | Some concerns |
| Gu et al. (2019) | Time to first defecation | Some concerns | Some concerns | Low risk | Some concerns | Some concerns | Some concerns |
| Zhou et al. (2021) | Time to first flatus | Some concerns | Some concerns | Low risk | High risk | Low risk | High risk |
| Zhou et al. (2021) | Time to first defecation | Some concerns | Some concerns | Low risk | High risk | Low risk | High risk |
| Zhou et al. (2021) | Length of hospital stay | Some concerns | Some concerns | Low risk | Some concerns | Low risk | Some concerns |
| Pei et al. (2025) | Time to first flatus | Low risk | Some concerns | Low risk | Low risk | Low risk | Some concerns |
| Pei et al. (2025) | Time to first defecation | Low risk | Some concerns | Low risk | Low risk | Low risk | Some concerns |
| Pei et al. (2025) | Time to first liquid intake | Low risk | Some concerns | Low risk | Low risk | Low risk | Some concerns |
| Pei et al. (2025) | Length of hospital stay | Low risk | Some concerns | Low risk | Low risk | Low risk | Some concerns |
| Guan et al. (2025) | Time to first flatus | Low risk | High risk | Low risk | Some concerns | Some concerns | High risk |
| Guan et al. (2025) | Time to first defecation | Low risk | High risk | Low risk | Some concerns | Some concerns | High risk |
| Jung et al. (2017) | Time to first flatus | Some concerns | High risk | Some concerns | High risk | Some concerns | High risk |
| Jung et al. (2017) | Time to first defecation | Some concerns | High risk | Some concerns | High risk | Some concerns | High risk |
| Jung et al. (2017) | Time to first liquid intake | Some concerns | High risk | Some concerns | High risk | Some concerns | High risk |
| Jung et al. (2017) | Length of hospital stay | Some concerns | High risk | Some concerns | Some concerns | Some concerns | High risk |
| Cai et al. (2025) | Time to first flatus | Low risk | Some concerns | Low risk | Some concerns | Some concerns | Some concerns |
| Cai et al. (2025) | Length of hospital stay | Low risk | Some concerns | Low risk | Some concerns | Some concerns | Some concerns |
| Ding and Huang (2021) | Time to first flatus | Some concerns | Low risk | Low risk | Low risk | Some concerns | Some concerns |
| Ding and Huang (2021) | Time to first defecation | Some concerns | Low risk | Low risk | Low risk | Some concerns | Some concerns |
| Ding and Huang (2021) | Length of hospital stay | Some concerns | Low risk | Low risk | Low risk | Some concerns | Some concerns |
| Wang et al. (2026) | Time to first flatus | Some concerns | Low risk | Low risk | High risk | Some concerns | High risk |
| Wang et al. (2026) | Time to first defecation | Some concerns | Low risk | Low risk | High risk | Some concerns | High risk |
| Huang and Li (2023) | Time to first flatus | Some concerns | Low risk | Low risk | High risk | Some concerns | High risk |
| Huang and Li (2023) | Length of hospital stay | Some concerns | Low risk | Low risk | Some concerns | Some concerns | Some concerns |
| Shi et al. (2024) | Time to first flatus | Some concerns | Low risk | Low risk | High risk | Some concerns | High risk |
| Shi et al. (2024) | Time to first defecation | Some concerns | Low risk | Low risk | High risk | Some concerns | High risk |

Panel B. Results included in the narrative synthesis only (n = 5)

| **Study (comparison)** | **Outcome** | **D1** | **D2** | **D3** | **D4** | **D5** | **Overall** |
| --- | --- | --- | --- | --- | --- | --- | --- |
| Guo et al. (2015) (TEAS vs sham) | Time to first flatus | Some concerns | Some concerns | Some concerns | Some concerns | Some concerns | Some concerns |
| He et al. (2020) (FTS+LEAS vs FTS) | Time to first flatus | Some concerns | Some concerns | Some concerns | High risk | Some concerns | High risk |
| He et al. (2020) (FTS+LEAS vs FTS) | Time to first defecation | Some concerns | Some concerns | Some concerns | High risk | Some concerns | High risk |
| He et al. (2020) (LEAS vs conventional) | Time to first flatus | Some concerns | Some concerns | Some concerns | High risk | Some concerns | High risk |
| He et al. (2020) (LEAS vs conventional) | Time to first defecation | Some concerns | Some concerns | Some concerns | High risk | Some concerns | High risk |

Abbreviations: D1–D5, risk-of-bias domains 1 to 5 (randomization process; deviations from intended interventions; missing outcome data; measurement of the outcome; selection of the reported result).

The effect of assignment to intervention was the effect of interest throughout. Signaling-question responses, supporting evidence, and judgment rationales are provided in Supplementary Table S4.
