# Supplementary material for: Electrical acupoint stimulation for gastrointestinal recovery after gastrectomy for gastric cancer: a systematic review and meta-analysis
Source: Front Surg. 2026 Sep 16;13:1915755. doi: 10.3389/fsurg.2026.1915755 (PMC13625762; doi:10.3389/fsurg.2026.1915755)
Supplement: Supplementary file 5 [file Table4.docx]

**Supplementary Table S4. Full outcome-specific RoB 2 signaling-question responses, supporting evidence, and judgment rationales.**

Assessment framework: revised Cochrane Risk of Bias 2 tool for individually randomized parallel-group trials (22 August 2019), effect of assignment to intervention. Responses: Y, yes; PY, probably yes; PN, probably no; N, no; NI, no information; NA, not applicable. A separate assessment block is presented for every study-outcome result contributing to the four quantitative outcomes, except where two results from the same trial were assessed identically on every signaling question, in which case a single block covers both and the heading names both outcomes. The last two trials in this table contributed to the narrative synthesis only; for the four-arm trial by He et al., the two comparisons described in the Methods were assessed separately.

Domain 2 was judged from actual trial-context deviations, imbalanced co-interventions, post-randomization exclusions, and the analysis used to estimate the assignment effect. Lack of blinding alone was not used to raise the Domain 2 judgment; its potential influence on participant-reported or clinician-dependent outcomes was assessed separately in Domain 4.

Overall judgments followed the RoB 2 algorithm. When several domains raised some concerns, the combined implications were considered for each numerical result; no blanket rule was used either to retain “some concerns” or to escalate to “high risk.” No algorithm-proposed domain judgment was overridden. Statements that information was not reported are source-evidence statements rather than verbatim quotations.

Color coding: green, low risk; yellow, some concerns; red, high risk. Judgments are also presented as text so that interpretation does not depend on color alone.

Signaling questions (revised Cochrane RoB 2 tool for individually randomized parallel-group trials, effect of assignment to intervention). The question numbers used in the assessment blocks below refer to this list; the full wording is given here once and is not repeated in each block.

1.1 Was the allocation sequence random?

1.2 Was the allocation sequence concealed until participants were enrolled and assigned to interventions?

1.3 Did baseline differences between intervention groups suggest a problem with the randomization process?

2.1 Were participants aware of their assigned intervention during the trial?

2.2 Were carers and people delivering the interventions aware of participants’ assigned intervention during the trial?

2.3 If Y/PY/NI to 2.1 or 2.2: Were there deviations from the intended intervention that arose because of the trial context?

2.4 If Y/PY to 2.3: Were these deviations likely to have affected the outcome?

2.5 If Y/PY/NI to 2.4: Were these deviations from intended intervention balanced between groups?

2.6 Was an appropriate analysis used to estimate the effect of assignment to intervention?

2.7 If N/PN/NI to 2.6: Was there potential for a substantial impact (on the result) of the failure to analyse participants in the group to which they were randomized?

3.1 Were data for this outcome available for all, or nearly all, participants randomized?

3.2 If N/PN/NI to 3.1: Is there evidence that the result was not biased by missing outcome data?

3.3 If N/PN to 3.2: Could missingness in the outcome depend on its true value?

3.4 If Y/PY/NI to 3.3: Is it likely that missingness in the outcome depended on its true value?

4.1 Was the method of measuring the outcome inappropriate?

4.2 Could measurement or ascertainment of the outcome have differed between intervention groups?

4.3 If N/PN/NI to 4.1 and 4.2: Were outcome assessors aware of the intervention received by study participants?

4.4 If Y/PY/NI to 4.3: Could assessment of the outcome have been influenced by knowledge of intervention received?

4.5 If Y/PY/NI to 4.4: Is it likely that assessment of the outcome was influenced by knowledge of intervention received?

5.1 Were the data that produced this result analysed in accordance with a pre-specified analysis plan that was finalized before unblinded outcome data were available for analysis?

5.2 Is the numerical result being assessed likely to have been selected, on the basis of the results, from multiple eligible outcome measurements (e.g. scales, definitions, time points) within the outcome domain?

5.3 Is the numerical result being assessed likely to have been selected, on the basis of the results, from multiple eligible analyses of the data?

**Zheng et al. (2007)**

**Outcome: Time to first flatus**

*Assessment target: the full-sample time-to-first-flatus result (TEAS n=20 vs control n=20). The report labels the outcome as recovery of intestinal peristalsis but defines it as the interval from the end of surgery to the first passage of flatus.*

| **Domain / SQ** | **Response** | **Supporting quotation / source evidence** | **Location** | **Rationale** |
| --- | --- | --- | --- | --- |
| 1.1 | **NI** | The report states that 40 patients were randomly divided into two groups of 20; no method of sequence generation is described. | Methods, 病例选择及分组 | Randomization is asserted, but the sequence-generation method cannot be verified. |
| 1.2 | **NI** | No allocation-concealment procedure is described. | Methods | Concealment before assignment could not be verified. |
| 1.3 | **PN** | Table 1 reports sex ratio, age, body weight, duration of surgery, intraoperative fluid volume, and intraoperative blood loss; the report states that there were no statistically significant between-group differences in these variables. | Results, p. 398, Table 1; Abstract, Results | Baseline comparability is documented across all six reported variables. |
| **D1 judgment: Some concerns. Sequence generation and allocation concealment were insufficiently reported.** | | | | |
| 2.1 | **Y** | The control group received no stimulation; no sham device was applied. | Methods, TEAS方案 | Participants could distinguish whether stimulation was delivered during awake pre- and postoperative sessions. |
| 2.2 | **Y** | Stimulation was delivered openly by the trial team. | Methods | Intervention providers necessarily knew the assignment. |
| 2.3 | **PN** | Both groups received the same anesthetic regimen and the same patient-controlled epidural analgesia; no assignment-related deviations are reported. | Methods, 麻醉方法 and 术后镇痛 | Awareness of assignment was not accompanied by documented deviations or imbalanced co-interventions. |
| 2.6 | **Y** | All 20 randomized participants in each group were included in the reported analyses. | Results, Tables 1–3 | The reported analysis corresponds to the randomized groups. |
| **D2 judgment: Low risk. Although the trial was open-label, no trial-context deviations likely to affect the outcome were identified and all randomized participants were analyzed.** | | | | |
| 3.1 | **Y** | Twenty participants per group were randomized and analyzed; no withdrawals are reported. | Methods and Results | No missing outcome data were identified. |
| **D3 judgment: Low risk. Outcome data were available for all randomized participants.** | | | | |
| 4.1 | **N** | The outcome was defined as the interval from the end of surgery to the first passage of flatus. | Methods, 测定指标 | The outcome definition is clinically recognizable and appropriate for this review. |
| 4.2 | **N** | A single definition of time to first flatus—the interval from the end of surgery to the first passage of flatus—was applied to both groups; no group-specific measurement or ascertainment procedure is reported. | Methods, 测定指标; Results | The outcome definition and reported metric were the same in both groups, with no indication of differential ascertainment. |
| 4.3 | **Y** | No sham was used. First flatus is a participant/proxy-reported recovery milestone, and no blinded ascertainment procedure is described. | Methods | The person reporting the event necessarily had knowledge of whether stimulation was being delivered. |
| 4.4 | **Y** | The outcome depends on recognition and reporting of passage of flatus. | Methods | A report-dependent timing outcome can be influenced by expectations or differential reporting. |
| 4.5 | **PY** | The comparison was open-label and the report provides no procedure intended to shield the timing report from treatment knowledge. | Methods | Consistent with the approach used for other open-label, report-dependent recovery outcomes in this review. |
| **D4 judgment: High risk. The primary recovery milestone was assessed without masking in an open-label comparison.** | | | | |
| 5.1 | **NI** | No trial registration number, protocol, or statistical analysis plan is cited. | Whole report | A prespecified analysis plan could not be verified. |
| 5.2 | **PN** | A single bowel-recovery timing outcome is defined and a single result is reported. | Methods and Results | No evidence of selection among multiple eligible measurements was identified. |
| 5.3 | **PN** | No alternative eligible analysis of the first-flatus result is described. | Methods and Results | No evidence of selection among multiple analyses was identified. |
| **D5 judgment: Some concerns. Prospective prespecification could not be verified.** | | | | |
| **Overall risk-of-bias judgment: High risk. Driven by Domain 4.** | | | | |

**Chen et al. (2018)**

**Outcome: Time to first flatus**

*Assessment target: the full-sample time-to-first-flatus result (TEA n=33 vs no-treatment control n=30). The report contained an unresolved inconsistency regarding a wrist-bruising withdrawal.*

| **Domain / SQ** | **Response** | **Supporting quotation / source evidence** | **Location** | **Rationale** |
| --- | --- | --- | --- | --- |
| 1.1 | **Y** | “The remaining 63 patients were randomly allocated by computer algorithm to the TEA (n = 33) or control (n = 30) group.” | Chen et al., p. 15; Procedures and TEA | A computer algorithm was used to generate assignment. |
| 1.2 | **NI** | No allocation-concealment procedure was reported in the article. | Chen et al., Methods, pp. 14-16 | The randomization method was stated, but concealment before assignment could not be verified. |
| 1.3 | **PN** | Table 1 reported identical TEA-group counts for the pT and pN categories (1/7/14/8/3), and the control-group pN distribution (11/5/4/10/0) was presented without a P value. | Chen et al., p. 16; Table 1 and Results | Because the TEA-group pN row duplicated the TEA-group pT row exactly, it most probably reflects a transcription error; the apparent nodal-stage difference could therefore not be evaluated and was not treated as evidence that the randomization process had failed. Confidence in the reported baseline assessment was nevertheless reduced. |
| **D1 judgment: Some concerns** | | | | |
| 2.1 | **Y** | “The control group received no treatment…” and “it was not a double-blind research.” | Chen et al., pp. 15 and 18 | Participants could distinguish active TEA from no treatment. |
| 2.2 | **Y** | “The control group received no treatment…” | Chen et al., p. 15; Procedures and TEA | Personnel delivering or managing TEA necessarily knew the assigned group. |
| 2.3 | **Y** | “An adverse event of bruising on the wrist due to TEA was reported and the patient withdrew from the study.” | Chen et al., p. 16; Results | A discontinuation directly attributable to the experimental intervention was reported and therefore constituted a trial-context deviation. |
| 2.4 | **PN** | The reported outcome tables retained the randomized denominators (33 vs 30), and the timing and analytic handling of the withdrawal were not clearly described. | Chen et al., pp. 15-17; Figure 1 and Table 2 | The deviation could have affected treatment exposure, but one event was unlikely to materially alter the assessed recovery result. |
| 2.5 | **NA** | Not applicable based on the preceding signaling-question response. | - | The question was not reached in the RoB 2 pathway. |
| 2.6 | **PY** | The main outcome tables retained n=33 and n=30, matching the randomized groups. | Chen et al., pp. 15-17; Figure 1 and Table 2 | The numerical results appear to preserve participants in their assigned groups, although an explicit intention-to-treat statement and the handling of the wrist-bruising withdrawal were not provided. |
| 2.7 | **NA** | Not applicable based on the preceding signaling-question response. | - | The question was not reached in the RoB 2 pathway. |
| **D2 judgment: Low risk** | | | | |
| 3.1 | **PY** | Table 2 reports n=33 and n=30 for the randomized groups, despite a separate statement that one participant withdrew after wrist bruising. | Chen et al., pp. 15-17; Figure 1, Table 2 and Results | Data appear available for all or nearly all randomized participants, but the internal inconsistency prevents an unequivocal “Yes.” |
| 3.2 | **NA** | Not applicable based on the preceding signaling-question response. | - | The question was not reached in the RoB 2 pathway. |
| 3.3 | **NA** | Not applicable based on the preceding signaling-question response. | - | The question was not reached in the RoB 2 pathway. |
| 3.4 | **NA** | Not applicable based on the preceding signaling-question response. | - | The question was not reached in the RoB 2 pathway. |
| **D3 judgment: Low risk** | | | | |
| 4.1 | **N** | “The main outcomes were hours to the first flatus…” | Chen et al., p. 15; Procedures and TEA | Time to first flatus is a conventional postoperative recovery measure. |
| 4.2 | **N** | The same outcome definition was applied to both randomized groups. | Chen et al., Methods and Table 2, pp. 15-17 | No differential measurement method was described. |
| 4.3 | **Y** | “It was not a double-blind research, thus we could not exclude the placebo effect.” | Chen et al., p. 18; Limitations | Participants and clinical personnel were aware of allocation in a no-treatment-controlled trial. |
| 4.4 | **Y** | The result was time to first flatus in an unblinded trial. | Chen et al., pp. 15-18 | Reporting/ascertainment of a subjective, participant-dependent event could be influenced by treatment knowledge. |
| 4.5 | **PY** | “It was not a double-blind research, thus we could not exclude the placebo effect.” | Chen et al., p. 18; Limitations | The combination of no-treatment control, unblinded participants, and subjective event timing makes influence likely. |
| **D4 judgment: High risk** | | | | |
| 5.1 | **NI** | No prospective registration, protocol, or statistical analysis plan was reported in the article. | Chen et al., article report | Pre-specification before access to unblinded data could not be verified. |
| 5.2 | **PN** | “The main outcomes were hours to the first flatus…” | Chen et al., p. 15; Procedures and TEA | A single clinically defined measure was reported for the outcome domain, with no evidence of selection among multiple measures. |
| 5.3 | **PN** | “Statistical analysis was performed using SPSS version 24.0, using Student’s t test and χ² test…” | Chen et al., p. 16; Statistical analysis | Only a straightforward between-group analysis was described; selection from multiple eligible analyses was not evident. |
| **D5 judgment: Some concerns** | | | | |
| **Overall risk-of-bias judgment: High risk. High risk was driven by likely measurement bias for a participant-reported event in an unblinded, no-treatment-controlled trial.** | | | | |

**Chen et al. (2018)**

**Outcome: Length of hospital stay**

*Assessment target: the full-sample LOS result (8.06±1.75 vs 9.40±3.09 days), which was used for the assignment-effect synthesis. The complication-excluded modified result (7.73±1.14 vs 8.59±1.67 days) was not used.*

| **Domain / SQ** | **Response** | **Supporting quotation / source evidence** | **Location** | **Rationale** |
| --- | --- | --- | --- | --- |
| 1.1 | **Y** | “The remaining 63 patients were randomly allocated by computer algorithm to the TEA (n = 33) or control (n = 30) group.” | Chen et al., p. 15; Procedures and TEA | A computer algorithm was used to generate assignment. |
| 1.2 | **NI** | No allocation-concealment procedure was reported in the article. | Chen et al., Methods, pp. 14-16 | The randomization method was stated, but concealment before assignment could not be verified. |
| 1.3 | **PN** | Table 1 reported identical TEA-group counts for the pT and pN categories (1/7/14/8/3), and the control-group pN distribution (11/5/4/10/0) was presented without a P value. | Chen et al., p. 16; Table 1 and Results | Because the TEA-group pN row duplicated the TEA-group pT row exactly, it most probably reflects a transcription error; the apparent nodal-stage difference could therefore not be evaluated and was not treated as evidence that the randomization process had failed. Confidence in the reported baseline assessment was nevertheless reduced. |
| **D1 judgment: Some concerns** | | | | |
| 2.1 | **Y** | “The control group received no treatment…” and “it was not a double-blind research.” | Chen et al., pp. 15 and 18 | Participants could distinguish active TEA from no treatment. |
| 2.2 | **Y** | “The control group received no treatment…” | Chen et al., p. 15; Procedures and TEA | Personnel delivering or managing TEA necessarily knew the assigned group. |
| 2.3 | **Y** | “An adverse event of bruising on the wrist due to TEA was reported and the patient withdrew from the study.” | Chen et al., p. 16; Results | A discontinuation directly attributable to the experimental intervention was reported and therefore constituted a trial-context deviation. |
| 2.4 | **PN** | The reported outcome tables retained the randomized denominators (33 vs 30), and the timing and analytic handling of the withdrawal were not clearly described. | Chen et al., pp. 15-17; Figure 1 and Table 2 | The deviation could have affected treatment exposure, but one event was unlikely to materially alter the assessed recovery result. |
| 2.5 | **NA** | Not applicable based on the preceding signaling-question response. | - | The question was not reached in the RoB 2 pathway. |
| 2.6 | **PY** | The main outcome tables retained n=33 and n=30, matching the randomized groups. | Chen et al., pp. 15-17; Figure 1 and Table 2 | The numerical results appear to preserve participants in their assigned groups, although an explicit intention-to-treat statement and the handling of the wrist-bruising withdrawal were not provided. |
| 2.7 | **NA** | Not applicable based on the preceding signaling-question response. | - | The question was not reached in the RoB 2 pathway. |
| **D2 judgment: Low risk** | | | | |
| 3.1 | **PY** | Table 2 reports n=33 and n=30 for the randomized groups, despite a separate statement that one participant withdrew after wrist bruising. | Chen et al., pp. 15-17; Figure 1, Table 2 and Results | Data appear available for all or nearly all randomized participants, but the internal inconsistency prevents an unequivocal “Yes.” |
| 3.2 | **NA** | Not applicable based on the preceding signaling-question response. | - | The question was not reached in the RoB 2 pathway. |
| 3.3 | **NA** | Not applicable based on the preceding signaling-question response. | - | The question was not reached in the RoB 2 pathway. |
| 3.4 | **NA** | Not applicable based on the preceding signaling-question response. | - | The question was not reached in the RoB 2 pathway. |
| **D3 judgment: Low risk** | | | | |
| 4.1 | **N** | “Hospital stay (POD)” was reported as a postoperative outcome. | Chen et al., p. 17; Table 2 | Length of hospital stay measured in postoperative days is an appropriate outcome measure. |
| 4.2 | **N** | The same hospital-stay outcome and data source were used for both randomized groups. | Chen et al., Methods and Table 2, pp. 15-17 | No group-specific measurement method was reported. |
| 4.3 | **Y** | “The control group received no treatment…” and the authors stated that “it was not a double-blind research.” | Chen et al., pp. 15 and 18 | Participants and clinical personnel were aware of treatment allocation. |
| 4.4 | **Y** | No standardized discharge criteria or blinded discharge decision process was reported. | Chen et al., Methods, pp. 14-16 | Although the discharge date is objectively recorded, the timing of discharge could be influenced by clinicians’ knowledge of allocation. |
| 4.5 | **PN** | Hospital stay was recorded in postoperative days, and no evidence of differential discharge behavior was reported. | Chen et al., p. 17; Table 2 | Influence was possible, but the report did not provide evidence that allocation knowledge probably changed discharge timing. |
| **D4 judgment: Some concerns** | | | | |
| 5.1 | **NI** | No prospective registry record, protocol, or statistical analysis plan was identified in the published report. | Chen et al., article report | Pre-specification of the analysis before unblinded outcome data were available could not be verified. |
| 5.2 | **N** | Both the full-sample and complication-excluded LOS results were reported in Table 2. | Chen et al., pp. 15 and 17; Procedures and Table 2 | The full-sample estimate was used to represent the effect of assignment, rather than selecting between the reported results on the basis of statistical significance. |
| 5.3 | **PN** | “Statistical analysis was performed using SPSS version 24.0, using Student’s t test and χ² test.” | Chen et al., p. 16; Statistical analysis | A straightforward analysis was described; result-driven selection among multiple analyses was not evident. |
| **D5 judgment: Some concerns** | | | | |
| **Overall risk-of-bias judgment: Some concerns. Concerns arose from randomization reporting, unblinded clinician-dependent discharge timing, and absence of a prespecified analysis plan; these concerns were not considered sufficiently serious, individually or collectively, to warrant high risk.** | | | | |

**Gu et al. (2018)**

**Outcomes: Time to first flatus and time to first defecation**

*Assessment target: the long-duration TEAS versus control comparison (n=60 vs n=60) used in the meta-analysis. The short-duration arm delivered stimulation during surgery only and did not meet the eligibility criteria of this review. The reported units for first flatus and first defecation were printed as minutes but were interpreted as hours in the review because the values are physiologically compatible only with hours.*

| **Domain / SQ** | **Response** | **Supporting quotation / source evidence** | **Location** | **Rationale** |
| --- | --- | --- | --- | --- |
| 1.1 | **Y** | Participants were stratified into three age strata and allocated within each stratum using a random number table (随机数字表法). | Methods, 1.2 | An adequate random sequence-generation method is described. |
| 1.2 | **NI** | No allocation-concealment procedure is described. | Methods, 1.2 | Concealment before assignment could not be verified. |
| 1.3 | **PN** | Table 1 reports sex, age, body mass index, duration of surgery, tumor stage, extent of resection, and reconstruction with no significant differences; the reconstruction counts in the control group sum to 57 rather than 60. | Table 1 | The arithmetic inconsistency reduces confidence in reporting but does not itself demonstrate failed randomization. |
| **D1 judgment: Some concerns. Allocation concealment was not reported and one baseline row contains an internal counting inconsistency.** | | | | |
| 2.1 | **PY** | Electrodes were applied to the control group but the stimulator was not switched on. In the long-duration arm, stimulation began 1 h before induction and was repeated postoperatively every 8 h for 2 days while participants were awake. | Methods, 1.2 | Perceptible awake stimulation could allow participants to distinguish active treatment from the inactive device. |
| 2.2 | **Y** | The stimulator was operated openly. | Methods, 1.2 | Intervention providers knew the assignment. |
| 2.3 | **PN** | All groups received the same anesthetic regimen and the same postoperative patient-controlled intravenous analgesia; no assignment-related deviations are reported. | Methods, 1.2 | Co-interventions were specified and applied comparably across groups. |
| 2.6 | **Y** | All 180 randomized participants completed the trial and were analyzed in their assigned groups. | Results, 2.1 | The analysis corresponds to the randomized groups. |
| **D2 judgment: Low risk. Awareness of assignment was not accompanied by documented trial-context deviations, and all randomized participants were analyzed.** | | | | |
| 3.1 | **Y** | All 180 randomized participants completed the trial. | Results, 2.1 | No missing outcome data were reported. |
| **D3 judgment: Low risk. Outcome data were available for all randomized participants.** | | | | |
| 4.1 | **N** | Times to first flatus and first defecation were measured from the end of surgery, defined as completion of wound closure. | Methods, 1.3(4) | The outcome definitions are explicit and appropriate. |
| 4.2 | **N** | The same family-recorded procedure for first flatus and first defecation was specified for all three groups. | Methods, 1.3(4) | The measurement/ascertainment procedure was the same across groups. |
| 4.3 | **Y** | Times to first flatus and first defecation were recorded by participants’ family members (由家属记录). The active long-duration group received observable pre- and postoperative stimulation, whereas the control device was not activated. | Methods, 1.2 and 1.3(4) | The proxy reporters were not described as blinded and the intervention was readily distinguishable during awake sessions. |
| 4.4 | **Y** | Both outcomes depend on participant/proxy recognition and reporting of recovery events. | Methods, 1.3(4) | Report-dependent timing outcomes are susceptible to expectation and recording effects. |
| 4.5 | **PY** | Family members recorded the outcomes in an intervention comparison in which active postoperative stimulation was observable and control stimulation was inactive. | Methods, 1.2 and 1.3(4) | The combination of unblinded proxy recording and a distinguishable intervention makes influence on assessment plausible. |
| **D4 judgment: High risk. Unblinded proxy recording of participant-dependent recovery milestones.** | | | | |
| 5.1 | **NI** | No trial registration number or protocol is cited; ethics approval RMYY-YWLL-2016-01 is reported. | Methods, 1.1 | A prespecified analysis plan could not be verified. |
| 5.2 | **PN** | The prespecified gastrointestinal recovery measures are reported in Table 5. | Methods, 1.3 and Table 5 | No evidence of selective choice among multiple eligible measurements was identified. |
| 5.3 | **PN** | No alternative eligible analyses of these timing outcomes are described. | Methods and Results | No evidence of selective analysis was identified. |
| **D5 judgment: Some concerns. Prospective prespecification could not be verified.** | | | | |
| **Overall risk-of-bias judgment: High risk. Driven by Domain 4 for both outcomes.** | | | | |

**Gu et al. (2019)**

**Outcome: Time to first flatus**

*Assessment target: the reported time to first flatus result in the L-TEAS-versus-inactive-device comparison. Flatus and defecation were self-recorded by participants.*

| **Domain / SQ** | **Response** | **Supporting quotation / source evidence** | **Location** | **Rationale** |
| --- | --- | --- | --- | --- |
| 1.1 | **Y** | “Each enrolled patient was randomized according to a computer-generated random numbers table.” | Gu et al., p. 12; Patients | A computer-generated random-number table was used. |
| 1.2 | **NI** | No method for concealing the random sequence before enrollment and assignment was reported. | Gu et al., p. 12; Patients | Allocation concealment could not be verified. |
| 1.3 | **N** | “Baseline characteristics were not significantly different among the groups.” | Gu et al., p. 13; Results | No baseline pattern suggested failure of randomization. |
| **D1 judgment: Some concerns** | | | | |
| 2.1 | **PN** | “All patients were unaware of the group allocations.” | Gu et al., p. 12; Patients | Participants were probably masked by an inactive device with broken output wires, but the report did not assess whether masking was successful. |
| 2.2 | **Y** | “A single investigator was responsible for applying the type of intervention.” | Gu et al., p. 12; Patients | The intervention provider knew the allocated procedure, although data collectors and analysts were blinded. |
| 2.3 | **NI** | The flow diagram reports two intervention discontinuations in L-TEAS and one canceled surgery in C-TEAS, but does not explain whether these arose because of the trial context. | Gu et al., p. 14; Figure 2 | Post-randomization departures occurred, but their relationship to the trial context could not be determined. |
| 2.4 | **NA** | Not applicable based on the preceding signaling-question response. | - | The question was not reached in the RoB 2 pathway. |
| 2.5 | **NA** | Not applicable based on the preceding signaling-question response. | - | The question was not reached in the RoB 2 pathway. |
| 2.6 | **PN** | The flow diagram records one canceled surgery in the C-TEAS arm and two intervention discontinuations in the L-TEAS arm, and states that no participant was excluded from analysis, but it does not explain why the analyzed sample fell from 120 to 117. | Gu et al., p. 14; Figure 2 | It could not be established whether these three participants were absent because outcome data were unavailable, which would correspond to an acceptable modified intention-to-treat analysis, or because they did not receive or continue the assigned intervention, which would not. The remaining participants appear to have been analyzed in their randomized groups. The magnitude of the departure was considered at 2.7 and the missing data at Domain 3. |
| 2.7 | **PN** | Three of 120 randomized participants (one C-TEAS and two L-TEAS) were not included in the analyzed sample of 117. | Gu et al., p. 14; Figure 2 | The departure involved 2.5% of randomized participants and was approximately balanced between groups, so a substantial impact on the assessed result was unlikely. |
| **D2 judgment: Some concerns** | | | | |
| 3.1 | **Y** | “A total of 117 patients completed the trial” after 120 were randomized. | Gu et al., pp. 13-14; Results and Figure 2 | Outcome data were available for 97.5% of randomized participants, considered nearly all. |
| 3.2 | **NA** | Not applicable based on the preceding signaling-question response. | - | The question was not reached in the RoB 2 pathway. |
| 3.3 | **NA** | Not applicable based on the preceding signaling-question response. | - | The question was not reached in the RoB 2 pathway. |
| 3.4 | **NA** | Not applicable based on the preceding signaling-question response. | - | The question was not reached in the RoB 2 pathway. |
| **D3 judgment: Low risk** | | | | |
| 4.1 | **N** | “The return of gastrointestinal function was assessed using 3 parameters: time to first bowel sounds, flatus and defecation.” | Gu et al., p. 13; Measurements | The selected measures were appropriate conventional postoperative recovery outcomes. |
| 4.2 | **N** | “The patients were instructed to self-record the first postoperative flatus and defecation.” | Gu et al., p. 13; Measurements | The same self-recording procedure was used in both groups. |
| 4.3 | **NI** | Active TEAS produced de qi sensations at 5-30 mA, whereas control wires were broken; the authors stated, “we did not ask patients which group they thought they were in.” | Gu et al., pp. 12 and 16; Intervention and Limitations | Participants self-recorded flatus and defecation. Claimed masking was not formally verified despite a potentially perceptible sensory contrast. |
| 4.4 | **Y** | “The patients were instructed to self-record the first postoperative flatus and defecation.” | Gu et al., p. 13; Measurements | If participants inferred their assignment, knowledge could influence reporting of these subjective time-to-event outcomes. |
| 4.5 | **PN** | The article reported that patients were unaware of allocation, but did not test blinding success. | Gu et al., pp. 12 and 16 | Influence was possible, but there was insufficient evidence that participants probably knew assignment or that reporting was actually altered. |
| **D4 judgment: Some concerns** | | | | |
| 5.1 | **NI** | No prospective registry entry, protocol, or statistical analysis plan was reported in the article. | Gu et al., article report | Pre-specification before unblinded data were available could not be verified. |
| 5.2 | **PN** | Flatus and defecation were prespecified as distinct recovery parameters in the Methods. | Gu et al., p. 13; Measurements | There was no evidence that the reported numerical results were selected from multiple definitions or time points within either outcome domain. |
| 5.3 | **PN** | “Differences between the 2 groups were analyzed by Student’s t-test…” | Gu et al., p. 13; Data analysis | A single straightforward comparison was described for these continuous outcomes. |
| **D5 judgment: Some concerns** | | | | |
| **Overall risk-of-bias judgment: Some concerns. Several domains raised concerns, mainly because of incomplete concealment/reporting, unexplained post-randomization losses, uncertain success of participant masking, and lack of a prespecified analysis plan. No domain provided evidence of bias sufficiently serious to warrant high risk.** | | | | |

**Gu et al. (2019)**

**Outcome: Time to first defecation**

*Assessment target: the reported time to first defecation result in the L-TEAS-versus-inactive-device comparison. Flatus and defecation were self-recorded by participants.*

| **Domain / SQ** | **Response** | **Supporting quotation / source evidence** | **Location** | **Rationale** |
| --- | --- | --- | --- | --- |
| 1.1 | **Y** | “Each enrolled patient was randomized according to a computer-generated random numbers table.” | Gu et al., p. 12; Patients | A computer-generated random-number table was used. |
| 1.2 | **NI** | No method for concealing the random sequence before enrollment and assignment was reported. | Gu et al., p. 12; Patients | Allocation concealment could not be verified. |
| 1.3 | **N** | “Baseline characteristics were not significantly different among the groups.” | Gu et al., p. 13; Results | No baseline pattern suggested failure of randomization. |
| **D1 judgment: Some concerns** | | | | |
| 2.1 | **PN** | “All patients were unaware of the group allocations.” | Gu et al., p. 12; Patients | Participants were probably masked by an inactive device with broken output wires, but the report did not assess whether masking was successful. |
| 2.2 | **Y** | “A single investigator was responsible for applying the type of intervention.” | Gu et al., p. 12; Patients | The intervention provider knew the allocated procedure, although data collectors and analysts were blinded. |
| 2.3 | **NI** | The flow diagram reports two intervention discontinuations in L-TEAS and one canceled surgery in C-TEAS, but does not explain whether these arose because of the trial context. | Gu et al., p. 14; Figure 2 | Post-randomization departures occurred, but their relationship to the trial context could not be determined. |
| 2.4 | **NA** | Not applicable based on the preceding signaling-question response. | - | The question was not reached in the RoB 2 pathway. |
| 2.5 | **NA** | Not applicable based on the preceding signaling-question response. | - | The question was not reached in the RoB 2 pathway. |
| 2.6 | **PN** | The flow diagram records one canceled surgery in the C-TEAS arm and two intervention discontinuations in the L-TEAS arm, and states that no participant was excluded from analysis, but it does not explain why the analyzed sample fell from 120 to 117. | Gu et al., p. 14; Figure 2 | It could not be established whether these three participants were absent because outcome data were unavailable, which would correspond to an acceptable modified intention-to-treat analysis, or because they did not receive or continue the assigned intervention, which would not. The remaining participants appear to have been analyzed in their randomized groups. The magnitude of the departure was considered at 2.7 and the missing data at Domain 3. |
| 2.7 | **PN** | Three of 120 randomized participants (one C-TEAS and two L-TEAS) were not included in the analyzed sample of 117. | Gu et al., p. 14; Figure 2 | The departure involved 2.5% of randomized participants and was approximately balanced between groups, so a substantial impact on the assessed result was unlikely. |
| **D2 judgment: Some concerns** | | | | |
| 3.1 | **Y** | “A total of 117 patients completed the trial” after 120 were randomized. | Gu et al., pp. 13-14; Results and Figure 2 | Outcome data were available for 97.5% of randomized participants, considered nearly all. |
| 3.2 | **NA** | Not applicable based on the preceding signaling-question response. | - | The question was not reached in the RoB 2 pathway. |
| 3.3 | **NA** | Not applicable based on the preceding signaling-question response. | - | The question was not reached in the RoB 2 pathway. |
| 3.4 | **NA** | Not applicable based on the preceding signaling-question response. | - | The question was not reached in the RoB 2 pathway. |
| **D3 judgment: Low risk** | | | | |
| 4.1 | **N** | “The return of gastrointestinal function was assessed using 3 parameters: time to first bowel sounds, flatus and defecation.” | Gu et al., p. 13; Measurements | The selected measures were appropriate conventional postoperative recovery outcomes. |
| 4.2 | **N** | “The patients were instructed to self-record the first postoperative flatus and defecation.” | Gu et al., p. 13; Measurements | The same self-recording procedure was used in both groups. |
| 4.3 | **NI** | Active TEAS produced de qi sensations at 5-30 mA, whereas control wires were broken; the authors stated, “we did not ask patients which group they thought they were in.” | Gu et al., pp. 12 and 16; Intervention and Limitations | Participants self-recorded flatus and defecation. Claimed masking was not formally verified despite a potentially perceptible sensory contrast. |
| 4.4 | **Y** | “The patients were instructed to self-record the first postoperative flatus and defecation.” | Gu et al., p. 13; Measurements | If participants inferred their assignment, knowledge could influence reporting of these subjective time-to-event outcomes. |
| 4.5 | **PN** | The article reported that patients were unaware of allocation, but did not test blinding success. | Gu et al., pp. 12 and 16 | Influence was possible, but there was insufficient evidence that participants probably knew assignment or that reporting was actually altered. |
| **D4 judgment: Some concerns** | | | | |
| 5.1 | **NI** | No prospective registry entry, protocol, or statistical analysis plan was reported in the article. | Gu et al., article report | Pre-specification before unblinded data were available could not be verified. |
| 5.2 | **PN** | Flatus and defecation were prespecified as distinct recovery parameters in the Methods. | Gu et al., p. 13; Measurements | There was no evidence that the reported numerical results were selected from multiple definitions or time points within either outcome domain. |
| 5.3 | **PN** | “Differences between the 2 groups were analyzed by Student’s t-test…” | Gu et al., p. 13; Data analysis | A single straightforward comparison was described for these continuous outcomes. |
| **D5 judgment: Some concerns** | | | | |
| **Overall risk-of-bias judgment: Some concerns. Several domains raised concerns, mainly because of incomplete concealment/reporting, unexplained post-randomization losses, uncertain success of participant masking, and lack of a prespecified analysis plan. No domain provided evidence of bias sufficiently serious to warrant high risk.** | | | | |

**Zhou et al. (2021)**

**Outcome: Time to first flatus**

*Assessment target: the time to first flatus result in the unblinded TEAS-versus-usual-care comparison. One randomized control participant was excluded under a postoperative-complication withdrawal rule.*

| **Domain / SQ** | **Response** | **Supporting quotation / source evidence** | **Location** | **Rationale** |
| --- | --- | --- | --- | --- |
| 1.1 | **Y** | “We randomized patients according to the order of the random number table generated by SPSS 21.0.” | Zhou et al., p. 1452; Randomization | A computer-generated random-number table was used. |
| 1.2 | **NI** | “Yuqi Sun was responsible for allocating the patients according to the random number table.” | Zhou et al., p. 1452; Randomization | The report did not state that the sequence was concealed from the allocator until enrollment and assignment. |
| 1.3 | **N** | “There were no significant differences between 2 groups (P > 0.05).” | Zhou et al., p. 1453; Baseline Data | Baseline data did not suggest a compromised randomization process. |
| **D1 judgment: Some concerns** | | | | |
| 2.1 | **Y** | “This was an unblinded randomized controlled trial.” | Zhou et al., p. 1450; Trial Design | Participants were aware of allocation. |
| 2.2 | **Y** | “This was an unblinded randomized controlled trial.” | Zhou et al., p. 1450; Trial Design | Clinical staff and intervention providers were not blinded. |
| 2.3 | **PN** | “During the perioperative period, the same ERAS protocol … was performed on both groups.” | Zhou et al., p. 1450; Trial Design | No important imbalance in intended co-interventions or other trial-context deviation was reported. |
| 2.4 | **NA** | Not applicable based on the preceding signaling-question response. | - | The question was not reached in the RoB 2 pathway. |
| 2.5 | **NA** | Not applicable based on the preceding signaling-question response. | - | The question was not reached in the RoB 2 pathway. |
| 2.6 | **N** | The protocol stated that patients with Clavien-Dindo grade >2 met withdrawal criteria; Figure 1 shows 82 randomized and 81 analyzed after one control participant with pulmonary infection withdrew. | Zhou et al., pp. 1450 and 1452; Withdrawal criteria and Figure 1 | The analysis excluded a randomized participant according to a post-randomization clinical event and therefore did not strictly estimate the effect of assignment. |
| 2.7 | **PN** | Only 1 of 82 randomized participants was excluded, although the exclusion was linked to a postoperative complication. | Zhou et al., pp. 1450 and 1452 | The outcome-dependent exclusion created concern, but a substantial effect on the numerical estimate was unlikely given its small magnitude. |
| **D2 judgment: Some concerns** | | | | |
| 3.1 | **Y** | “Eighty-two patients… were randomly assigned” and 81 were included in the analysis. | Zhou et al., pp. 1450 and 1452 | Outcome data were available for 98.8% of randomized participants, considered nearly all. |
| 3.2 | **NA** | Not applicable based on the preceding signaling-question response. | - | The question was not reached in the RoB 2 pathway. |
| 3.3 | **NA** | Not applicable based on the preceding signaling-question response. | - | The question was not reached in the RoB 2 pathway. |
| 3.4 | **NA** | Not applicable based on the preceding signaling-question response. | - | The question was not reached in the RoB 2 pathway. |
| **D3 judgment: Low risk** | | | | |
| 4.1 | **N** | The secondary outcomes included “time of first postoperative flatus and defecation.” | Zhou et al., p. 1451; Outcomes | These are conventional postoperative recovery measures. |
| 4.2 | **N** | The same definitions and follow-up schedule were applied to both groups. | Zhou et al., Outcomes and Results, pp. 1451-1453 | No differential measurement method was described. |
| 4.3 | **Y** | “This was an unblinded randomized controlled trial” and “All data were recorded by Yulong Tian and Zequn Li.” | Zhou et al., pp. 1450-1451 | The report did not describe blinded ascertainment of these participant-dependent outcomes. |
| 4.4 | **Y** | The outcomes were the times of first flatus and defecation in an explicitly unblinded trial. | Zhou et al., pp. 1450-1451 | Knowledge of allocation could influence participant reporting or investigator ascertainment. |
| 4.5 | **PY** | The report did not use sham stimulation or blinded outcome ascertainment for these subjective recovery events. | Zhou et al., Trial Design and Outcomes, pp. 1450-1451 | Influence from allocation knowledge was considered likely for the reported time-to-event outcomes. |
| **D4 judgment: High risk** | | | | |
| 5.1 | **PY** | “This trial has been registered… ChiCTR1900022692. The date of trial registration was April 22, 2019” before recruitment began in May 2019; the full protocol was stated to be available on request. | Zhou et al., p. 1450; Trial Design | Prospective registration and protocol availability support pre-specification, although a separate SAP was not provided in the article. |
| 5.2 | **N** | The registered/published secondary outcomes explicitly included time to first postoperative flatus and defecation. | Zhou et al., pp. 1449 and 1451; Abstract and Outcomes | No evidence suggested selection from multiple eligible measurements within these domains. |
| 5.3 | **PN** | The Methods described Student’s t-test or Wilcoxon testing according to distribution. | Zhou et al., p. 1452; Statistical Methods | There was no indication that the reported result was selected from multiple analyses based on significance. |
| **D5 judgment: Low risk** | | | | |
| **Overall risk-of-bias judgment: High risk. High risk was driven by likely measurement bias for a participant-dependent recovery event in an explicitly unblinded trial.** | | | | |

**Zhou et al. (2021)**

**Outcome: Time to first defecation**

*Assessment target: the time to first defecation result in the unblinded TEAS-versus-usual-care comparison. One randomized control participant was excluded under a postoperative-complication withdrawal rule.*

| **Domain / SQ** | **Response** | **Supporting quotation / source evidence** | **Location** | **Rationale** |
| --- | --- | --- | --- | --- |
| 1.1 | **Y** | “We randomized patients according to the order of the random number table generated by SPSS 21.0.” | Zhou et al., p. 1452; Randomization | A computer-generated random-number table was used. |
| 1.2 | **NI** | “Yuqi Sun was responsible for allocating the patients according to the random number table.” | Zhou et al., p. 1452; Randomization | The report did not state that the sequence was concealed from the allocator until enrollment and assignment. |
| 1.3 | **N** | “There were no significant differences between 2 groups (P > 0.05).” | Zhou et al., p. 1453; Baseline Data | Baseline data did not suggest a compromised randomization process. |
| **D1 judgment: Some concerns** | | | | |
| 2.1 | **Y** | “This was an unblinded randomized controlled trial.” | Zhou et al., p. 1450; Trial Design | Participants were aware of allocation. |
| 2.2 | **Y** | “This was an unblinded randomized controlled trial.” | Zhou et al., p. 1450; Trial Design | Clinical staff and intervention providers were not blinded. |
| 2.3 | **PN** | “During the perioperative period, the same ERAS protocol … was performed on both groups.” | Zhou et al., p. 1450; Trial Design | No important imbalance in intended co-interventions or other trial-context deviation was reported. |
| 2.4 | **NA** | Not applicable based on the preceding signaling-question response. | - | The question was not reached in the RoB 2 pathway. |
| 2.5 | **NA** | Not applicable based on the preceding signaling-question response. | - | The question was not reached in the RoB 2 pathway. |
| 2.6 | **N** | The protocol stated that patients with Clavien-Dindo grade >2 met withdrawal criteria; Figure 1 shows 82 randomized and 81 analyzed after one control participant with pulmonary infection withdrew. | Zhou et al., pp. 1450 and 1452; Withdrawal criteria and Figure 1 | The analysis excluded a randomized participant according to a post-randomization clinical event and therefore did not strictly estimate the effect of assignment. |
| 2.7 | **PN** | Only 1 of 82 randomized participants was excluded, although the exclusion was linked to a postoperative complication. | Zhou et al., pp. 1450 and 1452 | The outcome-dependent exclusion created concern, but a substantial effect on the numerical estimate was unlikely given its small magnitude. |
| **D2 judgment: Some concerns** | | | | |
| 3.1 | **Y** | “Eighty-two patients… were randomly assigned” and 81 were included in the analysis. | Zhou et al., pp. 1450 and 1452 | Outcome data were available for 98.8% of randomized participants, considered nearly all. |
| 3.2 | **NA** | Not applicable based on the preceding signaling-question response. | - | The question was not reached in the RoB 2 pathway. |
| 3.3 | **NA** | Not applicable based on the preceding signaling-question response. | - | The question was not reached in the RoB 2 pathway. |
| 3.4 | **NA** | Not applicable based on the preceding signaling-question response. | - | The question was not reached in the RoB 2 pathway. |
| **D3 judgment: Low risk** | | | | |
| 4.1 | **N** | The secondary outcomes included “time of first postoperative flatus and defecation.” | Zhou et al., p. 1451; Outcomes | These are conventional postoperative recovery measures. |
| 4.2 | **N** | The same definitions and follow-up schedule were applied to both groups. | Zhou et al., Outcomes and Results, pp. 1451-1453 | No differential measurement method was described. |
| 4.3 | **Y** | “This was an unblinded randomized controlled trial” and “All data were recorded by Yulong Tian and Zequn Li.” | Zhou et al., pp. 1450-1451 | The report did not describe blinded ascertainment of these participant-dependent outcomes. |
| 4.4 | **Y** | The outcomes were the times of first flatus and defecation in an explicitly unblinded trial. | Zhou et al., pp. 1450-1451 | Knowledge of allocation could influence participant reporting or investigator ascertainment. |
| 4.5 | **PY** | The report did not use sham stimulation or blinded outcome ascertainment for these subjective recovery events. | Zhou et al., Trial Design and Outcomes, pp. 1450-1451 | Influence from allocation knowledge was considered likely for the reported time-to-event outcomes. |
| **D4 judgment: High risk** | | | | |
| 5.1 | **PY** | “This trial has been registered… ChiCTR1900022692. The date of trial registration was April 22, 2019” before recruitment began in May 2019; the full protocol was stated to be available on request. | Zhou et al., p. 1450; Trial Design | Prospective registration and protocol availability support pre-specification, although a separate SAP was not provided in the article. |
| 5.2 | **N** | The registered/published secondary outcomes explicitly included time to first postoperative flatus and defecation. | Zhou et al., pp. 1449 and 1451; Abstract and Outcomes | No evidence suggested selection from multiple eligible measurements within these domains. |
| 5.3 | **PN** | The Methods described Student’s t-test or Wilcoxon testing according to distribution. | Zhou et al., p. 1452; Statistical Methods | There was no indication that the reported result was selected from multiple analyses based on significance. |
| **D5 judgment: Low risk** | | | | |
| **Overall risk-of-bias judgment: High risk. High risk was driven by likely measurement bias for a participant-dependent recovery event in an explicitly unblinded trial.** | | | | |

**Zhou et al. (2021)**

**Outcome: Length of hospital stay**

*Assessment target: postoperative length of stay in the unblinded TEAS-versus-usual-care comparison. One control participant was excluded under a Clavien-Dindo grade* ***>****2 withdrawal criterion.*

| **Domain / SQ** | **Response** | **Supporting quotation / source evidence** | **Location** | **Rationale** |
| --- | --- | --- | --- | --- |
| 1.1 | **Y** | “We randomized patients according to the order of the random number table generated by SPSS 21.0.” | Zhou et al., p. 1452; Randomization | A computer-generated random-number table was used. |
| 1.2 | **NI** | “Yuqi Sun was responsible for allocating the patients according to the random number table.” | Zhou et al., p. 1452; Randomization | The report did not state that the sequence was concealed from the allocator until enrollment and assignment. |
| 1.3 | **N** | “There were no significant differences between 2 groups (P > 0.05).” | Zhou et al., p. 1453; Baseline Data | Baseline data did not suggest a compromised randomization process. |
| **D1 judgment: Some concerns** | | | | |
| 2.1 | **Y** | “This was an unblinded randomized controlled trial.” | Zhou et al., p. 1450; Trial Design | Participants were aware of allocation. |
| 2.2 | **Y** | “This was an unblinded randomized controlled trial.” | Zhou et al., p. 1450; Trial Design | Clinical staff and intervention providers were not blinded. |
| 2.3 | **PN** | “During the perioperative period, the same ERAS protocol … was performed on both groups.” | Zhou et al., p. 1450; Trial Design | No important imbalance in intended co-interventions or other trial-context deviation was reported. |
| 2.4 | **NA** | Not applicable based on the preceding signaling-question response. | - | The question was not reached in the RoB 2 pathway. |
| 2.5 | **NA** | Not applicable based on the preceding signaling-question response. | - | The question was not reached in the RoB 2 pathway. |
| 2.6 | **N** | The protocol stated that patients with Clavien-Dindo grade >2 met withdrawal criteria; Figure 1 shows 82 randomized and 81 analyzed after one control participant with pulmonary infection withdrew. | Zhou et al., pp. 1450 and 1452; Withdrawal criteria and Figure 1 | The analysis excluded a randomized participant according to a post-randomization clinical event and therefore did not strictly estimate the effect of assignment. |
| 2.7 | **PN** | Only 1 of 82 randomized participants was excluded, although the exclusion was linked to a postoperative complication. | Zhou et al., pp. 1450 and 1452 | The outcome-dependent exclusion created concern, but a substantial effect on the numerical estimate was unlikely given its small magnitude. |
| **D2 judgment: Some concerns** | | | | |
| 3.1 | **Y** | “Eighty-two patients… were randomly assigned” and 81 were included in the analysis. | Zhou et al., pp. 1450 and 1452 | Outcome data were available for 98.8% of randomized participants, considered nearly all. |
| 3.2 | **NA** | Not applicable based on the preceding signaling-question response. | - | The question was not reached in the RoB 2 pathway. |
| 3.3 | **NA** | Not applicable based on the preceding signaling-question response. | - | The question was not reached in the RoB 2 pathway. |
| 3.4 | **NA** | Not applicable based on the preceding signaling-question response. | - | The question was not reached in the RoB 2 pathway. |
| **D3 judgment: Low risk** | | | | |
| 4.1 | **N** | The prespecified secondary outcomes included “length of postoperative day.” | Zhou et al., p. 1451; Outcomes | Postoperative length of stay is an appropriate outcome measure. |
| 4.2 | **N** | The same ERAS pathway and outcome collection procedures were applied to both groups. | Zhou et al., pp. 1450-1451; Trial Design and Outcomes | No different ascertainment method between groups was described. |
| 4.3 | **Y** | “This was an unblinded randomized controlled trial.” | Zhou et al., p. 1450; Trial Design | Participants and clinical personnel were aware of allocation. |
| 4.4 | **Y** | No blinded discharge assessment or explicit standardized discharge criteria were reported. | Zhou et al., Methods, pp. 1450-1452 | The recorded discharge date is objective, but the clinical decision determining discharge could potentially be influenced by allocation knowledge. |
| 4.5 | **PN** | “During the perioperative period, the same ERAS protocol … was performed on both groups,” and no differential discharge practice was reported. | Zhou et al., p. 1450; Trial Design | Influence was possible but not shown to be likely under the common perioperative pathway. |
| **D4 judgment: Some concerns** | | | | |
| 5.1 | **PY** | “This trial has been registered … ChiCTR1900022692. The date of trial registration was April 22, 2019” before recruitment began; the full protocol was stated to be available on request. | Zhou et al., p. 1450; Trial Design | Prospective registration supports pre-specification, although a separate finalized SAP was not available in the article. |
| 5.2 | **N** | “Length of postoperative day” was explicitly listed among the secondary outcomes. | Zhou et al., p. 1451; Outcomes | The reported result corresponded to a prespecified outcome, with no indication of selection among multiple eligible measurements. |
| 5.3 | **PN** | The Methods prespecified Student’s t-test or Wilcoxon rank-sum testing according to the distribution of continuous variables. | Zhou et al., p. 1452; Statistical Methods | There was no indication that the LOS analysis was selected from multiple analyses on the basis of the result. |
| **D5 judgment: Low risk** | | | | |
| **Overall risk-of-bias judgment: Some concerns. Concerns arose from allocation concealment, post-randomization exclusion, and potential influence of unblinded discharge decisions. These concerns were not considered sufficiently serious, individually or collectively, to warrant high risk.** | | | | |

**Pei et al. (2025)**

**Outcome: Time to first flatus**

*Assessment target: time to first flatus in the EA-versus-sham-EA comparison used in the meta-analysis.*

| **Domain / SQ** | **Response** | **Supporting quotation / source evidence** | **Location** | **Rationale** |
| --- | --- | --- | --- | --- |
| 1.1 | **Y** | “Randomization was stratified by enrollment site with a block size of 9…” | Pei et al., p. 75; Randomization and Blinding | A stratified block-randomization method was used. |
| 1.2 | **Y** | “Randomization and allocation concealment were conducted using the Electronic Data Capture System…” | Pei et al., p. 75; Randomization and Blinding | Central electronic allocation provided concealment. |
| 1.3 | **N** | “Baseline characteristics were similar across the groups, except for family history of gastric cancer.” | Pei et al., p. 76; Participants | A single modest imbalance was compatible with chance and was adjusted for in the analyses; it did not suggest compromised randomization. |
| **D1 judgment: Low risk** | | | | |
| 2.1 | **PN** | Matched blunt placebo needles were used. Blinding was formally assessed at two of the seven centers: 75 of 105 EA participants (71.43%) and 68 of 100 sham-EA participants (68.00%) believed they had received true EA (P = 0.79). Participants’ baseline expectations of acupuncture were also similar between the two groups (P = 0.49 and P = 0.35 for the two expectation items). | Pei et al., pp. 75-76; Randomization and Blinding, and Supplementary Material 2, eTables 3 and 4 | Similar treatment guesses and similar baseline expectations support successful participant masking in the EA-versus-sham-EA comparison, although the formal assessment was limited to two of the seven centers. |
| 2.2 | **Y** | “Surgeons, outcome assessors, and statisticians were blinded,” while licensed acupuncturists delivered EA or SA. | Pei et al., p. 75; Randomization and Blinding/Interventions | Acupuncturists necessarily knew the procedure, although other clinical and analytical personnel were masked. |
| 2.3 | **PN** | “All participants received perioperative standard care…” and EA/SA procedures were standardized. Some participants discontinued sessions or missed the 2-hour visit. | Pei et al., pp. 75 and 77 | Incomplete sessions were reported, but no important deviation caused by the trial context or imbalanced co-intervention was shown to affect the outcomes. |
| 2.4 | **NA** | Not applicable based on the preceding signaling-question response. | - | The question was not reached in the RoB 2 pathway. |
| 2.5 | **NA** | Not applicable based on the preceding signaling-question response. | - | The question was not reached in the RoB 2 pathway. |
| 2.6 | **PN** | The report describes five post-randomization exclusions before the arm-specific randomized totals are presented: one participant who underwent secondary surgery because of a severe postoperative complication, one erroneously randomized twice, and three found to have noncancerous gastric pathology. The report states that 585 participants were randomized overall; after these five exclusions the flow diagram displays group sizes of 195 (EA), 190 (sham EA) and 195 (standard care). One further participant in each of the EA and sham-EA arms was excluded from the full analysis set because no intervention was received and no efficacy outcome was recorded (194/195 and 189/190 analyzed). | Pei et al., pp. 76-77; Results and Figure 1 | Exclusion of participants whose ineligibility was ascertained only after randomization, and could not have been influenced by group assignment, may be acceptable; this applies to the duplicate randomization record and the three noncancerous pathologies. However, the exclusion following secondary surgery was based on a post-randomization clinical event. The treatment allocation of the five excluded participants was not reported, so the arm-specific randomized denominators could not be reconstructed. It could therefore not be confirmed that all eligible randomized participants were analyzed in the groups to which they were assigned. |
| 2.7 | **PN** | Two participants, one in each of the EA and sham-EA arms, were excluded from the full analysis set because no efficacy outcome data were recorded. The treatment allocation of the five earlier post-randomization exclusions was not reported. | Pei et al., pp. 76-77; Results and Figure 1 | Depending on the allocation of the five earlier exclusions, between 2 and 7 randomized participants were absent from the EA-versus-sham-EA analysis, corresponding to approximately 0.5% to 1.8% of the participants randomized to the two relevant arms. The exclusions were small in number, and no direct evidence indicated that they substantially altered the treatment estimate. However, because the allocation of the five earlier exclusions was not reported and may have been concentrated in the comparator arm, a substantial impact on the result could not be entirely excluded. |
| **D2 judgment: Some concerns** | | | | |
| 3.1 | **Y** | “No missing data were reported for the primary outcome.” | Pei et al., p. 76; Statistical Analysis | Time to first flatus was available for all participants in the full-analysis set. |
| 3.2 | **NA** | Not applicable based on the preceding signaling-question response. | - | The question was not reached in the RoB 2 pathway. |
| 3.3 | **NA** | Not applicable based on the preceding signaling-question response. | - | The question was not reached in the RoB 2 pathway. |
| 3.4 | **NA** | Not applicable based on the preceding signaling-question response. | - | The question was not reached in the RoB 2 pathway. |
| **D3 judgment: Low risk** | | | | |
| 4.1 | **N** | The outcomes were time to first flatus, first defecation, and first water intake, measured in hours after surgery. | Pei et al., pp. 75 and 80; Outcome Assessments and Table 2 | These were appropriate prespecified postoperative recovery measures. |
| 4.2 | **N** | “The time-related outcomes were initially recorded by the patients themselves; subsequently, nurses on each specific 8-hour shift reminded them to report.” | Pei et al., p. 75; Outcome Assessments | The same ascertainment method was applied across EA and SA groups. |
| 4.3 | **PN** | “Surgeons, outcome assessors, and statisticians were blinded,” and matched sham procedures were used. | Pei et al., p. 75; Randomization and Blinding, and Supplementary Material 2, eTable 4 | Outcome assessors were blinded and participants were probably blinded, although blinding success was formally evaluated at only two of the seven centers. |
| 4.4 | **NA** | Not applicable based on the preceding signaling-question response. | - | The question was not reached in the RoB 2 pathway. |
| 4.5 | **NA** | Not applicable based on the preceding signaling-question response. | - | The question was not reached in the RoB 2 pathway. |
| **D4 judgment: Low risk** | | | | |
| 5.1 | **Y** | The trial was prospectively registered (ChiCTR2100050660), and “The study protocol and statistical analysis plan are available as part of Supplementary Material 1.” | Pei et al., pp. 74 and 84; Study Design/Data Availability | A prospective registry, protocol, and SAP were available before final analysis. |
| 5.2 | **N** | The protocol-defined outcomes included time to first flatus, first defecation, and water intake, and Table 2 reports these prespecified measures. | Pei et al., pp. 75 and 80 | No result-based selection among multiple measurements was evident. |
| 5.3 | **N** | “The primary outcome was assessed using analysis of covariance…” and “The same approach applied to most secondary outcomes,” with a prespecified fixed-sequence/Bonferroni strategy. | Pei et al., pp. 75-76; Statistical Analysis | The analysis methods were prospectively structured and did not appear selected based on results. |
| **D5 judgment: Low risk** | | | | |
| **Overall risk-of-bias judgment: Some concerns. Randomization was centrally concealed, participants and outcome assessors were masked, the report explicitly stated that no data were missing for this primary outcome, and a prospective protocol and statistical analysis plan were available. Concerns arose in Domain 2 because the treatment allocation and analytic handling of five post-randomization exclusions were not reported.** | | | | |

**Pei et al. (2025)**

**Outcome: Time to first defecation**

*Assessment target: time to first defecation in the EA-versus-sham-EA comparison used in the meta-analysis.*

| **Domain / SQ** | **Response** | **Supporting quotation / source evidence** | **Location** | **Rationale** |
| --- | --- | --- | --- | --- |
| 1.1 | **Y** | “Randomization was stratified by enrollment site with a block size of 9…” | Pei et al., p. 75; Randomization and Blinding | A stratified block-randomization method was used. |
| 1.2 | **Y** | “Randomization and allocation concealment were conducted using the Electronic Data Capture System…” | Pei et al., p. 75; Randomization and Blinding | Central electronic allocation provided concealment. |
| 1.3 | **N** | “Baseline characteristics were similar across the groups, except for family history of gastric cancer.” | Pei et al., p. 76; Participants | A single modest imbalance was compatible with chance and was adjusted for in the analyses; it did not suggest compromised randomization. |
| **D1 judgment: Low risk** | | | | |
| 2.1 | **PN** | Matched blunt placebo needles were used. Blinding was formally assessed at two of the seven centers: 75 of 105 EA participants (71.43%) and 68 of 100 sham-EA participants (68.00%) believed they had received true EA (P = 0.79). Participants’ baseline expectations of acupuncture were also similar between the two groups (P = 0.49 and P = 0.35 for the two expectation items). | Pei et al., pp. 75-76; Randomization and Blinding, and Supplementary Material 2, eTables 3 and 4 | Similar treatment guesses and similar baseline expectations support successful participant masking in the EA-versus-sham-EA comparison, although the formal assessment was limited to two of the seven centers. |
| 2.2 | **Y** | “Surgeons, outcome assessors, and statisticians were blinded,” while licensed acupuncturists delivered EA or SA. | Pei et al., p. 75; Randomization and Blinding/Interventions | Acupuncturists necessarily knew the procedure, although other clinical and analytical personnel were masked. |
| 2.3 | **PN** | “All participants received perioperative standard care…” and EA/SA procedures were standardized. Some participants discontinued sessions or missed the 2-hour visit. | Pei et al., pp. 75 and 77 | Incomplete sessions were reported, but no important deviation caused by the trial context or imbalanced co-intervention was shown to affect the outcomes. |
| 2.4 | **NA** | Not applicable based on the preceding signaling-question response. | - | The question was not reached in the RoB 2 pathway. |
| 2.5 | **NA** | Not applicable based on the preceding signaling-question response. | - | The question was not reached in the RoB 2 pathway. |
| 2.6 | **PN** | The report describes five post-randomization exclusions before the arm-specific randomized totals are presented: one participant who underwent secondary surgery because of a severe postoperative complication, one erroneously randomized twice, and three found to have noncancerous gastric pathology. The report states that 585 participants were randomized overall; after these five exclusions the flow diagram displays group sizes of 195 (EA), 190 (sham EA) and 195 (standard care). One further participant in each of the EA and sham-EA arms was excluded from the full analysis set because no intervention was received and no efficacy outcome was recorded (194/195 and 189/190 analyzed). | Pei et al., pp. 76-77; Results and Figure 1 | Exclusion of participants whose ineligibility was ascertained only after randomization, and could not have been influenced by group assignment, may be acceptable; this applies to the duplicate randomization record and the three noncancerous pathologies. However, the exclusion following secondary surgery was based on a post-randomization clinical event. The treatment allocation of the five excluded participants was not reported, so the arm-specific randomized denominators could not be reconstructed. It could therefore not be confirmed that all eligible randomized participants were analyzed in the groups to which they were assigned. |
| 2.7 | **PN** | Two participants, one in each of the EA and sham-EA arms, were excluded from the full analysis set because no efficacy outcome data were recorded. The treatment allocation of the five earlier post-randomization exclusions was not reported. | Pei et al., pp. 76-77; Results and Figure 1 | Depending on the allocation of the five earlier exclusions, between 2 and 7 randomized participants were absent from the EA-versus-sham-EA analysis, corresponding to approximately 0.5% to 1.8% of the participants randomized to the two relevant arms. The exclusions were small in number, and no direct evidence indicated that they substantially altered the treatment estimate. However, because the allocation of the five earlier exclusions was not reported and may have been concentrated in the comparator arm, a substantial impact on the result could not be entirely excluded. |
| **D2 judgment: Some concerns** | | | | |
| 3.1 | **PY** | “Regarding the secondary outcomes, actual observed data were used for the analysis without imputation for missing data.” Table 2 presents this outcome under the full-analysis-set group headings, and no additional outcome-specific losses were described. | Pei et al., pp. 76 and 80; Statistical Analysis and Table 2 | Outcome-specific denominators were not reported. Data were therefore judged probably available for all or nearly all participants in the electroacupuncture-versus-sham-electroacupuncture comparison, although this could not be verified directly. |
| 3.2 | **NA** | Not applicable based on the preceding signaling-question response. | - | The question was not reached in the RoB 2 pathway. |
| 3.3 | **NA** | Not applicable based on the preceding signaling-question response. | - | The question was not reached in the RoB 2 pathway. |
| 3.4 | **NA** | Not applicable based on the preceding signaling-question response. | - | The question was not reached in the RoB 2 pathway. |
| **D3 judgment: Low risk** | | | | |
| 4.1 | **N** | The outcomes were time to first flatus, first defecation, and first water intake, measured in hours after surgery. | Pei et al., pp. 75 and 80; Outcome Assessments and Table 2 | These were appropriate prespecified postoperative recovery measures. |
| 4.2 | **N** | “The time-related outcomes were initially recorded by the patients themselves; subsequently, nurses on each specific 8-hour shift reminded them to report.” | Pei et al., p. 75; Outcome Assessments | The same ascertainment method was applied across EA and SA groups. |
| 4.3 | **PN** | “Surgeons, outcome assessors, and statisticians were blinded,” and matched sham procedures were used. | Pei et al., p. 75; Randomization and Blinding, and Supplementary Material 2, eTable 4 | Outcome assessors were blinded and participants were probably blinded, although blinding success was formally evaluated at only two of the seven centers. |
| 4.4 | **NA** | Not applicable based on the preceding signaling-question response. | - | The question was not reached in the RoB 2 pathway. |
| 4.5 | **NA** | Not applicable based on the preceding signaling-question response. | - | The question was not reached in the RoB 2 pathway. |
| **D4 judgment: Low risk** | | | | |
| 5.1 | **Y** | The trial was prospectively registered (ChiCTR2100050660), and “The study protocol and statistical analysis plan are available as part of Supplementary Material 1.” | Pei et al., pp. 74 and 84; Study Design/Data Availability | A prospective registry, protocol, and SAP were available before final analysis. |
| 5.2 | **N** | The protocol-defined outcomes included time to first flatus, first defecation, and water intake, and Table 2 reports these prespecified measures. | Pei et al., pp. 75 and 80 | No result-based selection among multiple measurements was evident. |
| 5.3 | **N** | “The primary outcome was assessed using analysis of covariance…” and “The same approach applied to most secondary outcomes,” with a prespecified fixed-sequence/Bonferroni strategy. | Pei et al., pp. 75-76; Statistical Analysis | The analysis methods were prospectively structured and did not appear selected based on results. |
| **D5 judgment: Low risk** | | | | |
| **Overall risk-of-bias judgment: Some concerns. Randomization was centrally concealed, participants and outcome assessors were masked, and a prospective protocol and statistical analysis plan were available. Concerns arose in Domain 2 because the treatment allocation and analytic handling of five post-randomization exclusions were not reported. In addition, and unlike the primary outcome, outcome-specific denominators were not reported for this secondary outcome, although no additional outcome-specific losses were described.** | | | | |

**Pei et al. (2025)**

**Outcome: Time to first liquid intake (first water intake)**

*Assessment target: time to first water intake for EA versus sham EA. The trial also reported liquid-diet tolerance, but that distinct outcome was not pooled. The EA-versus-sham-EA difference for first water intake was -11.05 hours (P=.25).*

| **Domain / SQ** | **Response** | **Supporting quotation / source evidence** | **Location** | **Rationale** |
| --- | --- | --- | --- | --- |
| 1.1 | **Y** | “Randomization was stratified by enrollment site with a block size of 9…” | Pei et al., p. 75; Randomization and Blinding | A stratified block-randomization method was used. |
| 1.2 | **Y** | “Randomization and allocation concealment were conducted using the Electronic Data Capture System…” | Pei et al., p. 75; Randomization and Blinding | Central electronic allocation provided concealment. |
| 1.3 | **N** | “Baseline characteristics were similar across the groups, except for family history of gastric cancer.” | Pei et al., p. 76; Participants | A single modest imbalance was compatible with chance and was adjusted for in the analyses; it did not suggest compromised randomization. |
| **D1 judgment: Low risk** | | | | |
| 2.1 | **PN** | Matched blunt placebo needles were used. Blinding was formally assessed at two of the seven centers: 75 of 105 EA participants (71.43%) and 68 of 100 sham-EA participants (68.00%) believed they had received true EA (P = 0.79). Participants’ baseline expectations of acupuncture were also similar between the two groups (P = 0.49 and P = 0.35 for the two expectation items). | Pei et al., pp. 75-76; Randomization and Blinding, and Supplementary Material 2, eTables 3 and 4 | Similar treatment guesses and similar baseline expectations support successful participant masking in the EA-versus-sham-EA comparison, although the formal assessment was limited to two of the seven centers. |
| 2.2 | **Y** | “Surgeons, outcome assessors, and statisticians were blinded,” while licensed acupuncturists delivered EA or SA. | Pei et al., p. 75; Randomization and Blinding/Interventions | Acupuncturists necessarily knew the procedure, although other clinical and analytical personnel were masked. |
| 2.3 | **PN** | “All participants received perioperative standard care…” and EA/SA procedures were standardized. Some participants discontinued sessions or missed the 2-hour visit. | Pei et al., pp. 75 and 77 | Incomplete sessions were reported, but no important deviation caused by the trial context or imbalanced co-intervention was shown to affect the outcomes. |
| 2.4 | **NA** | Not applicable based on the preceding signaling-question response. | - | The question was not reached in the RoB 2 pathway. |
| 2.5 | **NA** | Not applicable based on the preceding signaling-question response. | - | The question was not reached in the RoB 2 pathway. |
| 2.6 | **PN** | The report describes five post-randomization exclusions before the arm-specific randomized totals are presented: one participant who underwent secondary surgery because of a severe postoperative complication, one erroneously randomized twice, and three found to have noncancerous gastric pathology. The report states that 585 participants were randomized overall; after these five exclusions the flow diagram displays group sizes of 195 (EA), 190 (sham EA) and 195 (standard care). One further participant in each of the EA and sham-EA arms was excluded from the full analysis set because no intervention was received and no efficacy outcome was recorded (194/195 and 189/190 analyzed). | Pei et al., pp. 76-77; Results and Figure 1 | Exclusion of participants whose ineligibility was ascertained only after randomization, and could not have been influenced by group assignment, may be acceptable; this applies to the duplicate randomization record and the three noncancerous pathologies. However, the exclusion following secondary surgery was based on a post-randomization clinical event. The treatment allocation of the five excluded participants was not reported, so the arm-specific randomized denominators could not be reconstructed. It could therefore not be confirmed that all eligible randomized participants were analyzed in the groups to which they were assigned. |
| 2.7 | **PN** | Two participants, one in each of the EA and sham-EA arms, were excluded from the full analysis set because no efficacy outcome data were recorded. The treatment allocation of the five earlier post-randomization exclusions was not reported. | Pei et al., pp. 76-77; Results and Figure 1 | Depending on the allocation of the five earlier exclusions, between 2 and 7 randomized participants were absent from the EA-versus-sham-EA analysis, corresponding to approximately 0.5% to 1.8% of the participants randomized to the two relevant arms. The exclusions were small in number, and no direct evidence indicated that they substantially altered the treatment estimate. However, because the allocation of the five earlier exclusions was not reported and may have been concentrated in the comparator arm, a substantial impact on the result could not be entirely excluded. |
| **D2 judgment: Some concerns** | | | | |
| 3.1 | **PY** | “Regarding the secondary outcomes, actual observed data were used for the analysis without imputation for missing data.” Table 2 presents this outcome under the full-analysis-set group headings, and no additional outcome-specific losses were described. | Pei et al., pp. 76 and 80; Statistical Analysis and Table 2 | Outcome-specific denominators were not reported. Data were therefore judged probably available for all or nearly all participants in the electroacupuncture-versus-sham-electroacupuncture comparison, although this could not be verified directly. |
| 3.2 | **NA** | Not applicable based on the preceding signaling-question response. | - | The question was not reached in the RoB 2 pathway. |
| 3.3 | **NA** | Not applicable based on the preceding signaling-question response. | - | The question was not reached in the RoB 2 pathway. |
| 3.4 | **NA** | Not applicable based on the preceding signaling-question response. | - | The question was not reached in the RoB 2 pathway. |
| **D3 judgment: Low risk** | | | | |
| 4.1 | **N** | The outcomes were time to first flatus, first defecation, and first water intake, measured in hours after surgery. | Pei et al., pp. 75 and 80; Outcome Assessments and Table 2 | These were appropriate prespecified postoperative recovery measures. |
| 4.2 | **N** | “The time-related outcomes were initially recorded by the patients themselves; subsequently, nurses on each specific 8-hour shift reminded them to report.” | Pei et al., p. 75; Outcome Assessments | The same ascertainment method was applied across EA and SA groups. |
| 4.3 | **PN** | “Surgeons, outcome assessors, and statisticians were blinded,” and matched sham procedures were used. | Pei et al., p. 75; Randomization and Blinding, and Supplementary Material 2, eTable 4 | Outcome assessors were blinded and participants were probably blinded, although blinding success was formally evaluated at only two of the seven centers. |
| 4.4 | **NA** | Not applicable based on the preceding signaling-question response. | - | The question was not reached in the RoB 2 pathway. |
| 4.5 | **NA** | Not applicable based on the preceding signaling-question response. | - | The question was not reached in the RoB 2 pathway. |
| **D4 judgment: Low risk** | | | | |
| 5.1 | **Y** | The trial was prospectively registered (ChiCTR2100050660), and “The study protocol and statistical analysis plan are available as part of Supplementary Material 1.” | Pei et al., pp. 74 and 84; Study Design/Data Availability | A prospective registry, protocol, and SAP were available before final analysis. |
| 5.2 | **N** | The trial separately reported “time to first water intake” and “time to first tolerance of liquid diet.” | Pei et al., pp. 75 and 80; Outcome Assessments and Table 2 | The review used first water intake or initiation of sips as the harmonized liquid-intake milestone; the liquid-diet-tolerance measure was not combined. Selection was based on outcome harmonization rather than on the magnitude or statistical significance of the observed results. |
| 5.3 | **N** | “The primary outcome was assessed using analysis of covariance…” and “The same approach applied to most secondary outcomes,” with a prespecified fixed-sequence/Bonferroni strategy. | Pei et al., pp. 75-76; Statistical Analysis | The analysis methods were prospectively structured and did not appear selected based on results. |
| **D5 judgment: Low risk** | | | | |
| **Overall risk-of-bias judgment: Some concerns. Randomization was centrally concealed, participants and outcome assessors were masked, and a prospective protocol and statistical analysis plan were available. Concerns arose in Domain 2 because the treatment allocation and analytic handling of five post-randomization exclusions were not reported. In addition, and unlike the primary outcome, outcome-specific denominators were not reported for this secondary outcome, although no additional outcome-specific losses were described.** | | | | |

**Pei et al. (2025)**

**Outcome: Length of hospital stay**

*Assessment target: duration of postoperative hospital stay for electroacupuncture versus sham electroacupuncture (10.25 ± 5.72 vs 9.82 ± 4.13 days), which was the hospital-stay result entered in the meta-analysis. The trial also reported total duration of hospital stay (15.37 ± 6.80 vs 15.09 ± 5.33 days) under a distinct definition; that measure was not pooled.*

| **Domain / SQ** | **Response** | **Supporting quotation / source evidence** | **Location** | **Rationale** |
| --- | --- | --- | --- | --- |
| 1.1 | **Y** | “Randomization was stratified by enrollment site with a block size of 9…” | Pei et al., p. 75; Randomization and Blinding | A stratified block-randomization method was used. |
| 1.2 | **Y** | “Randomization and allocation concealment were conducted using the Electronic Data Capture System…” | Pei et al., p. 75; Randomization and Blinding | Central electronic allocation provided concealment. |
| 1.3 | **N** | “Baseline characteristics were similar across the groups, except for family history of gastric cancer.” | Pei et al., p. 76; Participants | A single modest imbalance was compatible with chance and was adjusted for in the analyses; it did not suggest compromised randomization. |
| **D1 judgment: Low risk** | | | | |
| 2.1 | **PN** | Matched blunt placebo needles were used. Blinding was formally assessed at two of the seven centers: 75 of 105 EA participants (71.43%) and 68 of 100 sham-EA participants (68.00%) believed they had received true EA (P = 0.79). Participants’ baseline expectations of acupuncture were also similar between the two groups (P = 0.49 and P = 0.35 for the two expectation items). | Pei et al., pp. 75-76; Randomization and Blinding, and Supplementary Material 2, eTables 3 and 4 | Similar treatment guesses and similar baseline expectations support successful participant masking in the EA-versus-sham-EA comparison, although the formal assessment was limited to two of the seven centers. |
| 2.2 | **Y** | “Surgeons, outcome assessors, and statisticians were blinded,” while licensed acupuncturists delivered EA or SA. | Pei et al., p. 75; Randomization and Blinding/Interventions | Acupuncturists necessarily knew the procedure, although other clinical and analytical personnel were masked. |
| 2.3 | **PN** | “All participants received perioperative standard care…” and EA/SA procedures were standardized. Some participants discontinued sessions or missed the 2-hour visit. | Pei et al., pp. 75 and 77 | Incomplete sessions were reported, but no important deviation caused by the trial context or imbalanced co-intervention was shown to affect the outcomes. |
| 2.4 | **NA** | Not applicable based on the preceding signaling-question response. | - | The question was not reached in the RoB 2 pathway. |
| 2.5 | **NA** | Not applicable based on the preceding signaling-question response. | - | The question was not reached in the RoB 2 pathway. |
| 2.6 | **PN** | The report describes five post-randomization exclusions before the arm-specific randomized totals are presented: one participant who underwent secondary surgery because of a severe postoperative complication, one erroneously randomized twice, and three found to have noncancerous gastric pathology. The report states that 585 participants were randomized overall; after these five exclusions the flow diagram displays group sizes of 195 (EA), 190 (sham EA) and 195 (standard care). One further participant in each of the EA and sham-EA arms was excluded from the full analysis set because no intervention was received and no efficacy outcome was recorded (194/195 and 189/190 analyzed). | Pei et al., pp. 76-77; Results and Figure 1 | Exclusion of participants whose ineligibility was ascertained only after randomization, and could not have been influenced by group assignment, may be acceptable; this applies to the duplicate randomization record and the three noncancerous pathologies. However, the exclusion following secondary surgery was based on a post-randomization clinical event. The treatment allocation of the five excluded participants was not reported, so the arm-specific randomized denominators could not be reconstructed. It could therefore not be confirmed that all eligible randomized participants were analyzed in the groups to which they were assigned. |
| 2.7 | **PN** | Two participants, one in each of the EA and sham-EA arms, were excluded from the full analysis set because no efficacy outcome data were recorded. The treatment allocation of the five earlier post-randomization exclusions was not reported. | Pei et al., pp. 76-77; Results and Figure 1 | Depending on the allocation of the five earlier exclusions, between 2 and 7 randomized participants were absent from the EA-versus-sham-EA analysis, corresponding to approximately 0.5% to 1.8% of the participants randomized to the two relevant arms. The exclusions were small in number, and no direct evidence indicated that they substantially altered the treatment estimate. However, because the allocation of the five earlier exclusions was not reported and may have been concentrated in the comparator arm, a substantial impact on the result could not be entirely excluded. |
| **D2 judgment: Some concerns** | | | | |
| 3.1 | **PY** | “Regarding the secondary outcomes, actual observed data were used for the analysis without imputation for missing data.” Table 2 presents this outcome under the full-analysis-set group headings, and no additional outcome-specific losses were described. | Pei et al., pp. 76 and 80; Statistical Analysis and Table 2 | Outcome-specific denominators were not reported. Data were therefore judged probably available for all or nearly all participants in the electroacupuncture-versus-sham-electroacupuncture comparison, although this could not be verified directly. |
| 3.2 | **NA** | Not applicable based on the preceding signaling-question response. | - | The question was not reached in the RoB 2 pathway. |
| 3.3 | **NA** | Not applicable based on the preceding signaling-question response. | - | The question was not reached in the RoB 2 pathway. |
| 3.4 | **NA** | Not applicable based on the preceding signaling-question response. | - | The question was not reached in the RoB 2 pathway. |
| **D3 judgment: Low risk** | | | | |
| 4.1 | **N** | Secondary outcomes included duration of hospital stay and duration of postoperative hospital stay, the latter defined as the period from the end of laparoscopic surgery to the patient’s discharge. | Pei et al., p. 75; Outcome Assessments | Duration of postoperative hospital stay, measured in days from the end of surgery to discharge, is an appropriate outcome measure. |
| 4.2 | **N** | Participants were discharged using common criteria: no intravenous-fluid requirement, regular flatus and defecation, tolerance of oral liquid diet or enteral nutrition, controlled pain with oral analgesics, and full ambulation. | Pei et al., p. 75; Interventions | Standardized discharge criteria were applied across groups. |
| 4.3 | **N** | “Surgeons, outcome assessors, and statisticians were blinded to the treatment allocation.” | Pei et al., p. 75; Randomization and Blinding | Personnel involved in postoperative care and outcome assessment were blinded for the EA-versus-sham-EA comparison. |
| 4.4 | **NA** | Not applicable based on the preceding signaling-question response. | - | The question was not reached in the RoB 2 pathway. |
| 4.5 | **NA** | Not applicable based on the preceding signaling-question response. | - | The question was not reached in the RoB 2 pathway. |
| **D4 judgment: Low risk** | | | | |
| 5.1 | **Y** | The trial was prospectively registered (ChiCTR2100050660), and “The study protocol and statistical analysis plan are available as part of Supplementary Material 1.” | Pei et al., pp. 74 and 84; Study Design/Data Availability | A prospective registry, protocol, and SAP were available before final analysis. |
| 5.2 | **N** | Both total hospital stay and postoperative hospital stay were prespecified and reported with distinct definitions in Table 2. | Pei et al., pp. 75 and 80; Outcome Assessments and Table 2 | The review used postoperative hospital stay as the harmonized LOS measure when both postoperative and total hospital stay were reported. Both measures were available and transparently reported, and the choice was based on outcome harmonization rather than on the magnitude or statistical significance of the observed results. |
| 5.3 | **N** | Continuous secondary outcomes were analyzed using prespecified analysis of variance/Kruskal-Wallis and adjusted pairwise comparisons; Table 2 describes the corresponding analysis framework. | Pei et al., pp. 76 and 80; Statistical Analysis and Table 2 | The analysis approach was prospectively structured and did not appear selected on the basis of the observed result. |
| **D5 judgment: Low risk** | | | | |
| **Overall risk-of-bias judgment: Some concerns. Randomization was centrally concealed, and discharge was governed by prespecified criteria applied by blinded surgical staff, so the recorded postoperative stay was unlikely to be influenced by knowledge of the assigned intervention. Concerns arose in Domain 2 because the treatment allocation and analytic handling of five post-randomization exclusions were not reported, and outcome-specific denominators were not reported for this secondary outcome.** | | | | |

**Guan et al. (2025)**

**Outcome: Time to first flatus**

*Assessment target: time to first flatus in the electroacupuncture-versus-placebo-electroacupuncture comparison. Judgments were based on the Chinese Methods and Results, with supporting quotations given in Chinese and English translation. Seven of 44 randomized participants were excluded before analysis.*

| **Domain / SQ** | **Response** | **Supporting quotation / source evidence** | **Location** | **Rationale** |
| --- | --- | --- | --- | --- |
| 1.1 | **Y** | “使用 Stata12.0 软件，按 1∶1 比例将所有患者随机分为试验组和对照组” [Using Stata 12.0, all patients were randomized 1:1 into the experimental and control groups.] | Guan et al., p. 752; Section 1.1 | A computer-generated random allocation sequence was used. |
| 1.2 | **Y** | “将组别及处理方法信息装入标有 1–44 号序号的不透光信封中密封…在其接受针灸治疗前由信息保管者交予针灸医生并实施信封内分案” [Group and treatment information was sealed in sequentially numbered (1–44) opaque envelopes and released by an independent custodian to the acupuncturist only immediately before treatment.] | Guan et al., p. 752; Section 1.1 | Sequentially numbered, sealed opaque envelopes held by an independent custodian provided allocation concealment. |
| 1.3 | **N** | “两组患者性别、年龄、体质量指数（BMI）、病理分期、新辅助化疗、ASA 分级、手术方式、手术时间、并发症一般资料比较差异均无统计学意义（P>0.05），具有可比性” [Baseline characteristics did not differ significantly between the groups.] | Guan et al., p. 754; Section 3.3.1 and Table 1 | Baseline data did not suggest a problem with the randomization process. |
| **D1 judgment: Low risk** | | | | |
| 2.1 | **PY** | “对照组予安慰电针治疗，将一次性穴位贴敷电极片贴敷于穴位…打开仪器但不予任何刺激” and “如盲法设置方面采用非针刺安慰针灸…但有可能导致患者揭盲” [The control group received adhesive electrode pads with the device switched on but no stimulation; the authors noted that the non-penetrating placebo may have unblinded participants.] | Guan et al., pp. 753 and 755; Section 2.2 and Discussion | The experimental group received needle insertion to de qi whereas the control group received surface electrode pads only, and the authors themselves acknowledged possible unblinding. |
| 2.2 | **Y** | “予电针治疗，操作均由针灸科具备副主任及以上职称的中医师进行” [Electroacupuncture was delivered by senior acupuncture-department physicians.] | Guan et al., p. 753; Section 2.1 | The practitioners delivering the intervention necessarily knew the assigned procedure, although surgeons and statisticians were reported as masked. |
| 2.3 | **PN** | “剔除标准：①违反试验方案且沟通无效者” [Protocol violation was a prespecified exclusion criterion]; no protocol violation, crossover, or differential co-intervention was reported among the analyzed participants. | Guan et al., pp. 752 and 754; Sections 1.4 and 3.3.1 | No trial-context deviation from the intended interventions was reported; both groups were managed under the same ERAS pathway. |
| 2.4 | **NA** | Not applicable based on the preceding signaling-question response. | - | The question was not reached in the RoB 2 pathway. |
| 2.5 | **NA** | Not applicable based on the preceding signaling-question response. | - | The question was not reached in the RoB 2 pathway. |
| 2.6 | **N** | “共纳入 44 例患者，因肠鸣音采集不完整而剔除 4 例（试验组 1 例，对照组 3 例），3 例（试验组 2 例，对照组 1 例）患者因手术中探查证实为Ⅳ期肿瘤且无法切除而剔除，最终试验组 19 例、对照组 18 例完成试验” [Of 44 randomized participants, 4 were excluded for incomplete bowel-sound recording (1 experimental, 3 control) and 3 for intraoperative confirmation of unresectable stage IV disease (2 experimental, 1 control); 19 and 18 were analyzed.] | Guan et al., p. 754; Section 3.3.1 | Exclusion of the three participants whose ineligibility was confirmed only during surgery, and could not have been influenced by group assignment, may be acceptable. However, the four participants excluded because of incomplete recording of a different outcome were eligible randomized participants removed from the analysis, so the reported analysis did not estimate the effect of assignment. |
| 2.7 | **PY** | Four eligible randomized participants (one experimental and three control) were removed from an analysis of 19 versus 18 participants; the reported time to first flatus was (40.17 ± 14.71 vs 49.53 ± 18.79 h). | Guan et al., p. 754; Section 3.3.1 and Table 3 | In a trial of this size the exclusions were both proportionally large and unbalanced between groups, so a substantial impact on the reported difference in time to first flatus could not be excluded. |
| **D2 judgment: High risk** | | | | |
| 3.1 | **NI** | The report does not state whether time to first flatus data were available for the seven excluded participants. | Guan et al., p. 754; Section 3.3.1 | Availability of this outcome for the excluded participants could not be determined from the report. |
| 3.2 | **N** | No sensitivity analysis, and no analysis including all randomized participants, was reported. | Guan et al., p. 753; Section 3.2 | The report did not demonstrate that the complete-case result was unaffected by the absent participants. |
| 3.3 | **PN** | Exclusions were attributed to incomplete bowel-sound recording and to intraoperative confirmation of unresectable stage IV disease. | Guan et al., pp. 752 and 754; Sections 1.4 and 3.3.1 | Neither reason is plausibly related to the true value of time to first flatus. Removal of participants by the investigators was addressed in Domain 2 to avoid counting the same problem twice. |
| 3.4 | **NA** | Not applicable based on the preceding signaling-question response. | - | The question was not reached in the RoB 2 pathway. |
| **D3 judgment: Low risk** | | | | |
| 4.1 | **N** | “肛门首次排气、排便时间：记录自手术结束至肛门恢复排气、排便的时间” [Time to first anal flatus and defecation were recorded from the end of surgery.] | Guan et al., p. 753; Section 3.1.2 | Time to first flatus is a conventional postoperative recovery measure. |
| 4.2 | **N** | The same outcome definition and recording procedure were applied in both groups. | Guan et al., p. 753; Section 3.1.2 | No differential ascertainment method was described. |
| 4.3 | **PY** | “患者、手术医生及数据统计人员均无法获知分组方案” [Patients, surgeons and statisticians were unable to learn the allocation] but the Discussion states that the non-penetrating placebo “有可能导致患者揭盲” [may have unblinded patients]. | Guan et al., pp. 752 and 755; Section 1.1 and Discussion | Masking was intended, but the authors acknowledged that the placebo procedure may have been recognizable, and this outcome depends on participant report. |
| 4.4 | **Y** | Time to first flatus was a participant-dependent event recorded from the end of surgery. | Guan et al., p. 753; Section 3.1.2 | Knowledge of allocation could influence recognition or reporting of the event. |
| 4.5 | **PN** | Blinding success was not formally assessed, and participants’ beliefs about their allocation were not recorded. | Guan et al., pp. 752-753 and 755; Sections 1.1 and 2.2 and Discussion | Masking was intended, and patients, surgeons and statisticians were reported as blinded. However, the authors acknowledged that the non-penetrating placebo might have allowed participants to infer their assignment, and because blinding success was not formally assessed, influence on the timing of a participant-reported event could not be excluded, although no direct evidence demonstrated that such influence occurred. |
| **D4 judgment: Some concerns** | | | | |
| 5.1 | **NI** | No trial registration record, protocol, or statistical analysis plan was reported; the article states only institutional ethical approval. | Guan et al., p. 752; Section 1.1 | Pre-specification of the analysis before unblinded outcome data became available could not be verified. |
| 5.2 | **PN** | “肛门首次排气时间” was prespecified among the secondary outcome measures in the Methods and reported in Table 3. | Guan et al., pp. 753-754; Sections 3.1.2 and 3.3.3 | No evidence indicated selection from multiple eligible measurements within this outcome domain. |
| 5.3 | **PN** | “计量资料…组间比较采用两独立样本 t 检验” [Continuous variables were compared between groups using independent-samples t tests.] | Guan et al., p. 753; Section 3.2 | A single straightforward comparison was described for this continuous outcome. |
| **D5 judgment: Some concerns** | | | | |
| **Overall risk-of-bias judgment: High risk. Sequence generation and allocation concealment were adequately described, and the placebo procedure limited, although it did not eliminate, the risk of measurement bias. High risk was driven by Domain 2. Four eligible randomized participants were excluded from the time to first flatus analysis because of incomplete recording of a different outcome, and these exclusions were unbalanced between groups (one experimental and three control participants). In this small trial a substantial impact on the estimated effect of assignment could not be excluded.** | | | | |

**Guan et al. (2025)**

**Outcome: Time to first defecation**

*Assessment target: time to first defecation in the electroacupuncture-versus-placebo-electroacupuncture comparison. Judgments were based on the Chinese Methods and Results, with supporting quotations given in Chinese and English translation. Seven of 44 randomized participants were excluded before analysis.*

| **Domain / SQ** | **Response** | **Supporting quotation / source evidence** | **Location** | **Rationale** |
| --- | --- | --- | --- | --- |
| 1.1 | **Y** | “使用 Stata12.0 软件，按 1∶1 比例将所有患者随机分为试验组和对照组” [Using Stata 12.0, all patients were randomized 1:1 into the experimental and control groups.] | Guan et al., p. 752; Section 1.1 | A computer-generated random allocation sequence was used. |
| 1.2 | **Y** | “将组别及处理方法信息装入标有 1–44 号序号的不透光信封中密封…在其接受针灸治疗前由信息保管者交予针灸医生并实施信封内分案” [Group and treatment information was sealed in sequentially numbered (1–44) opaque envelopes and released by an independent custodian to the acupuncturist only immediately before treatment.] | Guan et al., p. 752; Section 1.1 | Sequentially numbered, sealed opaque envelopes held by an independent custodian provided allocation concealment. |
| 1.3 | **N** | “两组患者性别、年龄、体质量指数（BMI）、病理分期、新辅助化疗、ASA 分级、手术方式、手术时间、并发症一般资料比较差异均无统计学意义（P>0.05），具有可比性” [Baseline characteristics did not differ significantly between the groups.] | Guan et al., p. 754; Section 3.3.1 and Table 1 | Baseline data did not suggest a problem with the randomization process. |
| **D1 judgment: Low risk** | | | | |
| 2.1 | **PY** | “对照组予安慰电针治疗，将一次性穴位贴敷电极片贴敷于穴位…打开仪器但不予任何刺激” and “如盲法设置方面采用非针刺安慰针灸…但有可能导致患者揭盲” [The control group received adhesive electrode pads with the device switched on but no stimulation; the authors noted that the non-penetrating placebo may have unblinded participants.] | Guan et al., pp. 753 and 755; Section 2.2 and Discussion | The experimental group received needle insertion to de qi whereas the control group received surface electrode pads only, and the authors themselves acknowledged possible unblinding. |
| 2.2 | **Y** | “予电针治疗，操作均由针灸科具备副主任及以上职称的中医师进行” [Electroacupuncture was delivered by senior acupuncture-department physicians.] | Guan et al., p. 753; Section 2.1 | The practitioners delivering the intervention necessarily knew the assigned procedure, although surgeons and statisticians were reported as masked. |
| 2.3 | **PN** | “剔除标准：①违反试验方案且沟通无效者” [Protocol violation was a prespecified exclusion criterion]; no protocol violation, crossover, or differential co-intervention was reported among the analyzed participants. | Guan et al., pp. 752 and 754; Sections 1.4 and 3.3.1 | No trial-context deviation from the intended interventions was reported; both groups were managed under the same ERAS pathway. |
| 2.4 | **NA** | Not applicable based on the preceding signaling-question response. | - | The question was not reached in the RoB 2 pathway. |
| 2.5 | **NA** | Not applicable based on the preceding signaling-question response. | - | The question was not reached in the RoB 2 pathway. |
| 2.6 | **N** | “共纳入 44 例患者，因肠鸣音采集不完整而剔除 4 例（试验组 1 例，对照组 3 例），3 例（试验组 2 例，对照组 1 例）患者因手术中探查证实为Ⅳ期肿瘤且无法切除而剔除，最终试验组 19 例、对照组 18 例完成试验” [Of 44 randomized participants, 4 were excluded for incomplete bowel-sound recording (1 experimental, 3 control) and 3 for intraoperative confirmation of unresectable stage IV disease (2 experimental, 1 control); 19 and 18 were analyzed.] | Guan et al., p. 754; Section 3.3.1 | Exclusion of the three participants whose ineligibility was confirmed only during surgery, and could not have been influenced by group assignment, may be acceptable. However, the four participants excluded because of incomplete recording of a different outcome were eligible randomized participants removed from the analysis, so the reported analysis did not estimate the effect of assignment. |
| 2.7 | **PY** | Four eligible randomized participants (one experimental and three control) were removed from an analysis of 19 versus 18 participants; the reported time to first defecation was (81.40 ± 22.18 vs 87.90 ± 35.77 h). | Guan et al., p. 754; Section 3.3.1 and Table 3 | In a trial of this size the exclusions were both proportionally large and unbalanced between groups, so a substantial impact on the reported difference in time to first defecation could not be excluded. |
| **D2 judgment: High risk** | | | | |
| 3.1 | **NI** | The report does not state whether time to first defecation data were available for the seven excluded participants. | Guan et al., p. 754; Section 3.3.1 | Availability of this outcome for the excluded participants could not be determined from the report. |
| 3.2 | **N** | No sensitivity analysis, and no analysis including all randomized participants, was reported. | Guan et al., p. 753; Section 3.2 | The report did not demonstrate that the complete-case result was unaffected by the absent participants. |
| 3.3 | **PN** | Exclusions were attributed to incomplete bowel-sound recording and to intraoperative confirmation of unresectable stage IV disease. | Guan et al., pp. 752 and 754; Sections 1.4 and 3.3.1 | Neither reason is plausibly related to the true value of time to first defecation. Removal of participants by the investigators was addressed in Domain 2 to avoid counting the same problem twice. |
| 3.4 | **NA** | Not applicable based on the preceding signaling-question response. | - | The question was not reached in the RoB 2 pathway. |
| **D3 judgment: Low risk** | | | | |
| 4.1 | **N** | “肛门首次排气、排便时间：记录自手术结束至肛门恢复排气、排便的时间” [Time to first anal flatus and defecation were recorded from the end of surgery.] | Guan et al., p. 753; Section 3.1.2 | Time to first defecation is a conventional postoperative recovery measure. |
| 4.2 | **N** | The same outcome definition and recording procedure were applied in both groups. | Guan et al., p. 753; Section 3.1.2 | No differential ascertainment method was described. |
| 4.3 | **PY** | “患者、手术医生及数据统计人员均无法获知分组方案” [Patients, surgeons and statisticians were unable to learn the allocation] but the Discussion states that the non-penetrating placebo “有可能导致患者揭盲” [may have unblinded patients]. | Guan et al., pp. 752 and 755; Section 1.1 and Discussion | Masking was intended, but the authors acknowledged that the placebo procedure may have been recognizable, and this outcome depends on participant report. |
| 4.4 | **Y** | Time to first defecation was a participant-dependent event recorded from the end of surgery. | Guan et al., p. 753; Section 3.1.2 | Knowledge of allocation could influence recognition or reporting of the event. |
| 4.5 | **PN** | Blinding success was not formally assessed, and participants’ beliefs about their allocation were not recorded. | Guan et al., pp. 752-753 and 755; Sections 1.1 and 2.2 and Discussion | Masking was intended, and patients, surgeons and statisticians were reported as blinded. However, the authors acknowledged that the non-penetrating placebo might have allowed participants to infer their assignment, and because blinding success was not formally assessed, influence on the timing of a participant-reported event could not be excluded, although no direct evidence demonstrated that such influence occurred. |
| **D4 judgment: Some concerns** | | | | |
| 5.1 | **NI** | No trial registration record, protocol, or statistical analysis plan was reported; the article states only institutional ethical approval. | Guan et al., p. 752; Section 1.1 | Pre-specification of the analysis before unblinded outcome data became available could not be verified. |
| 5.2 | **PN** | “肛门首次排便时间” was prespecified among the secondary outcome measures in the Methods and reported in Table 3. | Guan et al., pp. 753-754; Sections 3.1.2 and 3.3.3 | No evidence indicated selection from multiple eligible measurements within this outcome domain. |
| 5.3 | **PN** | “计量资料…组间比较采用两独立样本 t 检验” [Continuous variables were compared between groups using independent-samples t tests.] | Guan et al., p. 753; Section 3.2 | A single straightforward comparison was described for this continuous outcome. |
| **D5 judgment: Some concerns** | | | | |
| **Overall risk-of-bias judgment: High risk. Sequence generation and allocation concealment were adequately described, and the placebo procedure limited, although it did not eliminate, the risk of measurement bias. High risk was driven by Domain 2. Four eligible randomized participants were excluded from the time to first defecation analysis because of incomplete recording of a different outcome, and these exclusions were unbalanced between groups (one experimental and three control participants). In this small trial a substantial impact on the estimated effect of assignment could not be excluded.** | | | | |

**Jung et al. (2017)**

**Outcome: Time to first flatus**

*Assessment target: time to first flatus in the acupuncture-versus-no-acupuncture comparison; four of 40 randomized participants were excluded.*

| **Domain / SQ** | **Response** | **Supporting quotation / source evidence** | **Location** | **Rationale** |
| --- | --- | --- | --- | --- |
| 1.1 | **PY** | “The patients were randomized before surgery using simple randomization…” | Jung et al., p. 13; Study design | The report described simple randomization but did not state the exact sequence-generation mechanism. |
| 1.2 | **NI** | No allocation-concealment method was described. | Jung et al., Study design, p. 13 | Concealment before assignment could not be verified. |
| 1.3 | **N** | “The patient characteristics were well balanced between the groups…” | Jung et al., p. 15; Patient demographics | No baseline difference suggested a randomization problem. |
| **D1 judgment: Some concerns** | | | | |
| 2.1 | **Y** | The trial compared acupuncture with a non-acupuncture group, with no sham procedure. | Jung et al., pp. 13 and 18 | Participants were aware whether they received daily acupuncture. |
| 2.2 | **Y** | Acupuncture was administered by Korean traditional medicine doctors; the control group received no acupuncture. | Jung et al., p. 13; Acupuncture interventions | Intervention providers and clinical personnel could identify assignment. |
| 2.3 | **NI** | “Two patients in each group were withdrawn due to discontinued intervention or refusal to participate…” | Jung et al., pp. 14-15; Results | Withdrawals and non-adherence occurred, but the report did not establish whether they arose because of the trial context. |
| 2.4 | **NA** | Not applicable based on the preceding signaling-question response. | - | The question was not reached in the RoB 2 pathway. |
| 2.5 | **NA** | Not applicable based on the preceding signaling-question response. | - | The question was not reached in the RoB 2 pathway. |
| 2.6 | **N** | “Thus, 36 recruited patients were available for the analyses of primary and secondary outcomes.” | Jung et al., p. 15; Results | The analysis excluded four randomized participants and was not an assignment-effect/ITT analysis. |
| 2.7 | **PY** | Four of 40 randomized participants (10%) were excluded. For time to first flatus, the complete-case estimate was based on only 18 participants per group. | Jung et al., pp. 14-15; Results and Table 3 | In this small trial the complete-case difference was clearly significant (2.33 ± 0.49 vs 2.94 ± 0.80 days; P=.009). Nevertheless, removing four of 40 randomized participants from an analysis of 18 per group could still shift the effect estimate, and the direction of that shift could not be determined from the report. |
| **D2 judgment: High risk** | | | | |
| 3.1 | **N** | Forty participants were randomized, but 36 were included in the analyses of primary and secondary outcomes. | Jung et al., pp. 14-15; Results | Outcome data were missing for 10% of randomized participants. |
| 3.2 | **N** | No outcome-specific sensitivity analysis or analysis including all randomized participants was reported. | Jung et al., Results, pp. 14-15 | The report did not demonstrate that the complete-case result was unaffected by missing data. |
| 3.3 | **PY** | “Two patients in each group were withdrawn due to discontinued intervention or refusal to participate.” | Jung et al., pp. 14-15; Results | Missingness could have been related to intervention experience, tolerance, or postoperative recovery. |
| 3.4 | **PN** | Withdrawals were numerically balanced (two per group), and the report did not link withdrawal directly to the unobserved outcome values. | Jung et al., pp. 14-15 | Outcome-dependent missingness was possible but not shown to be likely. |
| **D3 judgment: Some concerns** | | | | |
| 4.1 | **N** | Secondary outcomes included time to first flatus, first defecation, and start of sips of water. | Jung et al., pp. 11 and 13 | These were relevant postoperative recovery outcomes. |
| 4.2 | **N** | The same postoperative protocol and outcome definitions were applied in both groups. | Jung et al., p. 13; Study design | No different measurement procedure by group was described. |
| 4.3 | **Y** | The study used a non-acupuncture control without sham treatment; the authors noted that the acupuncture group received more physician attention. | Jung et al., p. 18; Limitations | Participants and attending clinicians/outcome assessors were likely aware of assignment. |
| 4.4 | **Y** | Time to first flatus was participant-reported in a non-sham, open-label comparison. | Jung et al., pp. 13 and 18 | Knowledge of assignment could influence recognition or reporting of the event. |
| 4.5 | **PY** | The authors acknowledged that the absence of sham acupuncture and unequal physician-patient interaction could have affected results. | Jung et al., p. 18; Limitations | Influence on participant-reported flatus was considered likely. |
| **D4 judgment: High risk** | | | | |
| 5.1 | **NI** | No prospective registry record, protocol, or statistical analysis plan was reported in the article. | Jung et al., article report | Pre-specification before outcome data were available could not be verified. |
| 5.2 | **PN** | The secondary outcomes were listed in advance in the Methods as flatus, defecation, water intake, soft diet, and hospital stay. | Jung et al., pp. 11 and 13 | There was no clear evidence of selecting among multiple measurements within each assessed outcome domain. |
| 5.3 | **PN** | The article described two-sample t-tests for operative outcomes. | Jung et al., p. 14; Statistical analysis | No multiple eligible analyses for the assessed numerical results were apparent. |
| **D5 judgment: Some concerns** | | | | |
| **Overall risk-of-bias judgment: High risk. High risk was driven by exclusion of 10% of randomized participants from a small trial and likely measurement bias in an open-label comparison.** | | | | |

**Jung et al. (2017)**

**Outcome: Time to first defecation**

*Assessment target: time to first defecation in the acupuncture-versus-no-acupuncture comparison; four of 40 randomized participants were excluded.*

| **Domain / SQ** | **Response** | **Supporting quotation / source evidence** | **Location** | **Rationale** |
| --- | --- | --- | --- | --- |
| 1.1 | **PY** | “The patients were randomized before surgery using simple randomization…” | Jung et al., p. 13; Study design | The report described simple randomization but did not state the exact sequence-generation mechanism. |
| 1.2 | **NI** | No allocation-concealment method was described. | Jung et al., Study design, p. 13 | Concealment before assignment could not be verified. |
| 1.3 | **N** | “The patient characteristics were well balanced between the groups…” | Jung et al., p. 15; Patient demographics | No baseline difference suggested a randomization problem. |
| **D1 judgment: Some concerns** | | | | |
| 2.1 | **Y** | The trial compared acupuncture with a non-acupuncture group, with no sham procedure. | Jung et al., pp. 13 and 18 | Participants were aware whether they received daily acupuncture. |
| 2.2 | **Y** | Acupuncture was administered by Korean traditional medicine doctors; the control group received no acupuncture. | Jung et al., p. 13; Acupuncture interventions | Intervention providers and clinical personnel could identify assignment. |
| 2.3 | **NI** | “Two patients in each group were withdrawn due to discontinued intervention or refusal to participate…” | Jung et al., pp. 14-15; Results | Withdrawals and non-adherence occurred, but the report did not establish whether they arose because of the trial context. |
| 2.4 | **NA** | Not applicable based on the preceding signaling-question response. | - | The question was not reached in the RoB 2 pathway. |
| 2.5 | **NA** | Not applicable based on the preceding signaling-question response. | - | The question was not reached in the RoB 2 pathway. |
| 2.6 | **N** | “Thus, 36 recruited patients were available for the analyses of primary and secondary outcomes.” | Jung et al., p. 15; Results | The analysis excluded four randomized participants and was not an assignment-effect/ITT analysis. |
| 2.7 | **PY** | Four of 40 randomized participants (10%) were excluded. For time to first defecation, the complete-case estimate was based on only 18 participants per group. | Jung et al., pp. 14-15; Results and Table 3 | In this small trial the complete-case difference lay at the conventional significance threshold (2.94 ± 0.64 vs 3.39 ± 0.69 days; P=.054), so exclusion of four of 40 randomized participants could materially change the effect estimate for this outcome. |
| **D2 judgment: High risk** | | | | |
| 3.1 | **N** | Forty participants were randomized, but 36 were included in the analyses of primary and secondary outcomes. | Jung et al., pp. 14-15; Results | Outcome data were missing for 10% of randomized participants. |
| 3.2 | **N** | No outcome-specific sensitivity analysis or analysis including all randomized participants was reported. | Jung et al., Results, pp. 14-15 | The report did not demonstrate that the complete-case result was unaffected by missing data. |
| 3.3 | **PY** | “Two patients in each group were withdrawn due to discontinued intervention or refusal to participate.” | Jung et al., pp. 14-15; Results | Missingness could have been related to intervention experience, tolerance, or postoperative recovery. |
| 3.4 | **PN** | Withdrawals were numerically balanced (two per group), and the report did not link withdrawal directly to the unobserved outcome values. | Jung et al., pp. 14-15 | Outcome-dependent missingness was possible but not shown to be likely. |
| **D3 judgment: Some concerns** | | | | |
| 4.1 | **N** | Secondary outcomes included time to first flatus, first defecation, and start of sips of water. | Jung et al., pp. 11 and 13 | These were relevant postoperative recovery outcomes. |
| 4.2 | **N** | The same postoperative protocol and outcome definitions were applied in both groups. | Jung et al., p. 13; Study design | No different measurement procedure by group was described. |
| 4.3 | **Y** | The study used a non-acupuncture control without sham treatment; the authors noted that the acupuncture group received more physician attention. | Jung et al., p. 18; Limitations | Participants and attending clinicians/outcome assessors were likely aware of assignment. |
| 4.4 | **Y** | Time to first defecation was participant-reported in a non-sham, open-label comparison. | Jung et al., pp. 13 and 18 | Knowledge of assignment could influence recognition or reporting of the event. |
| 4.5 | **PY** | The complete-case result was near the conventional significance threshold (P=.054), and the authors acknowledged possible attention-related effects. | Jung et al., pp. 15 and 18; Table 3 and Limitations | Influence on participant reporting was considered likely. |
| **D4 judgment: High risk** | | | | |
| 5.1 | **NI** | No prospective registry record, protocol, or statistical analysis plan was reported in the article. | Jung et al., article report | Pre-specification before outcome data were available could not be verified. |
| 5.2 | **PN** | The secondary outcomes were listed in advance in the Methods as flatus, defecation, water intake, soft diet, and hospital stay. | Jung et al., pp. 11 and 13 | There was no clear evidence of selecting among multiple measurements within each assessed outcome domain. |
| 5.3 | **PN** | The article described two-sample t-tests for operative outcomes. | Jung et al., p. 14; Statistical analysis | No multiple eligible analyses for the assessed numerical results were apparent. |
| **D5 judgment: Some concerns** | | | | |
| **Overall risk-of-bias judgment: High risk. High risk was driven by exclusion of 10% of randomized participants from a small trial and likely measurement bias in an open-label comparison.** | | | | |

**Jung et al. (2017)**

**Outcome: Time to first liquid intake (start of sips of water)**

*Assessment target: time to first liquid intake (start of sips of water) in the acupuncture-versus-no-acupuncture comparison; four of 40 randomized participants were excluded.*

| **Domain / SQ** | **Response** | **Supporting quotation / source evidence** | **Location** | **Rationale** |
| --- | --- | --- | --- | --- |
| 1.1 | **PY** | “The patients were randomized before surgery using simple randomization…” | Jung et al., p. 13; Study design | The report described simple randomization but did not state the exact sequence-generation mechanism. |
| 1.2 | **NI** | No allocation-concealment method was described. | Jung et al., Study design, p. 13 | Concealment before assignment could not be verified. |
| 1.3 | **N** | “The patient characteristics were well balanced between the groups…” | Jung et al., p. 15; Patient demographics | No baseline difference suggested a randomization problem. |
| **D1 judgment: Some concerns** | | | | |
| 2.1 | **Y** | The trial compared acupuncture with a non-acupuncture group, with no sham procedure. | Jung et al., pp. 13 and 18 | Participants were aware whether they received daily acupuncture. |
| 2.2 | **Y** | Acupuncture was administered by Korean traditional medicine doctors; the control group received no acupuncture. | Jung et al., p. 13; Acupuncture interventions | Intervention providers and clinical personnel could identify assignment. |
| 2.3 | **NI** | “Two patients in each group were withdrawn due to discontinued intervention or refusal to participate…” | Jung et al., pp. 14-15; Results | Withdrawals and non-adherence occurred, but the report did not establish whether they arose because of the trial context. |
| 2.4 | **NA** | Not applicable based on the preceding signaling-question response. | - | The question was not reached in the RoB 2 pathway. |
| 2.5 | **NA** | Not applicable based on the preceding signaling-question response. | - | The question was not reached in the RoB 2 pathway. |
| 2.6 | **N** | “Thus, 36 recruited patients were available for the analyses of primary and secondary outcomes.” | Jung et al., p. 15; Results | The analysis excluded four randomized participants and was not an assignment-effect/ITT analysis. |
| 2.7 | **PY** | Four of 40 randomized participants (10%) were excluded. For time to first liquid intake (start of sips of water), the complete-case estimate was based on only 18 participants per group. | Jung et al., pp. 14-15; Results and Table 3 | In this small trial the complete-case difference lay at the conventional significance threshold (3.22 ± 0.65 vs 3.67 ± 0.59 days; P=.055), so exclusion of four of 40 randomized participants could materially change the effect estimate for this outcome. |
| **D2 judgment: High risk** | | | | |
| 3.1 | **N** | Forty participants were randomized, but 36 were included in the analyses of primary and secondary outcomes. | Jung et al., pp. 14-15; Results | Outcome data were missing for 10% of randomized participants. |
| 3.2 | **N** | No outcome-specific sensitivity analysis or analysis including all randomized participants was reported. | Jung et al., Results, pp. 14-15 | The report did not demonstrate that the complete-case result was unaffected by missing data. |
| 3.3 | **PY** | “Two patients in each group were withdrawn due to discontinued intervention or refusal to participate.” | Jung et al., pp. 14-15; Results | Missingness could have been related to intervention experience, tolerance, or postoperative recovery. |
| 3.4 | **PN** | Withdrawals were numerically balanced (two per group), and the report did not link withdrawal directly to the unobserved outcome values. | Jung et al., pp. 14-15 | Outcome-dependent missingness was possible but not shown to be likely. |
| **D3 judgment: Some concerns** | | | | |
| 4.1 | **N** | Secondary outcomes included time to first flatus, first defecation, and start of sips of water. | Jung et al., pp. 11 and 13 | These were relevant postoperative recovery outcomes. |
| 4.2 | **N** | The same postoperative protocol and outcome definitions were applied in both groups. | Jung et al., p. 13; Study design | No different measurement procedure by group was described. |
| 4.3 | **Y** | The study used a non-acupuncture control without sham treatment; the authors noted that the acupuncture group received more physician attention. | Jung et al., p. 18; Limitations | Participants and attending clinicians/outcome assessors were likely aware of assignment. |
| 4.4 | **Y** | The authors stated that “the time to resume diet can be influenced by the patient’s perception and can be easily manipulated by the attending clinician.” | Jung et al., p. 17; Discussion | Knowledge of assignment could influence the clinician-dependent start of sips of water. |
| 4.5 | **PY** | Start of sips of water was close to the significance threshold (P=.055), and the authors explicitly acknowledged potential manipulation of diet timing. | Jung et al., pp. 15 and 17; Table 3 and Discussion | Influence from allocation knowledge was considered likely. |
| **D4 judgment: High risk** | | | | |
| 5.1 | **NI** | No prospective registry record, protocol, or statistical analysis plan was reported in the article. | Jung et al., article report | Pre-specification before outcome data were available could not be verified. |
| 5.2 | **PN** | The secondary outcomes were listed in advance in the Methods as flatus, defecation, water intake, soft diet, and hospital stay. | Jung et al., pp. 11 and 13 | There was no clear evidence of selecting among multiple measurements within each assessed outcome domain. |
| 5.3 | **PN** | The article described two-sample t-tests for operative outcomes. | Jung et al., p. 14; Statistical analysis | No multiple eligible analyses for the assessed numerical results were apparent. |
| **D5 judgment: Some concerns** | | | | |
| **Overall risk-of-bias judgment: High risk. High risk was driven by exclusion of 10% of randomized participants from a small trial and likely measurement bias in an open-label comparison.** | | | | |

**Jung et al. (2017)**

**Outcome: Length of hospital stay**

*Assessment target: LOS in the acupuncture-versus-no-acupuncture comparison; four of 40 randomized participants were excluded from the complete-case analysis.*

| **Domain / SQ** | **Response** | **Supporting quotation / source evidence** | **Location** | **Rationale** |
| --- | --- | --- | --- | --- |
| 1.1 | **PY** | “The patients were randomized before surgery using simple randomization…” | Jung et al., p. 13; Study design | The report described simple randomization but did not state the exact sequence-generation mechanism. |
| 1.2 | **NI** | No allocation-concealment method was described. | Jung et al., Study design, p. 13 | Concealment before assignment could not be verified. |
| 1.3 | **N** | “The patient characteristics were well balanced between the groups…” | Jung et al., p. 15; Patient demographics | No baseline difference suggested a randomization problem. |
| **D1 judgment: Some concerns** | | | | |
| 2.1 | **Y** | The trial compared acupuncture with a non-acupuncture group, with no sham procedure. | Jung et al., pp. 13 and 18 | Participants were aware whether they received daily acupuncture. |
| 2.2 | **Y** | Acupuncture was administered by Korean traditional medicine doctors; the control group received no acupuncture. | Jung et al., p. 13; Acupuncture interventions | Intervention providers and clinical personnel could identify assignment. |
| 2.3 | **NI** | “Two patients in each group were withdrawn due to discontinued intervention or refusal to participate…” | Jung et al., pp. 14-15; Results | Withdrawals and non-adherence occurred, but the report did not establish whether they arose because of the trial context. |
| 2.4 | **NA** | Not applicable based on the preceding signaling-question response. | - | The question was not reached in the RoB 2 pathway. |
| 2.5 | **NA** | Not applicable based on the preceding signaling-question response. | - | The question was not reached in the RoB 2 pathway. |
| 2.6 | **N** | “Thus, 36 recruited patients were available for the analyses of primary and secondary outcomes.” | Jung et al., p. 15; Results | The analysis excluded four randomized participants and was not an assignment-effect/ITT analysis. |
| 2.7 | **PY** | Four of 40 randomized participants (10%) were excluded. For length of hospital stay, the complete-case estimate was based on only 18 participants per group. | Jung et al., pp. 14-15; Results and Table 3 | For length of hospital stay the complete-case means were 9.72 ± 2.29 versus 10.17 ± 2.38 days. Removal of four of 40 randomized participants represented a non-trivial proportion of a small sample relative to this variability and could materially change the mean difference, although the reported difference was small and not significant (P=.572). |
| **D2 judgment: High risk** | | | | |
| 3.1 | **N** | Forty participants were randomized, but 36 were included in the analyses of primary and secondary outcomes. | Jung et al., pp. 14-15; Results | Outcome data were missing for 10% of randomized participants. |
| 3.2 | **N** | No outcome-specific sensitivity analysis or analysis including all randomized participants was reported. | Jung et al., Results, pp. 14-15 | The report did not demonstrate that the complete-case result was unaffected by missing data. |
| 3.3 | **PY** | “Two patients in each group were withdrawn due to discontinued intervention or refusal to participate.” | Jung et al., pp. 14-15; Results | Missingness could have been related to intervention experience, tolerance, or postoperative recovery. |
| 3.4 | **PN** | Withdrawals were numerically balanced (two per group), and the report did not link withdrawal directly to the unobserved outcome values. | Jung et al., pp. 14-15 | Outcome-dependent missingness was possible but not shown to be likely. |
| **D3 judgment: Some concerns** | | | | |
| 4.1 | **N** | The secondary outcomes included “hospital stay.” | Jung et al., pp. 11 and 13; Abstract and Outcomes | Length of hospital stay is an appropriate postoperative outcome. |
| 4.2 | **N** | “All patients in both groups received the same postoperative management,” and patients were discharged after tolerating a solid diet and becoming fully ambulatory. | Jung et al., p. 13; Study design | Common postoperative care and discharge criteria were described for both groups. |
| 4.3 | **Y** | The study compared acupuncture with a non-acupuncture group without a sham procedure. | Jung et al., pp. 13 and 18 | Participants and attending clinicians were aware of assignment. |
| 4.4 | **Y** | Patients were discharged after tolerating solid diet and being fully ambulatory, and treatment allocation was apparent. | Jung et al., pp. 13 and 18 | The clinical discharge decision could be influenced by knowledge of assignment. |
| 4.5 | **PN** | Common discharge criteria were reported, and the LOS difference was small and non-significant (9.72 vs 10.17 days; P=.572). | Jung et al., pp. 13 and 15; Table 3 | Influence was possible but not shown to be likely for LOS. |
| **D4 judgment: Some concerns** | | | | |
| 5.1 | **NI** | No prospective registry record, protocol, or statistical analysis plan was reported in the article. | Jung et al., article report | Pre-specification before outcome data were available could not be verified. |
| 5.2 | **PN** | Hospital stay was explicitly listed as a secondary outcome in the Methods. | Jung et al., pp. 11 and 13 | There was no clear evidence that the reported hospital-stay measurement was selected from multiple eligible measurements. |
| 5.3 | **PN** | The article described two-sample t-tests for operative outcomes. | Jung et al., p. 14; Statistical analysis | No multiple eligible analyses for the hospital-stay result were apparent. |
| **D5 judgment: Some concerns** | | | | |
| **Overall risk-of-bias judgment: High risk. Overall high risk was driven by the inappropriate exclusion of 10% of randomized participants from the assignment-effect analysis; LOS measurement itself raised some, rather than high, concern.** | | | | |

**Cai et al. (2025)**

**Outcome: Time to first flatus**

*Assessment target: time to first flatus in the electroacupuncture-versus-sham-electroacupuncture comparison, reported as median (interquartile range) [42.7 (31.7-48.2) vs 46.8 (43.7-64.6) h]. Means and standard deviations were estimated from the reported medians and interquartile ranges using the method of Wan et al. before quantitative synthesis.*

| **Domain / SQ** | **Response** | **Supporting quotation / source evidence** | **Location** | **Rationale** |
| --- | --- | --- | --- | --- |
| 1.1 | **Y** | “The random number sequence was generated by an independent statistician who did not participate in any other part of the study,” and patients were “randomly allocated into acupuncture or sham acupuncture group in a ratio of 1:1 by block randomization.” | Cai et al., p. 5166; Randomization and Blinding | An independent statistician generated a block-randomized allocation sequence. |
| 1.2 | **Y** | “The sequential number was sealed into opaque envelopes and kept by the project coordinator.” | Cai et al., p. 5166; Randomization and Blinding | Sequentially numbered, sealed opaque envelopes held by an independent coordinator provided allocation concealment. |
| 1.3 | **N** | “There was no statistical difference in baseline characteristics between the groups (P > 0.05).” | Cai et al., p. 5168; Patient Characteristics and Table 1 | Baseline data did not suggest a problem with the randomization process. |
| **D1 judgment: Low risk** | | | | |
| 2.1 | **PN** | Sham electroacupuncture used identical needling without current, and “The patients had an eye patch placed over their eyes to achieve blinding.” | Cai et al., pp. 5166-5167; Randomization and Blinding/Interventions | Both groups received needle insertion and visual cues were masked, so participants were probably unaware of assignment; blinding success was not formally assessed. |
| 2.2 | **Y** | “Acupuncturists could not be blinded owing to the acupuncture procedure.” | Cai et al., p. 5166; Randomization and Blinding | The practitioners delivering the intervention necessarily knew the assigned procedure. |
| 2.3 | **NI** | The flow diagram records one discontinuation in the electroacupuncture arm and two in the sham arm, and the Results attribute three exclusions to “incomplete treatment,” without explaining why treatment was incomplete. | Cai et al., pp. 5167-5168; Figure 1 and Patient Characteristics | Post-randomization departures occurred, but their relationship to the trial context could not be determined. |
| 2.4 | **NA** | Not applicable based on the preceding signaling-question response. | - | The question was not reached in the RoB 2 pathway. |
| 2.5 | **NA** | Not applicable based on the preceding signaling-question response. | - | The question was not reached in the RoB 2 pathway. |
| 2.6 | **N** | “In total, 105 patients met the inclusion criteria. Of them, seven were excluded because of widespread tumor metastasis (n = 3), refusal of acupuncture therapy (n = 1), and incomplete treatment (n = 3).” The flow diagram shows 101 randomized (51 and 50) and 98 analyzed (50 and 48). | Cai et al., pp. 5167-5168; Figure 1 and Patient Characteristics | Three randomized participants were excluded because they did not complete the assigned treatment. Exclusion on the basis of treatment received corresponds to a per-protocol analysis rather than an estimate of the effect of assignment. |
| 2.7 | **PN** | Three of 101 randomized participants (one electroacupuncture and two sham) were excluded, corresponding to approximately 3% of the randomized sample. | Cai et al., p. 5167; Figure 1 | The exclusions were few and approximately balanced between groups, so a substantial impact on the result was unlikely. |
| **D2 judgment: Some concerns** | | | | |
| 3.1 | **Y** | Ninety-eight of 101 randomized participants were included in the analysis (50 and 48). | Cai et al., p. 5167; Figure 1 | Outcome data were available for 97% of randomized participants, considered nearly all. |
| 3.2 | **NA** | Not applicable based on the preceding signaling-question response. | - | The question was not reached in the RoB 2 pathway. |
| 3.3 | **NA** | Not applicable based on the preceding signaling-question response. | - | The question was not reached in the RoB 2 pathway. |
| 3.4 | **NA** | Not applicable based on the preceding signaling-question response. | - | The question was not reached in the RoB 2 pathway. |
| **D3 judgment: Low risk** | | | | |
| 4.1 | **N** | “The primary outcome was first flatus time.” | Cai et al., p. 5168; Outcomes | Time to first flatus is a conventional postoperative recovery measure. |
| 4.2 | **N** | All participants were managed under the same ERAS pathway, “in which there was no difference in narcotic use, pain medication, and pro-motility agents.” | Cai et al., p. 5167; Interventions | The same ascertainment conditions applied to both groups. |
| 4.3 | **NI** | “The assessors of outcome measures and the statisticians performing the data analysis were blinded,” and “The patients had an eye patch placed over their eyes to achieve blinding.” Both groups received needle insertion, but electrical current (10 Hz, 2–5 mA) was delivered only in the electroacupuncture group. Blinding success was not assessed. | Cai et al., pp. 5166-5167; Randomization and Blinding/Interventions | Outcome assessors and statisticians were masked and visual cues were covered, but the presence or absence of electrical stimulation was a perceptible difference between the arms and blinding success was not formally assessed, so it could not be established whether participants remained unaware of their assignment. |
| 4.4 | **Y** | Time to first flatus was a participant-dependent event. | Cai et al., p. 5168; Outcomes | Knowledge of allocation could influence recognition or reporting of the event. |
| 4.5 | **PN** | No formal assessment of blinding success or of participants’ beliefs about their allocation was reported. | Cai et al., pp. 5166-5168; Randomization and Blinding and Outcomes | Influence was possible, but participants received identical needling in both arms and outcome assessors were masked, and there was no direct evidence that participants recognized their allocation or that reporting was altered. |
| **D4 judgment: Some concerns** | | | | |
| 5.1 | **NI** | “The trial was retrospectively registered at Clinicaltrials.gov (ChiCTR2100050660).” The cited identifier corresponds to a multicenter three-arm trial with a different stimulation protocol; no separate protocol or statistical analysis plan was provided. | Cai et al., p. 5166; Patients and Methods | Registration was retrospective and the cited record does not correspond to the design reported here, so pre-specification of the analysis could not be verified. |
| 5.2 | **PN** | “The primary outcome was first flatus time. Secondary outcomes included first oral feeding time, bowel sound recovery time and length of hospitalization after surgery.” All four measures were reported in Table 2. | Cai et al., pp. 5168-5169; Outcomes and Table 2 | The prespecified measures were reported, with no evidence of selection among multiple eligible measurements within this outcome domain. |
| 5.3 | **PN** | “Non-normal distribution metric data are represented by median and interquartile range (M(IQR)). The Wilcoxon rank sum test was used for intergroup comparison.” | Cai et al., p. 5168; Statistical Analysis | A single prespecified comparison was described for each continuous outcome. |
| **D5 judgment: Some concerns** | | | | |
| **Overall risk-of-bias judgment: Some concerns. Randomization was concealed and outcome assessors were masked. Concerns arose because three randomized participants were excluded on the basis of treatment received, because blinding success was not assessed for a participant-reported outcome in which only one arm received electrical stimulation, and because the trial was registered retrospectively under an identifier corresponding to a different trial design.** | | | | |

**Cai et al. (2025)**

**Outcome: Length of hospital stay**

*Assessment target: length of postoperative hospitalization in the electroacupuncture-versus-sham-electroacupuncture comparison, reported as median (interquartile range) [5 (4-6) vs 5 (5-6) days]. Means and standard deviations were estimated from the reported medians and interquartile ranges using the method of Wan et al. before quantitative synthesis.*

| **Domain / SQ** | **Response** | **Supporting quotation / source evidence** | **Location** | **Rationale** |
| --- | --- | --- | --- | --- |
| 1.1 | **Y** | “The random number sequence was generated by an independent statistician who did not participate in any other part of the study,” and patients were “randomly allocated into acupuncture or sham acupuncture group in a ratio of 1:1 by block randomization.” | Cai et al., p. 5166; Randomization and Blinding | An independent statistician generated a block-randomized allocation sequence. |
| 1.2 | **Y** | “The sequential number was sealed into opaque envelopes and kept by the project coordinator.” | Cai et al., p. 5166; Randomization and Blinding | Sequentially numbered, sealed opaque envelopes held by an independent coordinator provided allocation concealment. |
| 1.3 | **N** | “There was no statistical difference in baseline characteristics between the groups (P > 0.05).” | Cai et al., p. 5168; Patient Characteristics and Table 1 | Baseline data did not suggest a problem with the randomization process. |
| **D1 judgment: Low risk** | | | | |
| 2.1 | **PN** | Sham electroacupuncture used identical needling without current, and “The patients had an eye patch placed over their eyes to achieve blinding.” | Cai et al., pp. 5166-5167; Randomization and Blinding/Interventions | Both groups received needle insertion and visual cues were masked, so participants were probably unaware of assignment; blinding success was not formally assessed. |
| 2.2 | **Y** | “Acupuncturists could not be blinded owing to the acupuncture procedure.” | Cai et al., p. 5166; Randomization and Blinding | The practitioners delivering the intervention necessarily knew the assigned procedure. |
| 2.3 | **NI** | The flow diagram records one discontinuation in the electroacupuncture arm and two in the sham arm, and the Results attribute three exclusions to “incomplete treatment,” without explaining why treatment was incomplete. | Cai et al., pp. 5167-5168; Figure 1 and Patient Characteristics | Post-randomization departures occurred, but their relationship to the trial context could not be determined. |
| 2.4 | **NA** | Not applicable based on the preceding signaling-question response. | - | The question was not reached in the RoB 2 pathway. |
| 2.5 | **NA** | Not applicable based on the preceding signaling-question response. | - | The question was not reached in the RoB 2 pathway. |
| 2.6 | **N** | “In total, 105 patients met the inclusion criteria. Of them, seven were excluded because of widespread tumor metastasis (n = 3), refusal of acupuncture therapy (n = 1), and incomplete treatment (n = 3).” The flow diagram shows 101 randomized (51 and 50) and 98 analyzed (50 and 48). | Cai et al., pp. 5167-5168; Figure 1 and Patient Characteristics | Three randomized participants were excluded because they did not complete the assigned treatment. Exclusion on the basis of treatment received corresponds to a per-protocol analysis rather than an estimate of the effect of assignment. |
| 2.7 | **PN** | Three of 101 randomized participants (one electroacupuncture and two sham) were excluded, corresponding to approximately 3% of the randomized sample. | Cai et al., p. 5167; Figure 1 | The exclusions were few and approximately balanced between groups, so a substantial impact on the result was unlikely. |
| **D2 judgment: Some concerns** | | | | |
| 3.1 | **Y** | Ninety-eight of 101 randomized participants were included in the analysis (50 and 48). | Cai et al., p. 5167; Figure 1 | Outcome data were available for 97% of randomized participants, considered nearly all. |
| 3.2 | **NA** | Not applicable based on the preceding signaling-question response. | - | The question was not reached in the RoB 2 pathway. |
| 3.3 | **NA** | Not applicable based on the preceding signaling-question response. | - | The question was not reached in the RoB 2 pathway. |
| 3.4 | **NA** | Not applicable based on the preceding signaling-question response. | - | The question was not reached in the RoB 2 pathway. |
| **D3 judgment: Low risk** | | | | |
| 4.1 | **N** | Secondary outcomes included “Length of postoperative hospitalization”, reported in Table 2 as 5 (4-6) vs 5 (5-6) days. | Cai et al., pp. 5168-5169; Outcomes and Table 2 | Length of postoperative hospitalization is an appropriate postoperative recovery measure. |
| 4.2 | **N** | All participants were managed under the same ERAS pathway, which prescribed early postoperative activity and eating and standardized analgesic and pro-motility drug use. | Cai et al., p. 5167; Interventions | A common perioperative pathway was applied to both groups. |
| 4.3 | **NI** | “The assessors of outcome measures and the statisticians performing the data analysis were blinded”; the report does not state whether the clinicians who determined the timing of oral feeding and discharge were blinded. | Cai et al., pp. 5166-5167; Randomization and Blinding | This outcome depended in part on clinical decisions made by the treating team, whose masking was not reported. |
| 4.4 | **Y** | Length of postoperative hospitalization was determined in part by clinical decisions rather than by a participant-reported event alone. | Cai et al., pp. 5167-5168; Interventions and Outcomes | Knowledge of allocation could influence the timing of these clinical decisions. |
| 4.5 | **PN** | Discharge occurred within the standardized ERAS pathway and the reported medians were identical in the two groups (5 days). | Cai et al., pp. 5167 and 5169; Interventions and Table 2 | Influence was possible, but was not shown to be likely under the common perioperative pathway. |
| **D4 judgment: Some concerns** | | | | |
| 5.1 | **NI** | “The trial was retrospectively registered at Clinicaltrials.gov (ChiCTR2100050660).” The cited identifier corresponds to a multicenter three-arm trial with a different stimulation protocol; no separate protocol or statistical analysis plan was provided. | Cai et al., p. 5166; Patients and Methods | Registration was retrospective and the cited record does not correspond to the design reported here, so pre-specification of the analysis could not be verified. |
| 5.2 | **PN** | “The primary outcome was first flatus time. Secondary outcomes included first oral feeding time, bowel sound recovery time and length of hospitalization after surgery.” All four measures were reported in Table 2. | Cai et al., pp. 5168-5169; Outcomes and Table 2 | The prespecified measures were reported, with no evidence of selection among multiple eligible measurements within this outcome domain. |
| 5.3 | **PN** | “Non-normal distribution metric data are represented by median and interquartile range (M(IQR)). The Wilcoxon rank sum test was used for intergroup comparison.” | Cai et al., p. 5168; Statistical Analysis | A single prespecified comparison was described for each continuous outcome. |
| **D5 judgment: Some concerns** | | | | |
| **Overall risk-of-bias judgment: Some concerns. Randomization was concealed and outcome assessors were masked. Concerns arose because three randomized participants were excluded on the basis of treatment received, because the masking of the clinicians who determined discharge was not reported for this partly clinician-dependent outcome, and because the trial was registered retrospectively under an identifier corresponding to a different trial design.** | | | | |

**Ding and Huang (2021)**

**Outcome: Time to first flatus**

*Assessment target: time to first flatus. Judgments relied primarily on the detailed Chinese Methods/Results because the English abstract and several tables contained internal inconsistencies.*

| **Domain / SQ** | **Response** | **Supporting quotation / source evidence** | **Location** | **Rationale** |
| --- | --- | --- | --- | --- |
| 1.1 | **PY** | The Chinese Methods reported block randomization, but the English abstract described allocation “on the basis of whether they opted to receive” treatment, and Section 1.3 incorrectly referred to three groups of 30. | Ding and Huang, pp. 107-109; Abstract and Section 1.3 | The detailed Chinese Methods support randomization, but major internal inconsistencies prevent an unequivocal “Yes.” |
| 1.2 | **NI** | The Chinese Methods describe randomization cards that were centrally distributed and managed and were opened only after a patient had been enrolled, but do not state that the cards were sealed, opaque or sequentially numbered, and no further concealment procedure is described. | Ding and Huang, p. 109; Section 1.3 | The described procedure is compatible with allocation concealment, but the report does not provide sufficient detail to verify that the sequence was concealed until enrollment and assignment, particularly given the internal inconsistencies elsewhere in the description of the randomization process. |
| 1.3 | **PN** | The Results reported two groups of 30 with no significant baseline differences, but Table 1 incorrectly listed the total sample as 93 and contained percentage errors. | Ding and Huang, p. 109; Section 2.1 and Table 1 | No clear baseline imbalance indicated failed randomization, but substantial reporting errors reduced confidence. |
| **D1 judgment: Some concerns** | | | | |
| 2.1 | **PN** | The report stated that sham acupuncture was used to blind patients, but blinding success was not tested. | Ding and Huang, p. 109; Sections 1.2.2-1.3 | Participants were probably blinded using nonpenetrating sham stimulation and inactive leads. |
| 2.2 | **Y** | “由实施电针操作的中医科医生负责揭开随机卡片组别…实现对外科治疗医生设盲” [The acupuncture physician opened the card; surgical doctors were blinded.] | Ding and Huang, p. 109; Section 1.3 | Acupuncture providers knew allocation, while surgical clinicians were blinded. |
| 2.3 | **PN** | “研究纳入60人…无脱落及严重不良反应。” [Sixty participants were included; there were no dropouts or serious adverse events.] | Ding and Huang, p. 109; Section 2.1 | No important trial-context deviation, crossover, or imbalance in co-interventions was reported, although reporting was concise. |
| 2.4 | **NA** | Not applicable based on the preceding signaling-question response. | - | The question was not reached in the RoB 2 pathway. |
| 2.5 | **NA** | Not applicable based on the preceding signaling-question response. | - | The question was not reached in the RoB 2 pathway. |
| 2.6 | **Y** | “研究纳入60人，随机分为2组，每组各30人。无脱落…” [All 60 randomized participants, 30 per group, were retained.] | Ding and Huang, p. 109; Section 2.1 | All randomized participants were included in the reported analyses. |
| 2.7 | **NA** | Not applicable based on the preceding signaling-question response. | - | The question was not reached in the RoB 2 pathway. |
| **D2 judgment: Low risk** | | | | |
| 3.1 | **Y** | “研究纳入60人…无脱落” [All 60 participants were included; there were no dropouts.] | Ding and Huang, p. 109; Section 2.1 | Outcome data were available for all randomized participants. |
| 3.2 | **NA** | Not applicable based on the preceding signaling-question response. | - | The question was not reached in the RoB 2 pathway. |
| 3.3 | **NA** | Not applicable based on the preceding signaling-question response. | - | The question was not reached in the RoB 2 pathway. |
| 3.4 | **NA** | Not applicable based on the preceding signaling-question response. | - | The question was not reached in the RoB 2 pathway. |
| **D3 judgment: Low risk** | | | | |
| 4.1 | **N** | “观察指标：术后首次排气时间…术后首次排便时间…” [Observed outcomes included postoperative time to first flatus and first defecation.] | Ding and Huang, p. 109; Section 1.5 | The measures were appropriate conventional recovery outcomes. |
| 4.2 | **N** | The same outcome definitions and follow-up period were used in both groups. | Ding and Huang, Sections 1.5 and 2.3, pp. 109-110 | No differential measurement method was reported. |
| 4.3 | **PN** | The report stated that patients and surgical clinicians were blinded; the acupuncture practitioner opened the allocation card. | Ding and Huang, p. 109; Section 1.3 | Outcome assessors were probably blinded, although blinding success and assessor identity were not independently verified. |
| 4.4 | **NA** | Not applicable based on the preceding signaling-question response. | - | The question was not reached in the RoB 2 pathway. |
| 4.5 | **NA** | Not applicable based on the preceding signaling-question response. | - | The question was not reached in the RoB 2 pathway. |
| **D4 judgment: Low risk** | | | | |
| 5.1 | **NI** | No registry record, protocol, or statistical analysis plan was reported. The article described this sample as 60 distal-gastrectomy patients selected from a broader RCT of gastric and periampullary tumors. | Ding and Huang, pp. 108-109; Section 1.1 | The prespecification of this surgical subgroup, outcomes, and analyses before results were known could not be verified. |
| 5.2 | **PN** | The Methods prespecified time to first flatus and first defecation as observation indicators. | Ding and Huang, p. 109; Section 1.5 | No evidence indicated selection from multiple eligible measurements within either outcome domain. |
| 5.3 | **PN** | The Methods describe a single set of t tests for continuous outcomes. No protocol or statistical analysis plan confirming that this subgroup report was prespecified within the larger trial was available. | Ding and Huang, pp. 108-109; Sections 1.1 and 1.4 | No prespecified analysis plan could be located, and this report describes a surgical subgroup of a larger trial. However, only one straightforward between-group comparison was described for this outcome and no indication of result-driven selection among multiple eligible analyses was identified. In line with the RoB 2 guidance for this situation, “probably no” was used in preference to “no information”. |
| **D5 judgment: Some concerns** | | | | |
| **Overall risk-of-bias judgment: Some concerns. Concerns arose from internally inconsistent randomization reporting and unverifiable prespecification of a subgroup report from a larger RCT; no domain was judged high risk.** | | | | |

**Ding and Huang (2021)**

**Outcome: Time to first defecation**

*Assessment target: time to first defecation. Judgments relied primarily on the detailed Chinese Methods/Results because the English abstract and several tables contained internal inconsistencies.*

| **Domain / SQ** | **Response** | **Supporting quotation / source evidence** | **Location** | **Rationale** |
| --- | --- | --- | --- | --- |
| 1.1 | **PY** | The Chinese Methods reported block randomization, but the English abstract described allocation “on the basis of whether they opted to receive” treatment, and Section 1.3 incorrectly referred to three groups of 30. | Ding and Huang, pp. 107-109; Abstract and Section 1.3 | The detailed Chinese Methods support randomization, but major internal inconsistencies prevent an unequivocal “Yes.” |
| 1.2 | **NI** | The Chinese Methods describe randomization cards that were centrally distributed and managed and were opened only after a patient had been enrolled, but do not state that the cards were sealed, opaque or sequentially numbered, and no further concealment procedure is described. | Ding and Huang, p. 109; Section 1.3 | The described procedure is compatible with allocation concealment, but the report does not provide sufficient detail to verify that the sequence was concealed until enrollment and assignment, particularly given the internal inconsistencies elsewhere in the description of the randomization process. |
| 1.3 | **PN** | The Results reported two groups of 30 with no significant baseline differences, but Table 1 incorrectly listed the total sample as 93 and contained percentage errors. | Ding and Huang, p. 109; Section 2.1 and Table 1 | No clear baseline imbalance indicated failed randomization, but substantial reporting errors reduced confidence. |
| **D1 judgment: Some concerns** | | | | |
| 2.1 | **PN** | The report stated that sham acupuncture was used to blind patients, but blinding success was not tested. | Ding and Huang, p. 109; Sections 1.2.2-1.3 | Participants were probably blinded using nonpenetrating sham stimulation and inactive leads. |
| 2.2 | **Y** | “由实施电针操作的中医科医生负责揭开随机卡片组别…实现对外科治疗医生设盲” [The acupuncture physician opened the card; surgical doctors were blinded.] | Ding and Huang, p. 109; Section 1.3 | Acupuncture providers knew allocation, while surgical clinicians were blinded. |
| 2.3 | **PN** | “研究纳入60人…无脱落及严重不良反应。” [Sixty participants were included; there were no dropouts or serious adverse events.] | Ding and Huang, p. 109; Section 2.1 | No important trial-context deviation, crossover, or imbalance in co-interventions was reported, although reporting was concise. |
| 2.4 | **NA** | Not applicable based on the preceding signaling-question response. | - | The question was not reached in the RoB 2 pathway. |
| 2.5 | **NA** | Not applicable based on the preceding signaling-question response. | - | The question was not reached in the RoB 2 pathway. |
| 2.6 | **Y** | “研究纳入60人，随机分为2组，每组各30人。无脱落…” [All 60 randomized participants, 30 per group, were retained.] | Ding and Huang, p. 109; Section 2.1 | All randomized participants were included in the reported analyses. |
| 2.7 | **NA** | Not applicable based on the preceding signaling-question response. | - | The question was not reached in the RoB 2 pathway. |
| **D2 judgment: Low risk** | | | | |
| 3.1 | **Y** | “研究纳入60人…无脱落” [All 60 participants were included; there were no dropouts.] | Ding and Huang, p. 109; Section 2.1 | Outcome data were available for all randomized participants. |
| 3.2 | **NA** | Not applicable based on the preceding signaling-question response. | - | The question was not reached in the RoB 2 pathway. |
| 3.3 | **NA** | Not applicable based on the preceding signaling-question response. | - | The question was not reached in the RoB 2 pathway. |
| 3.4 | **NA** | Not applicable based on the preceding signaling-question response. | - | The question was not reached in the RoB 2 pathway. |
| **D3 judgment: Low risk** | | | | |
| 4.1 | **N** | “观察指标：术后首次排气时间…术后首次排便时间…” [Observed outcomes included postoperative time to first flatus and first defecation.] | Ding and Huang, p. 109; Section 1.5 | The measures were appropriate conventional recovery outcomes. |
| 4.2 | **N** | The same outcome definitions and follow-up period were used in both groups. | Ding and Huang, Sections 1.5 and 2.3, pp. 109-110 | No differential measurement method was reported. |
| 4.3 | **PN** | The report stated that patients and surgical clinicians were blinded; the acupuncture practitioner opened the allocation card. | Ding and Huang, p. 109; Section 1.3 | Outcome assessors were probably blinded, although blinding success and assessor identity were not independently verified. |
| 4.4 | **NA** | Not applicable based on the preceding signaling-question response. | - | The question was not reached in the RoB 2 pathway. |
| 4.5 | **NA** | Not applicable based on the preceding signaling-question response. | - | The question was not reached in the RoB 2 pathway. |
| **D4 judgment: Low risk** | | | | |
| 5.1 | **NI** | No registry record, protocol, or statistical analysis plan was reported. The article described this sample as 60 distal-gastrectomy patients selected from a broader RCT of gastric and periampullary tumors. | Ding and Huang, pp. 108-109; Section 1.1 | The prespecification of this surgical subgroup, outcomes, and analyses before results were known could not be verified. |
| 5.2 | **PN** | The Methods prespecified time to first flatus and first defecation as observation indicators. | Ding and Huang, p. 109; Section 1.5 | No evidence indicated selection from multiple eligible measurements within either outcome domain. |
| 5.3 | **PN** | The Methods describe a single set of t tests for continuous outcomes. No protocol or statistical analysis plan confirming that this subgroup report was prespecified within the larger trial was available. | Ding and Huang, pp. 108-109; Sections 1.1 and 1.4 | No prespecified analysis plan could be located, and this report describes a surgical subgroup of a larger trial. However, only one straightforward between-group comparison was described for this outcome and no indication of result-driven selection among multiple eligible analyses was identified. In line with the RoB 2 guidance for this situation, “probably no” was used in preference to “no information”. |
| **D5 judgment: Some concerns** | | | | |
| **Overall risk-of-bias judgment: Some concerns. Concerns arose from internally inconsistent randomization reporting and unverifiable prespecification of a subgroup report from a larger RCT; no domain was judged high risk.** | | | | |

**Ding and Huang (2021)**

**Outcome: Length of hospital stay**

*Assessment target: length of hospital stay. Judgments relied primarily on the detailed Chinese Methods/Results because the English abstract and several tables contained internal inconsistencies. The trial reported “hospital stay (days)” without specifying whether this represented total or postoperative stay: the Methods list this item without the qualifier “postoperative” used for the adjacent outcomes, whereas the Discussion refers to postoperative hospital stay. The reported hospital-stay measure was retained for synthesis, with this ambiguity acknowledged.*

| **Domain / SQ** | **Response** | **Supporting quotation / source evidence** | **Location** | **Rationale** |
| --- | --- | --- | --- | --- |
| 1.1 | **PY** | The Chinese Methods reported block randomization, but the English abstract described allocation “on the basis of whether they opted to receive” treatment, and Section 1.3 incorrectly referred to three groups of 30. | Ding and Huang, pp. 107-109; Abstract and Section 1.3 | The detailed Chinese Methods support randomization, but major internal inconsistencies prevent an unequivocal “Yes.” |
| 1.2 | **NI** | The Chinese Methods describe randomization cards that were centrally distributed and managed and were opened only after a patient had been enrolled, but do not state that the cards were sealed, opaque or sequentially numbered, and no further concealment procedure is described. | Ding and Huang, p. 109; Section 1.3 | The described procedure is compatible with allocation concealment, but the report does not provide sufficient detail to verify that the sequence was concealed until enrollment and assignment, particularly given the internal inconsistencies elsewhere in the description of the randomization process. |
| 1.3 | **PN** | The Results reported two groups of 30 with no significant baseline differences, but Table 1 incorrectly listed the total sample as 93 and contained percentage errors. | Ding and Huang, p. 109; Section 2.1 and Table 1 | No clear baseline imbalance indicated failed randomization, but substantial reporting errors reduced confidence. |
| **D1 judgment: Some concerns** | | | | |
| 2.1 | **PN** | The report stated that sham acupuncture was used to blind patients, but blinding success was not tested. | Ding and Huang, p. 109; Sections 1.2.2-1.3 | Participants were probably blinded using nonpenetrating sham stimulation and inactive leads. |
| 2.2 | **Y** | “由实施电针操作的中医科医生负责揭开随机卡片组别…实现对外科治疗医生设盲” [The acupuncture physician opened the card; surgical doctors were blinded.] | Ding and Huang, p. 109; Section 1.3 | Acupuncture providers knew allocation, while surgical clinicians were blinded. |
| 2.3 | **PN** | “研究纳入60人…无脱落及严重不良反应。” [Sixty participants were included; there were no dropouts or serious adverse events.] | Ding and Huang, p. 109; Section 2.1 | No important trial-context deviation, crossover, or imbalance in co-interventions was reported, although reporting was concise. |
| 2.4 | **NA** | Not applicable based on the preceding signaling-question response. | - | The question was not reached in the RoB 2 pathway. |
| 2.5 | **NA** | Not applicable based on the preceding signaling-question response. | - | The question was not reached in the RoB 2 pathway. |
| 2.6 | **Y** | “研究纳入60人，随机分为2组，每组各30人。无脱落…” [All 60 randomized participants, 30 per group, were retained.] | Ding and Huang, p. 109; Section 2.1 | All randomized participants were included in the reported analyses. |
| 2.7 | **NA** | Not applicable based on the preceding signaling-question response. | - | The question was not reached in the RoB 2 pathway. |
| **D2 judgment: Low risk** | | | | |
| 3.1 | **Y** | “研究纳入60人…无脱落” [All 60 participants were included; there were no dropouts.] | Ding and Huang, p. 109; Section 2.1 | Outcome data were available for all randomized participants. |
| 3.2 | **NA** | Not applicable based on the preceding signaling-question response. | - | The question was not reached in the RoB 2 pathway. |
| 3.3 | **NA** | Not applicable based on the preceding signaling-question response. | - | The question was not reached in the RoB 2 pathway. |
| 3.4 | **NA** | Not applicable based on the preceding signaling-question response. | - | The question was not reached in the RoB 2 pathway. |
| **D3 judgment: Low risk** | | | | |
| 4.1 | **N** | “住院时间（d）” [hospital stay (days)] was prespecified as an observation indicator. | Ding and Huang, p. 109; Section 1.5 | Hospital stay in days is an appropriate outcome measure. |
| 4.2 | **N** | The same outcome definition and postoperative follow-up were used in both groups. | Ding and Huang, Sections 1.5 and 2.3, pp. 109-110 | No group-specific ascertainment method was reported. |
| 4.3 | **PN** | The report stated that patients and surgical clinicians were blinded; the acupuncture practitioner opened the allocation card. | Ding and Huang, p. 109; Section 1.3 | Outcome assessors were probably blinded, although blinding success and assessor identity were not independently verified. |
| 4.4 | **NA** | Not applicable based on the preceding signaling-question response. | - | The question was not reached in the RoB 2 pathway. |
| 4.5 | **NA** | Not applicable based on the preceding signaling-question response. | - | The question was not reached in the RoB 2 pathway. |
| **D4 judgment: Low risk** | | | | |
| 5.1 | **NI** | No registry record, protocol, or statistical analysis plan was reported. The article described this sample as 60 distal-gastrectomy patients selected from a broader RCT of gastric and periampullary tumors. | Ding and Huang, pp. 108-109; Section 1.1 | The prespecification of this surgical subgroup, outcomes, and analyses before results were known could not be verified. |
| 5.2 | **PN** | “住院时间（d）” [hospital stay (days)] was explicitly prespecified in the Methods and reported in Table 3. | Ding and Huang, pp. 109-110; Sections 1.5 and 2.3/Table 3 | No evidence indicated selection from multiple eligible measurements within the LOS domain. |
| 5.3 | **PN** | The Methods describe a single set of t tests for continuous outcomes. No protocol or statistical analysis plan confirming that this subgroup report was prespecified within the larger trial was available. | Ding and Huang, pp. 108-109; Sections 1.1 and 1.4 | No prespecified analysis plan could be located, and this report describes a surgical subgroup of a larger trial. However, only one straightforward between-group comparison was described for this outcome and no indication of result-driven selection among multiple eligible analyses was identified. In line with the RoB 2 guidance for this situation, “probably no” was used in preference to “no information”. |
| **D5 judgment: Some concerns** | | | | |
| **Overall risk-of-bias judgment: Some concerns. Concerns arose from internally inconsistent randomization reporting and unverifiable prespecification of a subgroup report from a larger RCT; no domain was judged high risk.** | | | | |

**Wang et al. (2026)**

**Outcomes: Time to first flatus and time to first defecation**

*Assessment target: transcutaneous acupoint electrical stimulation plus parenteral nutrition versus parenteral nutrition alone (n=45 vs n=45).*

| **Domain / SQ** | **Response** | **Supporting quotation / source evidence** | **Location** | **Rationale** |
| --- | --- | --- | --- | --- |
| 1.1 | **Y** | Ninety patients were allocated to two groups of 45 using a random number table (随机数字表法). | Methods, 1.1 | An adequate random sequence-generation method is described. |
| 1.2 | **NI** | No allocation-concealment procedure is described. | Methods, 1.1 | Concealment before assignment could not be verified. |
| 1.3 | **PN** | Sex, age, body mass index, tumor stage, and extent of resection are reported as not significantly different between groups. | Methods, 1.1 | No important baseline imbalance suggesting failed randomization was identified. |
| **D1 judgment: Some concerns. Allocation concealment was not reported.** | | | | |
| 2.1 | **Y** | The control group received parenteral nutrition alone; no sham stimulation was applied. | Methods, 1.2 | Participants necessarily knew whether stimulation was delivered. |
| 2.2 | **Y** | A practitioner explained and delivered the stimulation procedure. | Methods, 1.2 | Intervention providers knew the assignment. |
| 2.3 | **PN** | Both groups received the same routine postoperative care and the same parenteral nutrition regimen; no assignment-related deviations are reported. | Methods, 1.2 | Co-interventions were specified and applied comparably. |
| 2.6 | **Y** | All 45 randomized participants per group appear in the outcome analyses. | Results, Table 1 | The analysis corresponds to the randomized groups. |
| **D2 judgment: Low risk. The open-label design was not accompanied by documented trial-context deviations and all randomized participants were analyzed.** | | | | |
| 3.1 | **Y** | No withdrawals or losses are reported and all randomized participants appear in the analysis. | Results, Table 1 | No missing outcome data were identified. |
| **D3 judgment: Low risk. Outcome data were available for all randomized participants.** | | | | |
| 4.1 | **N** | Times to recovery of bowel sounds, first flatus, and first defecation were prespecified as gastrointestinal-function indicators and reported in hours. | Methods, 1.3.1; Table 1 | The measures are standard recovery milestones for this field. |
| 4.2 | **N** | The same postoperative gastrointestinal-function indicators were recorded in both groups, with no group-specific measurement procedure described. | Methods, 1.3.1 | The measurement/ascertainment procedure was intended to be the same across groups. |
| 4.3 | **Y** | No sham was used; participants were necessarily aware of assignment, and the report does not describe blinded ascertainment or identify a blinded assessor for first flatus or defecation. | Methods, 1.2 and 1.3.1 | For participant-dependent recovery events, participant knowledge constitutes knowledge at the point of outcome ascertainment. |
| 4.4 | **Y** | First flatus and defecation depend on recognition and reporting of events. | Methods, 1.3.1 | Expectation or differential reporting could influence the recorded timing. |
| 4.5 | **PY** | The comparison was open-label with no reported masking of outcome ascertainment. | Methods | Consistent with the approach applied to other open-label, report-dependent recovery outcomes in this review. |
| **D4 judgment: High risk. Unblinded assessment of participant-dependent recovery milestones.** | | | | |
| 5.1 | **NI** | No trial registration number, protocol, or statistical analysis plan is cited; ethics approval 2022-042-2 is reported. | Methods, 1.1 | A prespecified analysis plan could not be verified. |
| 5.2 | **PN** | The three gastrointestinal-function indicators defined in the Methods are reported. | Methods, 1.3.1 and Table 1 | No evidence of selective choice among multiple eligible measurements was identified. |
| 5.3 | **PN** | No alternative eligible analyses of the timing outcomes are described. | Methods and Results | No evidence of selective analysis was identified. |
| **D5 judgment: Some concerns. Prospective prespecification could not be verified.** | | | | |
| **Overall risk-of-bias judgment: High risk. Driven by Domain 4 for both outcomes.** | | | | |

**Huang and Li (2023)**

**Outcome: Time to first flatus**

*Assessment target: transcutaneous electrical acupoint stimulation added to fast-track nursing versus fast-track nursing alone; time to first flatus.*

| **Domain / SQ** | **Response** | **Supporting quotation / source evidence** | **Location** | **Rationale** |
| --- | --- | --- | --- | --- |
| 1.1 | **Y** | Fifty-two enrolled patients were divided into two groups of 26 using a random number table (随机数字表法). | Methods, 1.1 | An adequate random sequence-generation method is described. |
| 1.2 | **NI** | No allocation-concealment procedure is described; the report states only that enrolled patients were divided using a random number table. | Methods, 1.1 | Concealment before assignment could not be verified. |
| 1.3 | **PN** | Sex, age and disease duration are reported for both groups, with no significant difference in general characteristics. | Methods, 1.1 | No baseline imbalance suggesting failed randomization was identified, although the reported baseline characteristics are limited. |
| **D1 judgment: Some concerns. Allocation concealment was not reported.** | | | | |
| 2.1 | **Y** | The control group received fast-track nursing alone and the study group received the same nursing with transcutaneous electrical acupoint stimulation added (试验组在对照组基础上增加经皮穴位电刺激); no sham device was used. | Methods, 1.2 | Participants necessarily knew whether stimulation was delivered. |
| 2.2 | **Y** | Electrodes were applied and the stimulator (G6805-2a) was operated at 10 Hz and 20 mA, with the intensity adjusted to the individual patient. | Methods, 1.2 | Intervention providers knew the assignment. |
| 2.3 | **PN** | Both groups received the identical fast-track nursing program and the study group differed only by the addition of stimulation; no crossover, rescue treatment or imbalance in co-interventions is reported. | Methods, 1.2 | Co-interventions were specified and applied comparably. |
| 2.6 | **Y** | All 26 randomized participants per group appear in the postoperative recovery analyses. | Results, Table 2 | The analysis corresponds to the randomized groups. |
| **D2 judgment: Low risk. The absence of a sham was not accompanied by documented trial-context deviations or imbalanced co-interventions.** | | | | |
| 3.1 | **Y** | The exclusion criteria included participants unable to cooperate with the study or withdrawing during the study (无法配合本次研究或中途退出研究者). The report nevertheless states that 52 enrolled participants were randomized 26 to each group, and both randomized group sizes are retained in the outcome table. | Methods, 1.1; Results, Table 2 | No post-randomization loss or missing outcome data were reported. The wording of the exclusion criterion is ambiguous and no participant flow diagram is provided, but the reported randomized and analyzed group sizes are identical. |
| **D3 judgment: Low risk. Outcome data were available for all randomized participants as reported.** | | | | |
| 4.1 | **N** | Time to first flatus was prespecified among the postoperative recovery indicators (术后排气时间) and reported in days. | Methods, 1.3.2; Results, Table 2 | The measure is a standard recovery milestone for this field. |
| 4.2 | **N** | The same postoperative recovery indicators were recorded in both groups, with no group-specific procedure described. | Methods, 1.3.2 | The measurement procedure was intended to be the same across groups. |
| 4.3 | **Y** | No sham was used; participants and clinicians were necessarily aware of assignment, and the report does not describe any blinded outcome ascertainment. | Methods, 1.2 and 1.3.2 | Those assessing the outcome were aware of the assigned intervention. |
| 4.4 | **Y** | First flatus depends on recognition and reporting of the event by the participant. | Methods, 1.3.2 | Knowledge of assignment could influence the recorded value. |
| 4.5 | **PY** | The comparison was open-label with no reported masking of outcome ascertainment, and the milestone is participant-dependent. | Methods and Results | The comparison was open-label with no reported masking of outcome ascertainment, and the milestone is participant-dependent. |
| **D4 judgment: High risk. Unblinded assessment of a participant-dependent recovery milestone.** | | | | |
| 5.1 | **NI** | No trial registration number, protocol, or statistical analysis plan is cited; the report states only that the study was approved by the hospital ethics committee. | Methods, 1.1 | A prespecified analysis plan could not be verified. |
| 5.2 | **PN** | The postoperative recovery indicators defined in the Methods (time to first flatus, first feeding, urinary catheter duration and length of stay) are all reported. | Methods, 1.3.2; Results, Table 2 | No evidence of selective choice among multiple eligible measurements was identified. |
| 5.3 | **PN** | The statistical section specifies the t test for continuous variables, and no alternative eligible analyses are described. | Methods, 1.4; Results | No evidence of selective analysis was identified. |
| **D5 judgment: Some concerns. Prospective prespecification could not be verified.** | | | | |
| **Overall risk-of-bias judgment: High risk. Driven by Domain 4; Domains 1 and 5 raise some concerns.** | | | | |

**Huang and Li (2023)**

**Outcome: Length of hospital stay**

*Assessment target: transcutaneous electrical acupoint stimulation added to fast-track nursing versus fast-track nursing alone; length of hospital stay.*

| **Domain / SQ** | **Response** | **Supporting quotation / source evidence** | **Location** | **Rationale** |
| --- | --- | --- | --- | --- |
| 1.1 | **Y** | Fifty-two enrolled patients were divided into two groups of 26 using a random number table (随机数字表法). | Methods, 1.1 | An adequate random sequence-generation method is described. |
| 1.2 | **NI** | No allocation-concealment procedure is described; the report states only that enrolled patients were divided using a random number table. | Methods, 1.1 | Concealment before assignment could not be verified. |
| 1.3 | **PN** | Sex, age and disease duration are reported for both groups, with no significant difference in general characteristics. | Methods, 1.1 | No baseline imbalance suggesting failed randomization was identified, although the reported baseline characteristics are limited. |
| **D1 judgment: Some concerns. Allocation concealment was not reported.** | | | | |
| 2.1 | **Y** | The control group received fast-track nursing alone and the study group received the same nursing with transcutaneous electrical acupoint stimulation added (试验组在对照组基础上增加经皮穴位电刺激); no sham device was used. | Methods, 1.2 | Participants necessarily knew whether stimulation was delivered. |
| 2.2 | **Y** | Electrodes were applied and the stimulator (G6805-2a) was operated at 10 Hz and 20 mA, with the intensity adjusted to the individual patient. | Methods, 1.2 | Intervention providers knew the assignment. |
| 2.3 | **PN** | Both groups received the identical fast-track nursing program and the study group differed only by the addition of stimulation; no crossover, rescue treatment or imbalance in co-interventions is reported. | Methods, 1.2 | Co-interventions were specified and applied comparably. |
| 2.6 | **Y** | All 26 randomized participants per group appear in the postoperative recovery analyses. | Results, Table 2 | The analysis corresponds to the randomized groups. |
| **D2 judgment: Low risk. The absence of a sham was not accompanied by documented trial-context deviations or imbalanced co-interventions.** | | | | |
| 3.1 | **Y** | The exclusion criteria included participants unable to cooperate with the study or withdrawing during the study (无法配合本次研究或中途退出研究者). The report nevertheless states that 52 enrolled participants were randomized 26 to each group, and both randomized group sizes are retained in the outcome table. | Methods, 1.1; Results, Table 2 | No post-randomization loss or missing outcome data were reported. The wording of the exclusion criterion is ambiguous and no participant flow diagram is provided, but the reported randomized and analyzed group sizes are identical. |
| **D3 judgment: Low risk. Outcome data were available for all randomized participants as reported.** | | | | |
| 4.1 | **N** | Length of hospital stay was prespecified among the postoperative recovery indicators (住院时间) and reported in days. | Methods, 1.3.2; Results, Table 2 | Length of stay is an administratively recorded outcome and a standard recovery measure. |
| 4.2 | **N** | The same postoperative recovery indicators were recorded in both groups, with no group-specific procedure described. | Methods, 1.3.2 | The measurement procedure was intended to be the same across groups. |
| 4.3 | **Y** | No sham was used; participants and clinicians were necessarily aware of assignment, and the report does not describe any blinded outcome ascertainment. | Methods, 1.2 and 1.3.2 | Those assessing the outcome were aware of the assigned intervention. |
| 4.4 | **Y** | Discharge timing is recorded administratively but is influenced by clinical decisions, and no standardized discharge criteria are reported. | Methods, 1.3.2 | Knowledge of assignment could influence the recorded value. |
| 4.5 | **PN** | Although clinicians were aware of assignment and no standardized discharge criteria were reported, there is no indication that discharge decisions were actually influenced by the assignment. | Methods and Results | Although clinicians were aware of assignment and no standardized discharge criteria were reported, there is no indication that discharge decisions were actually influenced by the assignment. |
| **D4 judgment: Some concerns. Discharge decisions were made without standardized criteria by clinicians aware of assignment, but no influence on the recorded outcome was demonstrated.** | | | | |
| 5.1 | **NI** | No trial registration number, protocol, or statistical analysis plan is cited; the report states only that the study was approved by the hospital ethics committee. | Methods, 1.1 | A prespecified analysis plan could not be verified. |
| 5.2 | **PN** | The postoperative recovery indicators defined in the Methods (time to first flatus, first feeding, urinary catheter duration and length of stay) are all reported. | Methods, 1.3.2; Results, Table 2 | No evidence of selective choice among multiple eligible measurements was identified. |
| 5.3 | **PN** | The statistical section specifies the t test for continuous variables, and no alternative eligible analyses are described. | Methods, 1.4; Results | No evidence of selective analysis was identified. |
| **D5 judgment: Some concerns. Prospective prespecification could not be verified.** | | | | |
| **Overall risk-of-bias judgment: Some concerns. Domains 1, 4 and 5 each raise some concerns, without a domain at high risk.** | | | | |

**Shi et al. (2024)**

**Outcome: Time to first flatus**

*Assessment target: true transcutaneous electrical stimulation at PC6, ST36 and LI4 versus the same procedure with the stimulator switched off; time to first flatus.*

| **Domain / SQ** | **Response** | **Supporting quotation / source evidence** | **Location** | **Rationale** |
| --- | --- | --- | --- | --- |
| 1.1 | **Y** | One hundred and eighty patients were divided into a conventional group and an electrical stimulation group of 90 each using a random number table (按随机数字表法). | Methods, 1.4 | An adequate random sequence-generation method is described. |
| 1.2 | **NI** | No allocation-concealment procedure is described. | Methods, 1.4 | Concealment before assignment could not be verified. |
| 1.3 | **PN** | Sex, clinical stage, age and pathological type are reported for both groups, with no significant difference in general characteristics. | Methods, 1.4 | No baseline imbalance suggesting failed randomization was identified; the reported characteristics are reasonably balanced. |
| **D1 judgment: Some concerns. Allocation concealment was not reported.** | | | | |
| 2.1 | **PY** | The stimulation group received 8-12 mA at 2/100 Hz for 30 min every 12 h, whereas the control group underwent the identical procedure with the stimulator not switched on (常规组与电刺激组同样方法但经皮穴位电刺激仪不打开电源). | Methods, 2.1 and 2.2 | The sensory difference between a delivered current of 8-12 mA and no current makes it likely that participants could infer their assignment; no masking procedure or masking check is described. |
| 2.2 | **Y** | The operator applied the electrodes and either switched the stimulator on or left it off. | Methods, 2.1 and 2.2 | Intervention providers knew the assignment. |
| 2.3 | **PN** | Both groups received the same accelerated recovery treatment for gastric cancer (2组均实施胃癌加速康复治疗), and the groups differed only in whether the stimulator was switched on; no crossover, rescue treatment or imbalance in co-interventions is reported. | Methods, 2 | Co-interventions were specified and applied comparably. |
| 2.6 | **Y** | All 90 randomized participants per group appear in the gastrointestinal recovery analyses. | Results, Table 2 | The analysis corresponds to the randomized groups. |
| **D2 judgment: Low risk. Awareness of assignment was not accompanied by documented trial-context deviations or imbalanced co-interventions.** | | | | |
| 3.1 | **Y** | One hundred and eighty elderly patients were randomized 90 to each group, and both groups of 90 appear in the gastrointestinal recovery table; no withdrawals or losses are reported. | Methods, 1.4; Results, Table 2 | No missing outcome data were identified. |
| **D3 judgment: Low risk. Outcome data were available for all randomized participants.** | | | | |
| 4.1 | **N** | Time to first flatus was prespecified among the gastrointestinal recovery indicators (首次肛门排气时间) and reported in hours. | Methods, 3.1; Results, Table 2 | The measure is a standard recovery milestone for this field. |
| 4.2 | **N** | The same recovery indicators were recorded in both groups, with no group-specific procedure described. | Methods, 3.1 | The measurement procedure was intended to be the same across groups. |
| 4.3 | **PY** | The control procedure consisted only of leaving the stimulator switched off, and the report does not state that participants were told a sensation might be absent, does not describe any masking of the device display, does not report a masking check, and does not describe blinded outcome ascertainment. | Methods, 2.2 and 3.1 | The inactive-device comparator is unlikely to have masked assignment, so those assessing the outcome were probably aware of it. This differs from the trial by Gu et al. (2019), in which the lead was severed while the display continued to indicate the set intensity and participants were told in advance that they might feel nothing. |
| 4.4 | **Y** | First flatus depends on recognition and reporting of the event by the participant. | Methods, 3.1 | Expectation or differential reporting could influence the recorded timing. |
| 4.5 | **PY** | A participant-dependent milestone was recorded in a comparison in which the inactive-device control is unlikely to have concealed assignment. | Methods and Results | A participant-dependent milestone was recorded in a comparison in which the inactive-device control is unlikely to have concealed assignment. |
| **D4 judgment: High risk. A participant-dependent milestone was assessed in a comparison whose inactive-device control is unlikely to have masked assignment.** | | | | |
| 5.1 | **NI** | No trial registration number, protocol, or statistical analysis plan is cited; the report cites a provincial research grant only. | Methods, header | A prespecified analysis plan could not be verified. |
| 5.2 | **PN** | The three gastrointestinal recovery indicators defined in the Methods (time to normal bowel sounds, first flatus and first defecation) are all reported. | Methods, 3.1; Results, Table 2 | No evidence of selective choice among multiple eligible measurements was identified. |
| 5.3 | **PN** | The statistical section specifies the independent-samples t test for continuous variables, and no alternative eligible analyses are described. | Methods, 3.2; Results | No evidence of selective analysis was identified. |
| **D5 judgment: Some concerns. Prospective prespecification could not be verified.** | | | | |
| **Overall risk-of-bias judgment: High risk. Driven by Domain 4; Domains 1 and 5 raise some concerns.** | | | | |

**Shi et al. (2024)**

**Outcome: Time to first defecation**

*Assessment target: true transcutaneous electrical stimulation at PC6, ST36 and LI4 versus the same procedure with the stimulator switched off; time to first defecation.*

| **Domain / SQ** | **Response** | **Supporting quotation / source evidence** | **Location** | **Rationale** |
| --- | --- | --- | --- | --- |
| 1.1 | **Y** | One hundred and eighty patients were divided into a conventional group and an electrical stimulation group of 90 each using a random number table (按随机数字表法). | Methods, 1.4 | An adequate random sequence-generation method is described. |
| 1.2 | **NI** | No allocation-concealment procedure is described. | Methods, 1.4 | Concealment before assignment could not be verified. |
| 1.3 | **PN** | Sex, clinical stage, age and pathological type are reported for both groups, with no significant difference in general characteristics. | Methods, 1.4 | No baseline imbalance suggesting failed randomization was identified; the reported characteristics are reasonably balanced. |
| **D1 judgment: Some concerns. Allocation concealment was not reported.** | | | | |
| 2.1 | **PY** | The stimulation group received 8-12 mA at 2/100 Hz for 30 min every 12 h, whereas the control group underwent the identical procedure with the stimulator not switched on (常规组与电刺激组同样方法但经皮穴位电刺激仪不打开电源). | Methods, 2.1 and 2.2 | The sensory difference between a delivered current of 8-12 mA and no current makes it likely that participants could infer their assignment; no masking procedure or masking check is described. |
| 2.2 | **Y** | The operator applied the electrodes and either switched the stimulator on or left it off. | Methods, 2.1 and 2.2 | Intervention providers knew the assignment. |
| 2.3 | **PN** | Both groups received the same accelerated recovery treatment for gastric cancer (2组均实施胃癌加速康复治疗), and the groups differed only in whether the stimulator was switched on; no crossover, rescue treatment or imbalance in co-interventions is reported. | Methods, 2 | Co-interventions were specified and applied comparably. |
| 2.6 | **Y** | All 90 randomized participants per group appear in the gastrointestinal recovery analyses. | Results, Table 2 | The analysis corresponds to the randomized groups. |
| **D2 judgment: Low risk. Awareness of assignment was not accompanied by documented trial-context deviations or imbalanced co-interventions.** | | | | |
| 3.1 | **Y** | One hundred and eighty elderly patients were randomized 90 to each group, and both groups of 90 appear in the gastrointestinal recovery table; no withdrawals or losses are reported. | Methods, 1.4; Results, Table 2 | No missing outcome data were identified. |
| **D3 judgment: Low risk. Outcome data were available for all randomized participants.** | | | | |
| 4.1 | **N** | Time to first defecation was prespecified among the gastrointestinal recovery indicators (首次排便时间) and reported in hours. | Methods, 3.1; Results, Table 2 | The measure is a standard recovery milestone for this field. |
| 4.2 | **N** | The same recovery indicators were recorded in both groups, with no group-specific procedure described. | Methods, 3.1 | The measurement procedure was intended to be the same across groups. |
| 4.3 | **PY** | The control procedure consisted only of leaving the stimulator switched off, and the report does not state that participants were told a sensation might be absent, does not describe any masking of the device display, does not report a masking check, and does not describe blinded outcome ascertainment. | Methods, 2.2 and 3.1 | The inactive-device comparator is unlikely to have masked assignment, so those assessing the outcome were probably aware of it. This differs from the trial by Gu et al. (2019), in which the lead was severed while the display continued to indicate the set intensity and participants were told in advance that they might feel nothing. |
| 4.4 | **Y** | First defecation depends on recognition and reporting of the event by the participant. | Methods, 3.1 | Expectation or differential reporting could influence the recorded timing. |
| 4.5 | **PY** | A participant-dependent milestone was recorded in a comparison in which the inactive-device control is unlikely to have concealed assignment. | Methods and Results | A participant-dependent milestone was recorded in a comparison in which the inactive-device control is unlikely to have concealed assignment. |
| **D4 judgment: High risk. A participant-dependent milestone was assessed in a comparison whose inactive-device control is unlikely to have masked assignment.** | | | | |
| 5.1 | **NI** | No trial registration number, protocol, or statistical analysis plan is cited; the report cites a provincial research grant only. | Methods, header | A prespecified analysis plan could not be verified. |
| 5.2 | **PN** | The three gastrointestinal recovery indicators defined in the Methods (time to normal bowel sounds, first flatus and first defecation) are all reported. | Methods, 3.1; Results, Table 2 | No evidence of selective choice among multiple eligible measurements was identified. |
| 5.3 | **PN** | The statistical section specifies the independent-samples t test for continuous variables, and no alternative eligible analyses are described. | Methods, 3.2; Results | No evidence of selective analysis was identified. |
| **D5 judgment: Some concerns. Prospective prespecification could not be verified.** | | | | |
| **Overall risk-of-bias judgment: High risk. Driven by Domain 4; Domains 1 and 5 raise some concerns.** | | | | |

**Guo et al. (2015)**

**Outcome: Time to first flatus**

*Assessment target: the time-to-first-flatus result for the TEAS versus sham-stimulation comparison. The report randomized 102 participants (34 per group) to control, TEAS, and sham groups, but Table 1 presents baseline data for only 30, 31, and 26 participants (87 total). The flatus outcome is presented graphically for all three groups without extractable numerical summaries.*

| **Domain / SQ** | **Response** | **Supporting quotation / source evidence** | **Location** | **Rationale** |
| --- | --- | --- | --- | --- |
| 1.1 | **NI** | The report states that 102 patients were randomly divided into three groups of 34; the sequence-generation method is not described. | Methods, 1.1 | Randomization is asserted but the method cannot be verified. |
| 1.2 | **NI** | No allocation-concealment procedure is described. | Methods, 1.1 | Concealment before assignment could not be verified. |
| 1.3 | **PN** | Table 1 reports no statistically significant baseline differences among the participants shown, although the table includes only 87 of the 102 randomized participants. | Results, 2.1; Table 1 | No clear baseline imbalance is evident, but the post-randomization loss limits interpretation. |
| **D1 judgment: Some concerns. Sequence generation and allocation concealment were insufficiently reported.** | | | | |
| 2.1 | **NI** | The TEAS group received active stimulation at ST36 and SP6. The sham group received the same electrical waveform (sparse-dense 2/15 Hz) at non-acupoints 2 cm from ST36 and SP6; the report does not state whether participant blinding was assessed. | Methods, 1.1 | An active non-acupoint sham could support participant masking, but successful masking was not verified. |
| 2.2 | **Y** | Providers had to place electrodes at acupoints versus specified non-acupoints. | Methods, 1.1 | Intervention providers necessarily knew the treatment location. |
| 2.3 | **PN** | No assignment-related deviations or imbalanced co-interventions are described for the TEAS and sham groups. | Methods | No specific deviation is reported. |
| 2.6 | **NI** | Although 34 participants per group were randomized, Table 1 reports 30 control, 31 TEAS, and 26 sham participants, and the report provides no participant flow or explanation for the 15 missing participants. The first-flatus figure does not state the analysis denominator. | Methods, 1.1; Table 1; Results, 2.3/Fig. 3 | It cannot be verified that the flatus result retained participants according to randomized assignment. |
| **D2 judgment: Some concerns. The analysis population cannot be reconciled with the randomized population.** | | | | |
| 3.1 | **N** | The report randomized 102 participants but Table 1 includes only 87; 15 participants (14.7%) are not accounted for, and the first-flatus figure gives no denominator. | Methods, 1.1; Table 1; Fig. 3 | A substantial fraction of the randomized cohort is unexplained. |
| 3.2 | **NI** | No reasons for missingness, sensitivity analysis, or outcome information for the 15 unaccounted participants are reported. | Whole report | Bias from missing outcome data could not be ruled out. |
| 3.3 | **NI** | The reasons for the 15 unaccounted participants are not reported. | Whole report | The relationship between missingness and recovery time is unknown. |
| 3.4 | **NI** | No information is available on reasons or outcome values for the missing participants. | Whole report | Dependence on the true outcome cannot be determined. |
| **D3 judgment: Some concerns. Fifteen of 102 randomized participants are unexplained and the outcome-analysis denominator is not reported.** | | | | |
| 4.1 | **N** | Postoperative anal exhaust time is reported as a gastrointestinal recovery outcome in the abstract and in Results §2.3/Figure 3. The body Methods §1.4 describes blood sampling and IgG/IgM assays but does not specify how anal exhaust time was ascertained. | Abstract, Methods; Results, 2.3; Figure 3 | First flatus/anal exhaust time is an appropriate postoperative recovery milestone, although ascertainment details are not described in the body Methods. |
| 4.2 | **PN** | The ascertainment procedure for postoperative anal exhaust time is not described for either group. | Whole report | Differential ascertainment between groups is not reported, but cannot be excluded because the ascertainment procedure itself is unspecified. |
| 4.3 | **NI** | The sham group received active electrical stimulation at non-acupoints, which could preserve participant masking, but assessor masking and blinding success are not reported. | Methods, 1.1 | Knowledge of assignment at the point of outcome ascertainment cannot be determined. |
| 4.4 | **Y** | First flatus/anal exhaust is a participant-dependent, report-based recovery milestone. | Results, 2.3; Figure 3 | If assignment were known, expectations could influence recognition or reporting of the event. |
| 4.5 | **NI** | The active sham makes participant masking plausible, but its success was not evaluated and assessor masking was not reported. | Methods | There is insufficient information to conclude that treatment knowledge likely influenced measurement. |
| **D4 judgment: Some concerns. The outcome is susceptible to knowledge of assignment, but the active non-acupoint sham may have provided some masking and blinding success was not assessed.** | | | | |
| 5.1 | **NI** | No prospective registration, protocol, or statistical analysis plan is cited. | Whole report | Prospective prespecification could not be verified. |
| 5.2 | **PN** | Anal exhaust time is presented as a single postoperative outcome in the abstract and in Results §2.3/Figure 3, but it is not listed in the body Methods §1.4, which describes only blood sampling and IgG/IgM measurement. | Abstract; Methods, 1.4; Results, 2.3/Figure 3 | Prospective specification of this outcome in the body Methods cannot be verified, but no multiple eligible flatus measurements are reported and there is no direct evidence of selection among multiple measurements. |
| 5.3 | **PN** | No alternative eligible analyses of the flatus outcome are described. | Methods and Results | No evidence of selective analysis was identified. |
| **D5 judgment: Some concerns. Prospective prespecification could not be verified.** | | | | |
| **Overall risk-of-bias judgment: Some concerns. No domain reached high risk, but concerns remain in all five domains.** | | | | |

**He et al. (2020)**

**Outcomes: Time to first flatus and time to first defecation (comparison 1)**

*Assessment target: low-frequency electrical acupoint stimulation (LEAS) added to conventional perioperative care versus conventional perioperative care alone. The trial allocated 184 participants (46 per group) using 184 Excel-generated random numbers. Four participants dropped out and three were excluded after randomization, leaving 177 for analysis; final group sizes were 43 conventional, 45 LEAS, 46 FTS, and 43 FTS+LEAS.*

| **Domain / SQ** | **Response** | **Supporting quotation / source evidence** | **Location** | **Rationale** |
| --- | --- | --- | --- | --- |
| 1.1 | **Y** | The sample size was set at 46 per group (184 total), 184 random numbers were generated in Excel, and participants were randomly allocated to four groups. | Methods, 1.1 | A random-number procedure for sequence generation is described. |
| 1.2 | **NI** | No allocation-concealment mechanism is described. | Methods, 1.1 | Concealment before assignment could not be verified. |
| 1.3 | **PN** | Among the 177 analyzed participants, sex, age, surgical approach, and pathological stage were reported as comparable across groups. | Results, 2.1; Tables 1–2 | No important baseline imbalance suggesting failed randomization was identified, although the baseline table reflects the post-randomization analysis set. |
| **D1 judgment: Some concerns. Allocation concealment was not reported.** | | | | |
| 2.1 | **Y** | The LEAS group received postoperative electrical acupoint stimulation whereas the conventional group did not; no sham stimulation was used. | Methods, 1.7 | Participants could distinguish assignment. |
| 2.2 | **Y** | LEAS was actively delivered in the intervention group and no sham procedure is described. | Methods, 1.7 | Intervention providers knew the assignment. |
| 2.3 | **NI** | Seven participants were removed after randomization. Prespecified dropout criteria included participant-requested withdrawal, postoperative complications, intolerance of low-frequency stimulation, and electrode-patch allergy; exclusion criteria also included ineligibility, incomplete outcome data, and poor compliance. The report does not map the seven removals to specific reasons. | Methods, 1.5–1.6; Results, 2.1 | Some removal criteria could be treatment-related, but the actual reasons for the observed removals are not reported. |
| 2.6 | **N** | Of 46 participants initially allocated to each group, 43 conventional and 45 LEAS participants were analyzed; no intention-to-treat analysis or method retaining the excluded participants is reported. | Methods, 1.1; Results, 2.1 | The analysis excluded post-randomization participants from the effect-of-assignment comparison. |
| 2.7 | **PN** | Losses were 3/46 in the conventional group and 1/46 in the LEAS group. The absolute number was small, but the individual reasons and outcome values are unknown. | Results, 2.1 | A material effect is not demonstrated, but cannot be excluded with confidence. |
| **D2 judgment: Some concerns. Post-randomization exclusions occurred and were not handled by an assignment-based analysis; reasons were incompletely reported.** | | | | |
| 3.1 | **PN** | Outcome data were reported for 43/46 conventional and 45/46 LEAS participants. | Results, 2.1 | A small proportion of randomized participants was missing, with unequal loss between the two groups. |
| 3.2 | **NI** | No sensitivity analysis, imputation, or outcome information for excluded participants is provided. | Whole report | Bias from missing outcome data could not be ruled out. |
| 3.3 | **PY** | Dropout/exclusion criteria included postoperative complications, incomplete outcome data, poor compliance, and potential treatment intolerance; the actual reasons were not linked to individual participants. | Methods, 1.5–1.6 | Several possible reasons for missingness could plausibly relate to postoperative recovery or treatment experience. |
| 3.4 | **NI** | The seven removals were not described by reason or outcome. | Results, 2.1 | There is insufficient information to determine whether missingness depended on the true recovery time. |
| **D3 judgment: Some concerns. A small but incompletely explained proportion of post-randomization outcome data was missing.** | | | | |
| 4.1 | **N** | Time from surgery to first flatus and first defecation was used as the gastrointestinal recovery measure. | Methods, 1.8 | The measures are standard recovery milestones. |
| 4.2 | **N** | The same self-recording procedure was specified for all groups. | Methods, 1.8 | The measurement method itself was intended to be the same across groups. |
| 4.3 | **Y** | The report states that the patients themselves recorded the times (由患者自己记录，研究者统一收集并统计); no sham procedure was used. | Methods, 1.8 | Participants were the primary outcome recorders and were aware of treatment assignment. |
| 4.4 | **Y** | First flatus and defecation are subjective, report-dependent recovery milestones. | Methods, 1.8 | Treatment expectations could influence recognition or timing of reports. |
| 4.5 | **PY** | Self-recording occurred in an open-label comparison without any masking procedure. | Methods, 1.7–1.8 | The combination of self-report and open-label treatment makes influence on measurement plausible. |
| **D4 judgment: High risk. Patient self-recording of report-dependent recovery milestones in an unblinded comparison.** | | | | |
| 5.1 | **NI** | No prospective trial registration, protocol, or statistical analysis plan is cited in the report. | Whole report | Prospective prespecification could not be verified. |
| 5.2 | **PN** | First flatus and first defecation were explicitly specified as gastrointestinal recovery outcomes and both were reported. | Methods, 1.8; Results, 2.2 | No evidence of selective choice among multiple eligible measurements was identified. |
| 5.3 | **PN** | No alternative eligible analyses of these outcomes are described. | Methods and Results | No evidence of selective analysis was identified. |
| **D5 judgment: Some concerns. Prospective prespecification could not be verified.** | | | | |
| **Overall risk-of-bias judgment: High risk. Driven by Domain 4; Domains 1–3 and 5 also raise concerns.** | | | | |

**He et al. (2020)**

**Outcomes: Time to first flatus and time to first defecation (comparison 2)**

*Assessment target: fast-track surgery plus low-frequency electrical acupoint stimulation (FTS+LEAS) versus fast-track surgery (FTS) alone. The same 184-participant randomization and 177-participant analysis set apply.*

| **Domain / SQ** | **Response** | **Supporting quotation / source evidence** | **Location** | **Rationale** |
| --- | --- | --- | --- | --- |
| 1.1 | **Y** | The sample size was set at 46 per group (184 total), 184 random numbers were generated in Excel, and participants were randomly allocated to four groups. | Methods, 1.1 | A random-number procedure for sequence generation is described. |
| 1.2 | **NI** | No allocation-concealment mechanism is described. | Methods, 1.1 | Concealment before assignment could not be verified. |
| 1.3 | **PN** | Among the 177 analyzed participants, sex, age, surgical approach, and pathological stage were reported as comparable across groups. | Results, 2.1; Tables 1–2 | No important baseline imbalance suggesting failed randomization was identified, although the baseline table reflects the post-randomization analysis set. |
| **D1 judgment: Some concerns. Allocation concealment was not reported.** | | | | |
| 2.1 | **Y** | Both groups received the FTS pathway, but only the FTS+LEAS group received electrical acupoint stimulation; no sham stimulation was used. | Methods, 1.7 | Participants could distinguish whether stimulation was added. |
| 2.2 | **Y** | Electrical stimulation was actively delivered only in the FTS+LEAS group. | Methods, 1.7 | Intervention providers knew the assignment. |
| 2.3 | **NI** | Seven participants were removed after randomization. Prespecified dropout criteria included participant-requested withdrawal, postoperative complications, intolerance of low-frequency stimulation, and electrode-patch allergy; exclusion criteria also included ineligibility, incomplete outcome data, and poor compliance. The report does not map the seven removals to specific reasons. | Methods, 1.5–1.6; Results, 2.1 | Some removal criteria could be specific to electrical stimulation, but the actual reasons are not reported. |
| 2.6 | **N** | Of 46 participants initially allocated to each group, all 46 FTS participants but only 43 FTS+LEAS participants were analyzed; no intention-to-treat analysis is reported. | Methods, 1.1; Results, 2.1 | The FTS+LEAS versus FTS comparison contains differential post-randomization exclusion (3 vs 0 participants). |
| 2.7 | **PN** | Losses were 3/46 in FTS+LEAS and 0/46 in FTS. Because intolerance of low-frequency stimulation and electrode-patch allergy were prespecified dropout reasons possible only in stimulation groups, an intervention-related explanation for some losses cannot be excluded. | Methods, 1.5; Results, 2.1 | The differential loss is concentrated in the stimulation group, but only three participants were omitted; a substantial impact on the effect estimate is possible but not judged likely on the available information. |
| **D2 judgment: Some concerns. Differential post-randomization exclusion occurred in the stimulation comparison and reasons were incompletely reported.** | | | | |
| 3.1 | **PN** | Outcome data were reported for 46/46 FTS and 43/46 FTS+LEAS participants. | Results, 2.1 | All missing data in this comparison occurred in the stimulation arm. |
| 3.2 | **NI** | No sensitivity analysis, imputation, or outcome information for the three excluded FTS+LEAS participants is provided. | Whole report | Bias from missing outcome data could not be ruled out. |
| 3.3 | **PY** | Potential reasons include postoperative complications, incomplete outcome data, poor compliance, treatment intolerance, and electrode-patch allergy. | Methods, 1.5–1.6 | Several possible reasons could plausibly relate to treatment experience or postoperative recovery. |
| 3.4 | **NI** | The reasons and outcome values for the three excluded FTS+LEAS participants are not provided. | Results, 2.1 | The dependence of missingness on true recovery time cannot be determined. |
| **D3 judgment: Some concerns. Differential and incompletely explained missing outcome data were present.** | | | | |
| 4.1 | **N** | Time from surgery to first flatus and first defecation was used as the gastrointestinal recovery measure. | Methods, 1.8 | The measures are standard recovery milestones. |
| 4.2 | **N** | The same self-recording procedure was specified for all groups. | Methods, 1.8 | The measurement method itself was intended to be the same across groups. |
| 4.3 | **Y** | Patients themselves recorded the recovery times (由患者自己记录，研究者统一收集并统计), and only the FTS+LEAS group received stimulation. | Methods, 1.7–1.8 | Participants were the primary outcome recorders and were aware of whether stimulation was added. |
| 4.4 | **Y** | First flatus and defecation are subjective, report-dependent recovery milestones. | Methods, 1.8 | Treatment expectations could influence recognition or timing of reports. |
| 4.5 | **PY** | Self-recording occurred in an open-label comparison without any masking procedure. | Methods, 1.7–1.8 | The combination of self-report and open-label treatment makes influence on measurement plausible. |
| **D4 judgment: High risk. Patient self-recording of report-dependent recovery milestones in an unblinded comparison.** | | | | |
| 5.1 | **NI** | No prospective trial registration, protocol, or statistical analysis plan is cited in the report. | Whole report | Prospective prespecification could not be verified. |
| 5.2 | **PN** | First flatus and first defecation were explicitly specified as gastrointestinal recovery outcomes and both were reported. | Methods, 1.8; Results, 2.2 | No evidence of selective choice among multiple eligible measurements was identified. |
| 5.3 | **PN** | No alternative eligible analyses of these outcomes are described. | Methods and Results | No evidence of selective analysis was identified. |
| **D5 judgment: Some concerns. Prospective prespecification could not be verified.** | | | | |
| **Overall risk-of-bias judgment: High risk. Driven by Domain 4; Domains 1–3 and 5 also raise concerns.** | | | | |
