# Supplementary material for: Electrical acupoint stimulation for gastrointestinal recovery after gastrectomy for gastric cancer: a systematic review and meta-analysis
Source: Front Surg. 2026 Sep 16;13:1915755. doi: 10.3389/fsurg.2026.1915755 (PMC13625762; doi:10.3389/fsurg.2026.1915755)
Supplement: Supplementary file 6 [file Table5.docx]

**Supplementary Table S5. Summary statistics reported by Cai et al. (22) and the values derived for the meta-analysis.**

| **Outcome** | **Group** | **n** | **Reported median (IQR)** | **Position of median within IQR^b^** | **Derived mean ± SD^a^** | **Fitted-normal probability below zero, %^c^** |
| --- | --- | --- | --- | --- | --- | --- |
| Time to first flatus, h | Electroacupuncture | 50 | 42.7 (31.7 to 48.2) | 0.67 | 40.87 ± 12.59 | 0.058 |
|  | Sham electroacupuncture | 48 | 46.8 (43.7 to 64.6) | 0.15 | 51.70 ± 15.97 | 0.060 |
| Length of hospital stay, days | Electroacupuncture | 50 | 5 (4 to 6) | 0.50 | 5.00 ± 1.53 | 0.054 |
|  | Sham electroacupuncture | 48 | 5 (5 to 6) | 0.00 | 5.33 ± 0.76 | <0.001 |

Cai et al. (22) stated that non-normally distributed continuous data were summarized as medians with interquartile ranges and compared using Wilcoxon rank-sum tests; the two outcomes shown here were reported in that format. For the outcomes they contributed to the quantitative synthesis, all other trials reported means and standard deviations directly.

^a^ Means and standard deviations were estimated under Scenario C3 of Wan et al. (28), in which the sample size, first quartile, median, and third quartile are available. The mean was estimated as (Q1 + median + Q3)/3 and the standard deviation as (Q3 − Q1)/η(n), where η(n) = 2Φ⁻¹((0.75n − 0.125)/(n + 0.25)) and Φ⁻¹ denotes the standard normal quantile function.

^b^ The position of the median within the interquartile range is (median − Q1)/(Q3 − Q1); a value of 0.50 indicates equal distances from the first and third quartiles. The opposite patterns of within-IQR asymmetry in the two arms mean that cancellation of conversion error between the arms cannot be assumed, so the derived between-group mean difference should be regarded as approximate. Length of hospital stay was reported in whole days, and in the sham arm the median and the first quartile coincide.

^c^ The percentage of a normal distribution with the derived mean and standard deviation that would fall below zero, a value impossible for these outcomes. This diagnostic was calculated only to identify gross incompatibility between the derived summary statistics and a normal approximation. It does not establish that the underlying distributions were normal, does not exclude right-skewness, and, because it is applied to the derived rather than the reported values, does not independently verify the conversion.
