# Supplementary material for: Electrical acupoint stimulation for gastrointestinal recovery after gastrectomy for gastric cancer: a systematic review and meta-analysis
Source: Front Surg. 2026 Sep 16;13:1915755. doi: 10.3389/fsurg.2026.1915755 (PMC13625762; doi:10.3389/fsurg.2026.1915755)
Supplement: Supplementary file 7 [file Table6.docx]

**Supplementary Table S6. Study-family characteristics and relationships among the included reports and related publications.**

**Panel A. Reports contributing to the synthesis**

*The first 13 reports contributed to the quantitative synthesis; the last two contributed to the narrative synthesis only.*

| **Report** | **Trial registration** | **Ethics approval** | **Recruiting center** | **Recruitment period** | **Investigators shared with other reports** | **Randomized (I/C)** | **Analyzed (I/C)^a^** | **Intervention schedule; outcomes contributed** | **Study-family assessment** |
| --- | --- | --- | --- | --- | --- | --- | --- | --- | --- |
| Zheng et al. (35) | Not reported | Not reported^b^ | Tumor Hospital Affiliated to Harbin Medical University, Harbin | Not reported^b^ | None identified | 20 / 20 | 20 / 20 | Continuous from 30 min before induction to the end of surgery, then 30 min three times at three-hourly intervals on the day of surgery and once daily on POD1–2 (5 postoperative sessions); flatus | Independent |
| Chen et al. (36) | Not reported | 2013-051-C01 | Second Affiliated Hospital, Zhejiang University School of Medicine, Hangzhou | Apr 2014 – Feb 2017 | None identified | 33 / 30 | 33 / 30 | 60 min twice daily from POD1 until first flatus or POD5; flatus, LOS | Independent |
| Gu et al. 2018 (19) | Not reported | RMYY-YWLL-2016-01 | Tangshan People’s Hospital, North China University of Science and Technology | Jan 2016 – Aug 2017 | S. Gu, J. Gan, H. Lang (with Gu et al. 2019) | 60 / 60^c^ | 60 / 60 | Continuous from 1 h before induction to 30 min after surgery, then 30 min every 8 h within 2 d; flatus, defecation | Related to the Gu et al. 2019 report by center and investigators; assessed as an independent trial^d^ |
| Gu et al. 2019 (37) | Not reported | RMYY-YWLL-2017-0120 | Tangshan People’s Hospital, North China University of Science and Technology | Feb 2017 – Jun 2018 | S. Gu, J. Gan, H. Lang (with Gu et al. 2018) | 60 / 60 | 58 / 59 | Continuous from 30 min before induction to 30 min after surgery, then 30 min at 8 a.m., 1 p.m. and 6 p.m. within 2 d (7 sessions); flatus, defecation | Related to the Gu et al. 2018 report by center and investigators; assessed as an independent trial^d^ |
| Zhou et al. (38) | ChiCTR1900022692 | QYFYKYLL201812 | Affiliated Hospital of Qingdao University | 1 May – 31 Oct 2019^e^ | None identified | 41 / 41 | 41 / 40 | 30 min twice daily on POD1–3 (6 sessions); flatus, defecation, LOS | Report of record; one further report of the same trial (Panel B) |
| Pei et al. (20) | ChiCTR2100050660 | 2021-NL-053-02 (registration record; approved 3 Jun 2021)^f^ | Seven hospitals^g^, including the Affiliated Hospital of Nanjing University of Chinese Medicine, Nanjing | 27 Oct 2021 – 21 Dec 2023 (registered window 1 Sep 2021 – 30 Sep 2023)^h^ | G. Wang (with Guan et al. and Cai et al.); J. Sun (with Guan et al.)^i^ | 585 total; arm-specific randomized denominators not reconstructable^j^ | 194 / 189 | 30 min at 2, 24, 48, and 72 h after surgery (4 sessions); flatus, defecation, liquid intake, LOS | Index report of the study family; one further report of the same trial (Panel B) |
| Guan et al. (40) | Not reported | 2021-NL-053-03^f^ | Jiangsu Province Hospital of Chinese Medicine, Nanjing | Feb 2021 – Oct 2022^h^ | G. Wang (with Pei et al. and Cai et al.); J. Sun (with Pei et al.); J. Guan, M. Ge, Y. Cai, Z. Jiang (with Cai et al.)^i^ | 22 / 22 | 19 / 18 | 30 min once daily from 1 h after surgery until first flatus; flatus, defecation | Related; independent sample per investigator correspondence |
| Jung et al. (21) | Not reported | DCMC-CR-14-002 | Daegu Catholic University Medical Center, Daegu, Republic of Korea | Mar – Dec 2015 | None identified | 20 / 20 | 18 / 18 | 25–30 min once daily on POD1–5 (5 sessions); flatus, defecation, liquid intake, LOS | Independent |
| Cai et al. (22) | ChiCTR2100050660^k^ | 2021-NL-053-03^f^ | Affiliated Hospital of Nanjing University of Chinese Medicine | Sep 2022 – Jun 2024^h^ | G. Wang (with Pei et al. and Guan et al.); J. Guan, M. Ge, Y. Cai, Z. Jiang (with Guan et al.)^i^ | 51 / 50 | 50 / 48 | 30 min at 2 h after surgery and on the mornings of POD1 and POD2 (3 sessions); flatus, LOS | Related; independent sample per investigator correspondence; the relationship of the reported registration and ethics approval to this trial could not be established from the published reports. Report of record for this trial; one further report (Panel B) |
| Ding and Huang (23) | Not reported | Approved; number not reported | Cancer Hospital, Chinese Academy of Medical Sciences, Beijing | Sep 2018 – Jun 2019^r^ | None identified | 30 / 30 | 30 / 30 | 20 min on the day before surgery and once daily on POD1–5 (6 sessions); flatus, defecation, LOS | Independent^s^ |
| Wang et al. (41) | Not reported | 2022-042-2 (granted before recruitment began) | Shanghai Hospital of Integrated Traditional Chinese and Western Medicine | Jan 2023 – Dec 2024 | None identified^l^ | 45 / 45 | 45 / 45 | 30 min twice daily for 1 week, from POD1; flatus, defecation | Independent |
| Huang and Li (24) | Not reported | Approved; number not reported | Longhua Hospital, Shanghai University of Traditional Chinese Medicine | Mar 2021 – Mar 2022 | None identified | 26 / 26 | 26 / 26 | 20 min twice daily on postoperative days 1–3; flatus, length of stay | Independent |
| Shi et al. (39) | Not reported | Not reported | Lishui Central Hospital, Zhejiang | Jan 2021 – Dec 2022 | Two investigators shared with the excluded report by Ye et al. (2024)^q^ | 90 / 90 | 90 / 90 | 30 min every 12 h from 1 day before surgery to postoperative day 3; flatus, defecation | Related by center and investigators; assessed as independent^q^ |
| Guo et al. (42) | Not reported | Approved; number not reported | Xijing Digestive Disease Hospital, Xi’an | Jul–Nov 2014 | None identified | 34 / 34^n^ | Not reported^n^ | 30 min before induction of anesthesia and on postoperative days 1 and 2 at ST36 and SP6, versus sham stimulation at non-acupoints; flatus (narrative synthesis only) | Independent |
| He et al. (26) | Not reported | Not reported | Shanghai Changhai Hospital, Shanghai (General Surgery and Rehabilitation Medicine wards) | Feb 2016 – Oct 2017 | None identified | 184 in total (46 per group)^o^ | 177 in total (43 / 45 / 46 / 43)^o^ | Low-frequency electrical acupoint stimulation from postoperative day 1 until first flatus, in a four-arm design; flatus and defecation (narrative synthesis only) | Independent |

**Panel B. Related or potentially related records not contributing to the synthesis**

*Panel B lists additional records related to reports in Panel A. The first three are linked reports of included studies; they are counted as reports but not as separate studies, and they are what separates the number of reports from the number of unique underlying studies in the PRISMA flow diagram. The remaining records were excluded at title-and-abstract or full-text screening and are shown for transparency because of study-family, investigator or recruiting-center relationships.*

| **Report** | **Relates to** | **Registration / ethics approval** | **Center** | **Recruitment period** | **Randomized** | **Status in this review** | **Basis** |
| --- | --- | --- | --- | --- | --- | --- | --- |
| Cai Y, Nan H, Xu P, et al. J Clin Acupunct Moxibustion 2025;41(10):38–44 (Chinese) | Cai et al. report | Not stated | Affiliated Hospital of Nanjing University of Chinese Medicine | Sep 2022 – Dec 2023 | 40 / 40 (80) | Linked report of an included study; counted as a report, not as a separate study; no data used | Same center, same stimulation schedule, same sham comparator, same monitoring equipment, eight shared authors. Smaller sample and earlier recruitment end than the Cai et al. report; the participants appear to form part of that sample. The Cai et al. report was retained as the more complete report |
| Guo J, et al. Gastroenterology 2025 (conference abstract)^p^ | Pei et al. report | ChiCTR2100050660 | As the Pei et al. report | As the Pei et al. report | As the Pei et al. report | Linked report of an included study; counted as a report, not as a separate study; no data used | Conference abstract of the trial reported in full by Pei et al. |
| Zhou Y. Clin Nutr ESPEN 2023 (abstract supplement) | Zhou et al. report | Not stated | Not available^m^ | Not available^m^ | Not available^m^ | Linked report of an included study; counted as a report, not as a separate study; no data used | Title identical to the Zhou et al. report; DOI sequence indicates a conference-abstract supplement |
| Li L, Sun J, et al. Chinese Acupuncture & Moxibustion 2026;46(4):537–546 (Chinese) | Pei et al., Guan et al., and Cai et al. reports | Not reported / 2021-NL-053-01 | Jiangsu Province Hospital of Chinese Medicine, one of the seven centers of the Pei et al. report | 27 Oct 2021 – 21 Dec 2023 (identical to the Pei et al. report to the day) | 30 / 30 (60) | Excluded at full-text assessment | Recruitment period identical to the Pei et al. report to the day; same institution; stimulation at 2, 24, 48, and 72 h as in the Pei et al. report; shares J. Sun; ethics approval number shares the protocol stem of the Pei et al., Guan et al., and Cai et al. reports. Independence of the reported data could not be established |
| Wang Y, Gan J, et al. J Liaoning Univ Tradit Chin Med 2024;26(4):158–161 (Chinese) | 2019 and 2018 reports by Gu et al. (same group) | Not reported | Tangshan People’s Hospital, as in the 2019 and 2018 reports by Gu et al. | Not reported | 40 / 40 (80) | Excluded at title/abstract screening | Same center and a shared author, but different acupoints and no postoperative stimulation session. Excluded because the intervention was administered exclusively before or during surgery; no overlap concern arises |
| Ye Z, Shi P, Zhang S, Luo D. New Chin Med 2024;56(8):160–164 (Chinese) | Related to Shi et al. (39) by center and investigators | Lishui Municipal Central Hospital 2023-541 | Lishui Central Hospital, Zhejiang | Jan – Dec 2020 | 40 / 40 (80) | Excluded at full text; independence of the reported data could not be established | Reports outcome values almost identical to those of a separate trial from another center. Two investigators and the recruiting department are shared with Shi et al. (39), but the recruitment periods do not overlap and the age range, disease stage, sample size and outcome values differ, so Shi et al. was assessed as an independent trial |

**Notes**

Abbreviations: C, control group; I, intervention group; LOS, length of hospital stay; POD, postoperative day.

Reports in Panel A are listed in the same order as the studies in Tables 1 and 2, followed by the two trials included in the narrative synthesis only. Values are given as presented in each report; where a report did not provide an item, this is stated as “not reported” rather than left blank. Where a study was described in more than one publication, one report was designated the report of record and all data were taken from it; values were not combined across reports, and the other linked reports were not used.

^a^ Analyzed numbers are those contributing to the primary outcome, time to first flatus. Group sizes for every outcome analysis are shown in Figure 2.

^b^ The Zheng et al. report states neither the recruitment period nor an ethics approval number.

^c^ The Gu et al. 2018 report randomized 180 participants, stratified by age, in equal numbers to a control group, a short-duration stimulation group, and a long-duration stimulation group. Only the long-duration stimulation group and the control group are shown here and contributed to the pooled analyses; stimulation in the short-duration group was administered exclusively during surgery and did not meet the eligibility criteria of this review.

^d^ The 2019 and 2018 reports by Gu et al. were conducted at the same hospital by overlapping investigators, and their recruitment periods overlap between February and August 2017. They were nevertheless assessed as separate trials: the ethics approval numbers differ in both the year and the protocol number (RMYY-YWLL-2016-01 and RMYY-YWLL-2017-0120), the sample sizes and group structures differ (180 in three groups and 120 in two groups), the stimulator models differ, stimulation began 1 h and 30 min before induction respectively, the stimulation intensity ranges differ, and the two reports describe different sham procedures.

^e^ Zhou et al. report May–November 2019 in the abstract and 1 May – 31 October 2019 in the Methods.

^f^ Ethics approval numbers under the protocol stem 2021-NL-053. Version 1.3 of the registration record for ChiCTR2100050660 lists 2021-NL-053-02 for the trial reported by Pei et al. (20); the Pei et al. report itself does not state an approval number. Both the Guan et al. and Cai et al. reports state 2021-NL-053-03. The approving body is named differently in the two reports, as the Ethics Committee of Jiangsu Province Hospital of Chinese Medicine and as the Medical Ethics Committee of the Affiliated Hospital of Nanjing University of Chinese Medicine, but these are two names for one institution: the Pei et al. report gives the combined affiliation Jiangsu Province Hospital of Chinese Medicine, Affiliated Hospital of Nanjing University of Chinese Medicine. That institution is one of the seven recruiting centers of the trial reported by Pei et al. and is the single center of both the Guan et al. and the Cai et al. reports. The three reports therefore share the same institutional center and the same protocol stem, although the full approval number recorded in the registry for Pei et al. is 2021-NL-053-02 rather than 2021-NL-053-03.

^g^ The registration record lists seven recruiting centers for the Pei et al. report.

^h^ Registered timing for ChiCTR2100050660: recruitment 1 September 2021 to 30 September 2023, and study execution 1 January 2021 to 30 November 2023. The Pei et al. report, the index report of that registration, states that recruitment continued until 21 December 2023, about three months beyond the registered recruitment window and 21 days beyond the registered execution period. The Cai et al. report, which cites the same identifier, reports recruitment through June 2024, approximately seven months beyond the registered execution period, so its reported recruitment period is also inconsistent with the timing recorded in the registry. The Guan et al. report began recruiting in February 2021, before the registered recruitment window opened, but does not report a trial registration. These discrepancies are recorded for transparency and were not used on their own to determine study-family independence. Published information alone was not sufficient to exclude participant overlap. The Guan et al. and Cai et al. reports were retained as separate trials after written investigator confirmation that their randomized samples did not overlap with the randomized sample reported by Pei et al.; the differences in protocol and study characteristics are recorded here as contextual information and not as proof of independence.

^i^ Dr Gang Wang is a co-first author of the Pei et al. report and the corresponding author of the Guan et al. and Cai et al. reports. Dr Jianhua Sun is a co-senior and corresponding author of the Pei et al. report, the applicant and study leader of record for ChiCTR2100050660, and a co-author of the Guan et al. report.

^j^ Pei et al. report that 585 participants were randomized and that five were excluded after randomization. The flow diagram then displays 195, 190 and 195 participants in the electroacupuncture, sham and standard-care groups, and the full analysis set comprised 194, 189 and 195. The original group assignment of the five excluded participants was not reported, so arm-specific randomized denominators could not be reconstructed. The registration record gives a planned size of 195 per group in a 1:1:1 allocation, which is a target rather than an allocation result.

^k^ The identifier is a Chinese Clinical Trial Registry number; the Cai et al. report attributes it to ClinicalTrials.gov and describes the registration as retrospective, although recruitment for that trial began after the registration was created.

^l^ The corresponding author of the Wang et al. report, Sunhu Yang, is a different investigator from Jianhua Sun; that trial has no documented relationship to the other reports listed here.

^m^ The full text of this abstract supplement was not retrieved; linkage was based on the identical title and on the DOI sequence. Affiliation, recruitment period, and sample size were not available from the retrieved record.

The acupoint combinations and treatment schedules of the Pei et al., Guan et al., and Cai et al. reports differ from one another, as do those of the 2019 and 2018 reports by Gu et al. (Supplementary Table S2).

^n^ Guo et al. randomized 102 participants in equal numbers (34 per group) to a control group, a transcutaneous electrical acupoint stimulation group, and a sham-stimulation group; only the stimulation and sham groups were relevant to this review. The baseline table presents 30, 31, and 26 participants (87 in total), without explaining the disposition of the remaining 15 randomized participants.

^o^ He et al. randomized 184 participants in equal numbers (46 per group) to conventional care, low-frequency electrical acupoint stimulation (LEAS), fast-track surgery (FTS), and FTS plus LEAS. Four participants withdrew and three were excluded after randomization, leaving 177 analyzed: 43, 45, 46, and 43 participants respectively. Two comparisons were assessed separately in this review (LEAS versus conventional care, and FTS plus LEAS versus FTS). Gastrointestinal recovery outcomes were presented graphically in days, so the trial contributed to the narrative synthesis only.

^p^ This conference abstract reports the multicenter trial published in full by Pei et al. (20) and is unrelated to Guo et al. (42), which is a separate trial by different investigators published in 2015.

^q^ Shi et al. (39) and the excluded report by Ye et al. (2024) come from the same department of the same hospital and share two investigators and a provincial research grant. Their recruitment periods do not overlap (January 2021 to December 2022 and January to December 2020), and the age ranges (65–85 and 42–82 years), disease stages (I–II and II–III), sample sizes and outcome values all differ. No participant overlap was identified, and Shi et al. was therefore assessed as an independent trial.

^r^ The Ding and Huang report gives inconsistent recruitment dates: the Chinese abstract states September 2018 to September 2020, whereas the Methods state September 2018 to June 2019, and the date range in the English abstract is malformed. The Methods dates are used here.

^s^ The Methods state that these 60 patients with gastric cancer undergoing distal gastrectomy were enrolled within a broader randomized trial project on recovery after surgery for gastric and periampullary tumors. No separate report or overlapping sample from that project was identified.
