# Supplementary material for: Electrical acupoint stimulation for gastrointestinal recovery after gastrectomy for gastric cancer: a systematic review and meta-analysis
Source: Front Surg. 2026 Sep 16;13:1915755. doi: 10.3389/fsurg.2026.1915755 (PMC13625762; doi:10.3389/fsurg.2026.1915755)
Supplement: Supplementary file 8 [file Table7.docx]

**Supplementary Table S7. Perioperative care described in each trial and assignment of enhanced recovery after surgery status.**

| **Study** | **ERAS or fast-track terminology in the report** | **Qualifying preoperative or intraoperative domains** | **Qualifying postoperative-recovery domains** | **Domains, n** | **Classification** |
| --- | --- | --- | --- | --- | --- |
| Zheng et al. (35) | Not mentioned | None | D4 combined local anesthetic and opioid patient-controlled epidural analgesia (bupivacaine with fentanyl) | 1 | Conventional care |
| Chen et al. (36) | Discussion (background only); the authors state that enhanced recovery was not applied after surgery | None | None | 0 | Conventional care |
| Gu et al. 2018 (19) | Introduction and Discussion (background only) | None | D4 multimodal analgesia including flurbiprofen axetil | 1 | Conventional care |
| Gu et al. 2019 (37) | Keywords, Introduction and Discussion (background only) | None | D4 multimodal patient-controlled intravenous analgesia | 1 | Conventional care |
| Zhou et al. (38) | Keywords, Introduction, Methods and Discussion | D1 pre-rehabilitation exercise and multidisciplinary assessment; D2 fasting shortened to 6 h with oral carbohydrate 2 h before surgery and no bowel preparation; D3 maintenance of intraoperative normothermia | D5 early enteral feeding; D6 early mobilization; D7 avoidance of routine drains and early removal of the urinary catheter and drainage tubes | 6 | ERAS-based |
| Pei et al. (20) | Introduction (background only); the perioperative pathway is called standard care | None | D4 multimodal analgesia; D5 early water and feed intake; D6 early ambulation; D7 early removal of the urinary catheter and nasogastric tube; D8 functional discharge criteria | 5 | ERAS-based |
| Guan et al. (40) | Abstract, Introduction, Methods and Discussion | D1 patient education, smoking and alcohol cessation, preoperative assessment and nutritional support | D4 multimodal analgesia; D6 early mobilization | 3 | ERAS-based |
| Jung et al. (21) | Discussion (background only) | None | D6 early mobilization | 1 | Conventional care |
| Cai et al. (22) | Introduction and Methods | D2 fasting for 6 h and fluid restriction for 2 h before surgery | D4 multimodal analgesia; D5 early eating; D6 early activity; D7 avoidance of routine nasogastric and abdominal drainage tubes | 5 | ERAS-based |
| Ding and Huang (23) | Title, keywords, abstract, Introduction and Discussion (background only) | None | None | 0 | Conventional care |
| Wang et al. (41) | Discussion (background only) | None | D6 early ambulation encouraged | 1 | Conventional care |
| Huang and Li (24) | Title, Abstract, Introduction, Methods and Discussion | D1 preoperative counseling and rehabilitation training; D2 250 mL warm sugar water 2 h before surgery; D3 intraoperative normothermia with warmed fluids | D5 early enteral nutrition from the second postoperative day; D6 early mobilization from the first to the third postoperative day | 5 | ERAS-based |
| Shi et al. (39) | Methods | D1 prehabilitation for patients with anemia, malnutrition or sarcopenia; D2 oral liquid nutrition 2 h before surgery; D3 intraoperative normothermia and goal-directed fluid therapy | D4 opioid-sparing postoperative analgesia with non-steroidal anti-inflammatory drugs | 4 | ERAS-based |

Abbreviations: ERAS, enhanced recovery after surgery.

ERAS status was assigned from the perioperative co-interventions described as actually implemented in both randomized groups, and not from the authors’ use of ERAS, fast-track or rapid-recovery terminology. Reported practices were mapped to eight review-defined clinical domains informed by enhanced-recovery recommendations for gastrectomy: D1, preoperative optimization or prehabilitation; D2, abbreviated fasting or carbohydrate loading; D3, intraoperative stress reduction such as maintenance of normothermia or goal-directed or restrictive fluid management; D4, multimodal or opioid-sparing analgesia; D5, early oral or enteral intake; D6, early mobilization; D7, avoidance or early removal of nasogastric, abdominal-drain or urinary-catheter tubes; and D8, protocolized functional discharge criteria. Domains D4 to D8 were treated as postoperative-recovery domains.

A trial was classified as ERAS-based when at least three distinct qualifying domains were described as implemented, including at least one postoperative-recovery domain. This threshold is an operational rule for the present exploratory subgroup analysis and is not an ERAS Society diagnostic definition. No trial had exactly two qualifying domains: the seven trials classified as conventional care had none or one, and the six classified as ERAS-based had three or more, so the classification would be unchanged if a two-domain threshold were applied instead.

Practices that were routine or nonspecific rather than characteristic of an enhanced-recovery pathway were not counted as qualifying domains. These included the laparoscopic approach itself, the choice of general anesthetic agent, depth-of-anesthesia monitoring, lung-protective ventilation, antibiotic prophylaxis, prophylaxis of postoperative nausea and vomiting, prophylaxis of deep vein thrombosis, and patient-controlled analgesia without a described multimodal or opioid-sparing component.

Features delivered in the conventional direction, which were not counted: Zheng et al., intraoperative crystalloid and colloid at 10–15 mL/kg/h, approximately 2,500 mL in total; both trials by Gu et al., preoperative fasting of 6 h for solids but 4 h for clear fluids, and, in the 2019 trial, routine transfer to the intensive care unit with the patient still intubated, extubation there, and discharge from the unit on the following morning; Guan et al., oral enteral nutrition started only after the first passage of flatus; Wang et al., routine parenteral nutrition from the first postoperative day for 1 week and routine retention of drains; Huang and Li, routine fasting for both food and fluids from 6 h before surgery, the separately reported 250 mL of warm sugar water 2 h before surgery being counted as carbohydrate loading; Shi et al., routine preoperative bowel preparation.

The two trials included in the narrative synthesis only are not classified here, because they did not contribute to the pooled analyses in which perioperative care was examined as a subgroup.
