# Supplementary material for: Electrical acupoint stimulation for gastrointestinal recovery after gastrectomy for gastric cancer: a systematic review and meta-analysis
Source: Front Surg. 2026 Sep 16;13:1915755. doi: 10.3389/fsurg.2026.1915755 (PMC13625762; doi:10.3389/fsurg.2026.1915755)
Supplement: Supplementary file 9 [file Table8.docx]

**Supplementary Table S8. Reports excluded at full-text assessment, with reasons.**

| **No.** | **First author or source (year)** | **Journal or register** | **Title** | **Reason for exclusion** |
| --- | --- | --- | --- | --- |
| 1 | Zhao Z (2025) | Zhongguo Zhen Jiu | [Herb-spreading moxibustion as an adjuvant treatment for chemotherapy-induced nausea and vomiting of spleen and stomach deficiency cold in gastric cancer: a randomized controlled trial] | Not an intervention of interest |
| 2 | Jing M (2025) | Zhongguo Zhen Jiu | [Effects of thumbtack-needle embedding therapy of auricular acupuncture on gastrointestinal function and intestinal microflora in patients with gastric cancer after operation] | Not an intervention of interest |
| 3 | Ou Y (2024) | Front Med (Lausanne) | Acupuncture and moxibustion as adjunctive therapy for postoperative gastrointestinal dysfunction in gastric cancer: a systematic review and network meta-analysis | Not an intervention of interest |
| 4 | Yu C (2024) | Support Care Cancer | Influence of acupuncture and moxibustion on gastrointestinal function and adverse events in gastric cancer patients after surgery and chemotherapy: a meta-analysis | Not an intervention of interest |
| 5 | Guo JF (2023) | Zhongguo Zhen Jiu | [Effect of acupuncture on functional delayed gastric emptying after gastric cancer surgery based on enhance recovery after surgery] | Not an intervention of interest |
| 6 | Xing R (2022) | Pain Ther | Effect of Transcutaneous Electrical Acupoint Stimulation Combined with Transversus Abdominis Plane Block on Postoperative Recovery in Elderly Patients Undergoing Laparoscopic Gastric Cancer Surgery: A Randomized Controlled Trial | Intervention administered exclusively before or during surgery |
| 7 | Wang XQ (2022) | Explore (NY) | The feasibility and efficacy of perioperative auricular acupuncture technique via intradermal needle buried for postoperative movement-evoked pain after open radical gastrectomy: A randomized controlled pilot trial | Not an intervention of interest |
| 8 | You X (2018) | J Cancer | Zusanli (ST36) Acupoint Injection with Neostigmine for Paralytic Postoperative Ileus following Radical Gastrectomy for Gastric Cancer: a Randomized Clinical Trial | Not an intervention of interest |
| 9 | Chae HD (2016) | J Altern Complement Med | Effect of Acupuncture on Reducing Duration of Postoperative Ileus After Gastrectomy in Patients with Gastric Cancer: A Pilot Study Using Sitz Marker | Not an intervention of interest |
| 10 | Hsiung WT (2015) | Complement Ther Med | Acupressure improves the postoperative comfort of gastric cancer patients: A randomised controlled trial | Not an intervention of interest |
| 11 | Yin SH (2009) | Zhongguo Zhen Jiu | [Clinical study on acupuncture combined with medication in restoration of gastrointestinal functions for postoperative patients with gastric cancer] | Not an intervention of interest |
| 12 | Yin X (2001) | J Tradit Chin Med | Treatment of 104 cases of chemotherapy-induced leukopenia by injection of drugs into Zusanli | Not an intervention of interest |
| 13 | Wan Q (2000) | J Tradit Chin Med | Auricular-plaster therapy plus acupuncture at zusanli for postoperative recovery of intestinal function | Not an intervention of interest |
| 14 | Sun P (1996) | Zhen Ci Yan Jiu | [A study of subtotal gastrectomy under the acupuncture combined with epidural anesthesia of small dosage] | Not an intervention of interest |
| 15 | Kong C (2018) | Academic journal of second military medical university | Curative effect of auricular point sticking for SOX chemotherapy-induced nausea, vomiting and diarrhea | Not an intervention of interest |
| 16 | Wang LL (2019) | Zhongguo Zhen Jiu | Effect of grain-moxibustion on neutrophil to lymphocyte ratio and quality of life in patients with advanced gastric cancer | Not an intervention of interest |
| 17 | Zhang YJ (2020) | Evidence-based complementary and alternative medicine | Efficacy of Acupuncture and Moxibustion as a Subsequent Treatment after Second-Line Chemotherapy in Advanced Gastric Cancer | Not an intervention of interest |
| 18 | Zhou J (2012) | World journal of acupuncture - moxibustion | Clinical research on acupuncture combined with TDP for stump stomach atony after subtotal gastrectomy | Not an intervention of interest |
| 19 | Zhang CZ (2001) | Conference proceedings of the 7th national integrative medicine academic congress on basic and clinical general surgery | Treating late stage gastric cancer with acupoint chemotherapy | Not an intervention of interest |
| 20 | Zhang Huajian (2020) | Chinese nursing research | Application of five-element music gong-modes combined with acupoint massage in hospice care of patients with advanced gastric cancer | Not an intervention of interest |
| 21 | ClinicalTrials.gov (2024) | ClinicalTrials.gov, NCT06744933 | Auricular Acupressure Integrated With a Smartphone Application for Weight Reduction | Not an intervention of interest |
| 22 | Gan H (2009) | Zhong Wai Yi Liao | Flumethasone, Vitamin B6, 6 5 4 - 2 Injection of Zusanli Point for Treatment of Leukopenia in Patients with Cancer | Not an intervention of interest |
| 23 | ClinicalTrials.gov (2018) | ClinicalTrials.gov, NCT03655600 | Acupressure for Pain and Opioid Use Among Breast Cancer Patients | Not an intervention of interest |
| 24 | Yang LH (2025) | Journal of integrative and complementary medicine | Effectiveness of Acupressure in Managing the Pain–Fatigue–Sleep Disturbance–Depression Symptom Cluster in Patients with Cancer: A Systematic Review and Meta-Analysis of Randomized Controlled Trials | Not an intervention of interest |
| 25 | Cheung DST (2025) | ESMO Open | Improving chemotherapy-induced peripheral neuropathy in cancer patients using a combined qigong and self-administered acupressure intervention: a randomized controlled trial | Not an intervention of interest |
| 26 | Zhang C (2025) | Explore | Auricular acupressure for chemotherapy-related insomnia in cancer patients: A systematic review and meta-analysis | Not an intervention of interest |
| 27 | Zhou Y (2025) | Journal of Pain Research | Auricular Acupuncture for Perioperative Pain Management: A Systematic Review and Meta-Analysis of Randomized Controlled Trials | Not an intervention of interest |
| 28 | ClinicalTrials.gov (2024) | ClinicalTrials.gov, NCT06580197 | Application of Auricular Acupuncture Therapy Based on Real-World Studies After Abdominal Surgery | Not an intervention of interest |
| 29 | Han Z (2024) | Obesity surgery | Auricular Acupressure in Relieving PONV and Promoting Gastrointestinal Function Recovery in Females After Laparoscopic Sleeve Gastrectomy: A Prospective Randomized Controlled Trial | Not an intervention of interest |
| 30 | Hu N (2024) | Complementary Medicine Research | Ear-Marking Relief: A Meta-Analysis on the Efficacy of Auricular Acupressure in Alleviating Anxiety Disorders | Not an intervention of interest |
| 31 | Zhu DX (2024) | Frontiers in Neurology | Effects of buccal acupuncture on postoperative analgesia in elderly patients undergoing laparoscopic radical gastrectomy: a randomized controlled trial | Not an intervention of interest |
| 32 | ClinicalTrials.gov (2023) | ClinicalTrials.gov, NCT05911243 | A Randomized Controlled Trial Testing the Effects of an Acupressure Intervention on Appetite and Weight in Patients With Gastric, Esophageal, and Pancreatic Cancer: A Pilot and Feasibility Study | Not an intervention of interest |
| 33 | Wang F (2022) | Frontiers in Oncology | Acupuncture and acupressure with improved cancer-related depression of retrospective studies | Not an intervention of interest |
| 34 | Chen L (2021) | Evidence-based complementary and alternative medicine | Efficacy of Auricular Acupressure in Prevention and Treatment of Chemotherapy-Induced Nausea and Vomiting in Patients with Cancer: A Systematic Review and Meta-Analysis | Not an intervention of interest |
| 35 | O’Hagan ET (2020) | Systematic Reviews | Zolpidem reduces pain intensity postoperatively: A systematic review and meta-analysis of the effect of hypnotic medicines on post-operative pain intensity | Not an intervention of interest |
| 36 | Kong C (2018) | International Journal of Clinical and Experimental Medicine | Auricular point acupressure improved nausea, vomiting, diarrhea and nutritional status in gastric cancer patients receiving oral s-1 therapy | Not an intervention of interest |
| 37 | Song HJ (2015) | Complementary therapies in medicine | Effect of self-acupressure for symptom management: A systematic review | Not an intervention of interest |
| 38 | Lee MS (2010) | BMC Cancer | Moxibustion for cancer care: A systematic review and meta-analysis | Not an intervention of interest |
| 39 | Song N (2021) | World journal of acupuncture - moxibustion | Clinical observation of cancer-related fatigue treated with ginger-isolated moxibustion in the patients with gastric cancer | Not an intervention of interest |
| 40 | Zhao T (2021) | Heilongjiang Zhong Yi Yao | 中医护理联合经皮穴位电刺激在胃癌中的应用价值 | Not an intervention of interest |
| 41 | Chen Q (2016) | Quanke Huli | 穴位注射配合电针在胃癌根治术后胃瘫综合征中的护理效果观察 | Not an intervention of interest |
| 42 | Chen Y (2010) | Xin Zhong Yi | 通腑泻热合剂灌肠加电针对胃癌根治术后胃肠功能恢复的影响 | Not an intervention of interest |
| 43 | Zhang C (2004) | Jiceng Yixue Luntan | 散结饮加电针足三里对胃癌术后患者胃肠功能影响 | Not an intervention of interest |
| 44 | Jiangsu Provincial Hospital of Traditional Chinese Medicine (Affiliated Hospital of Nanjing University of Chinese Medicine) (2026) | ITMCTR, ITMCTR2026000501 | A Study on the Efficacy of Ear-Based Dual Therapy for Preventing and Treating Postoperative Gastrointestinal Dysfunction Following Early Gastric Cancer Surgery Based on the “Ear-Brain-Vital Organs Correlation” Theory | Not an intervention of interest |
| 45 | Qilu Hospital of Shandong University(Qingdao) (2025) | ChiCTR, ChiCTR2500102789 | Effect of percutaneous auricular vagus nerve stimulation on gastrointestinal function in patients with gastric cancer after surgery | Not an intervention of interest |
| 46 | 900TH Hospital of Joint Logistics Support Force (2023) | ChiCTR, ChiCTR2300069538 | Effect of acupoint massage on pain-fatigue-sleep disorder symptom group in postoperative chemotherapy patients with gastric cancer | Not an intervention of interest |
| 47 | Affiliated Hospital of Guizhou Medical University (2021) | ITMCTR, ITMCTR2100005090 | Clinical study on the application of pressing acupuncture and umbilical therapy under the concept of ERAS in the recovery after laparoscopic radical gastric cancer | Not an intervention of interest |
| 48 | University of Washington (2020) | ClinicalTrials.gov, NCT04505553 | Oral Cryotherapy Plus Acupressure and Acupuncture Versus Oral Cryotherapy for Decreasing Chemotherapy-Induced Peripheral Neuropathy From Oxaliplatin-Based Chemotherapy in Patients With Gastrointestinal Cancer | Not an intervention of interest |
| 49 | Sun M (2026) | J Natl Cancer Inst | RE: Electroacupuncture for quality of life during adjuvant chemotherapy in gastric cancer: a randomized trial | Not an outcome of interest |
| 50 | Gu CY (2004) | Zhongguo Zhong Xi Yi Jie He Za Zhi | [Effects of different anesthesia methods on the T lymphocyte subsets and hemodynamics of patients with abdominal tumor in peri-operational period] | Not an outcome of interest |
| 51 | Liu J (2002) | Zhongguo Zhong Xi Yi Jie He Za Zhi | [Influence of combined therapy of guben yiliu III, moxibustion and chemotherapy on immune function and blood coagulation mechanism in patients with mid-late stage malignant tumor] | Not an outcome of interest |
| 52 | Dang W (1998) | J Tradit Chin Med | Clinical study on acupuncture treatment of stomach carcinoma pain | Not an outcome of interest |
| 53 | ITMCTR (2025) | ITMCTR, ITMCTR2025000920 | Effect of transcutaneous acupoint electrical stimulation combined with esketamine on postoperative anxiety and depression in patients with abdominal malignant tumors: a multicenter prospective randomized double-blind controlled trial | Not an outcome of interest |
| 54 | Oztas B (2019) | Contemporary nurse | The effects of two different electrical stimulation methods on the pain intensity of the patients who had undergone abdominal surgery with a midline incision: randomized controlled clinical trial | Not an outcome of interest |
| 55 | Chang X (2024) | Journal of integrative and complementary medicine | ELECTRO-ACUPUNCTURE’S EFFECT ON CHEMOKINE OF CHEMOTHERAPY-RELATED MYELOSUPPRESSION IN GASTRIC CANCER PATIENTS UNDERGOING ADJUVANT CHEMOTHERAPY | Not an outcome of interest |
| 56 | ChiCTR (2024) | ChiCTR, ChiCTR2400093820 | To explore the molecular mechanism of electroacupuncture in improving postoperative cognitive dysfunction via regulating Th17/Treg immune axis | Not an outcome of interest |
| 57 | Wan SY (2015) | Shanghai Zhen Jiu Za Zhi | Clinical observation of herbal foot-bath plus acupoint application in improving sleep quality prior to stomach cancer surgery | Not an outcome of interest |
| 58 | Kim JE (2015) | Trials | Acupuncture for chronic fatigue syndrome and idiopathic chronic fatigue: A multicenter, nonblinded, randomized controlled trial | Not an outcome of interest |
| 59 | ClinicalTrials.gov (2022) | ClinicalTrials.gov, NCT05601479 | Preventive Effect of Acetyl-L-carnitine on Oxaliplatin-induced Peripheral Neuropathy | Not an outcome of interest |
| 60 | Wang H (2024) | International Wound Journal | Enhancing wound recovery in chemotherapy-induced leukopenia for malignant tumours: A meta-analysis of acupuncture treatment efficacy | Not an outcome of interest |
| 61 | Liu W (2024) | Integrative Cancer Therapies | Systematic Review and Meta-analysis of Acupuncture for Modulation of Immune and Inflammatory Markers in Cancer Patients | Not an outcome of interest |
| 62 | Shih YW (2023) | Journal of integrative and complementary medicine | Effectiveness of Acupuncture for Relieving Chemotherapy-Induced Bone Marrow Suppression: A Systematic Review with a Meta-analysis and Trial Sequential Analysis | Not an outcome of interest |
| 63 | Wang L (2019) | World journal of acupuncture - moxibustion | Abdominal acupuncture combined with megestrol acetate for advanced cancer-related anorexia: A randomized controlled trial | Not an outcome of interest |
| 64 | Sun K (2019) | Advances in Integrative Medicine | Acupuncture treatment for chronic atrophic gastritis: A systematic review and meta-analysis of randomized controlled trials | Not an outcome of interest |
| 65 | Kang HJ (2017) | JAMS Journal of Acupuncture and Meridian Studies | Cancer Pain Control for Advanced Cancer Patients by Using Autonomic Nerve Pharmacopuncture | Not an outcome of interest |
| 66 | Enblom A (2011) | PLoS ONE | Getting the grip on nonspecific treatment effects: Emesis in patients randomized to acupuncture or sham compared to patients receiving standard care | Not an outcome of interest |
| 67 | Feng Y (2011) | Journal of Traditional Chinese Medicine | Clinical research of acupuncture on malignant tumor patients for improving depression and sleep quality | Not an outcome of interest |
| 68 | Yamaoka K (1998) | International Medical Journal | Validity of the Japanese version of the questionnaire for Quality of Life measurement (QOL20) | Not an outcome of interest |
| 69 | Yu ML (2023) | World journal of acupuncture - moxibustion | Bibliometric characteristics of clinical studies on acupuncture for the management of cancer-related symptoms | Not an outcome of interest |
| 70 | Krukowska K (2024) | Frontiers in Oncology | The effectiveness of acupuncture in cancer pain treatment | Not an outcome of interest |
| 71 | Liang Q (2020) | American Journal of Chinese Medicine | Acupuncture for Cancer Pain - An Adjuvant Therapy for Cancer Pain Relief | Not an outcome of interest |
| 72 | Cheng J (2025) | Zhongguo Zhen Jiu | 围术期经皮穴位电刺激对老年患者术后疲劳综合征的影响 | Not an outcome of interest |
| 73 | Fu W (2025) | Zhongguo Yiyao Kexue | 经皮穴位电刺激对胃癌患者术后全身炎症反应综合征发生率的影响 | Not an outcome of interest |
| 74 | Xu H (2023) | Zhongguo Zhen Jiu | 电针促进腹腔镜胃癌根治术患者胃肠功能恢复：随机对照试验 | Not an outcome of interest |
| 75 | Chang X (2022) | Zhongguo Zhen Jiu | 针刺改善胃癌术后辅助化疗期间患者症状负荷疗效观察 | Not an outcome of interest |
| 76 | Shi Y (2020) | Shiyong Zhongliu Za Zhi | 电针联合心理干预对晚期胃癌化疗患者免疫功能及生活质量的影响 | Not an outcome of interest |
| 77 | Zhu YH (2020) | Journal of Acupuncture and Tuina Science | Effects of electroacupuncture plus drug anesthesia on pain and stress response in patients after radical surgery for stomach cancer | Not an outcome of interest |
| 78 | Yueyang Integrated Traditional Chinese and Western Medicine Hospital affiliated with Shanghai University of Traditional Chinese Medicine (2026) | ITMCTR, ITMCTR2026000476 | Electroacupuncture for Gastric Cancer-Related Sarcopenia with Qi-Blood Deficiency: A Clinical Efficacy and Safety Study | Not an outcome of interest |
| 79 | Guangzhou University of Traditional Chinese Medicine (2025) | ClinicalTrials.gov, NCT07098949 | Acupuncture Combined With Chemotherapy for Gastric Cancer After Surgery | Not an outcome of interest |
| 80 | Guangdong Provincial Hospital of Chinese Medicine (2024) | ITMCTR, ITMCTR2024000289 | Clinical application of electroacupuncture assisted Opioid-sparing anesthesia in laparoscopic abdominal surgery | Not an outcome of interest |
| 81 | WeiFang People’s Hospital (2024) | ChiCTR, ChiCTR2400083948 | Effect of percutaneous acupoint electrical stimulation on postoperative anti-inflammatory effects in patients with complete laparoscopic distal gastric cancer: a multicenter prospective randomized controlled trial | Not an outcome of interest |
| 82 | GERCOR - Multidisciplinary Oncology Cooperative Group (2023) | ClinicalTrials.gov, NCT05850130 | Acupuncture for Oxaliplatin-induced Peripheral Neuropathy in Gastro-intestinal Cancer Patients | Not an outcome of interest |
| 83 | The Affiliated Hospital of Xuzhou Medical University (2022) | ITMCTR, ITMCTR2200006585 | Effect of transcutaneous acupoint electrical stimulation assisted general anesthesia on postoperative fatigue in patients undergoing laparoscopic radical gastrectomy for gastric cancer | Not an outcome of interest |
| 84 | Jiangsu Province Hospital of Chinese Medicine (2022) | ChiCTR, ChiCTR2200060159 | The Effect and Mechanism of Predictive Intervention of Auricular Point Sticking and Pressing Based on Sentinel Symptom on Pain-Fatigue-Sleep Disorder Symptoms in Gastric Cancer Patients Undergoing Chemotherapy | Not an outcome of interest |
| 85 | Guangzhou University of Traditional Chinese Medicine (2020) | ClinicalTrials.gov, NCT04360577 | Acupuncture for QoL and Symptoms in Gastric Cancer During Adjuvant Chemotherapy | Not an outcome of interest |
| 86 | Guangzhou University of Traditional Chinese Medicine (2018) | ClinicalTrials.gov, NCT03753399 | Effect of Acupuncture for QoL in Gastric Cancer Patients Undergoing Adjuvant Chemotherapy: a Pilot Study | Not an outcome of interest |
| 87 | Department of Anaesthesiology Harbin Medical University Cancer Hospital (2018) | ChiCTR, ChiCTR1800018978 | Effects of transcutaneous electrical acupoint stimulation on postoperative pain management and immune function in gastrectomy for gastric cancer | Not an outcome of interest |
| 88 | Fourth Military Medical University (2014) | ClinicalTrials.gov, NCT02193477 | Effect of TEAS on the Incidence Rate of SIRS in Patients Undergoing Abdominal Surgery | Not an outcome of interest |
| 89 | Hou YT (2023) | World J Gastrointest Surg | Transcutaneous electrical acupoint stimulation in adult patients receiving gastrectomy/colorectal resection: A randomized controlled trial | Mixed cancer population; gastric cancer-specific data not separable |
| 90 | Qiu G (2021) | Evid Based Complement Alternat Med | Perioperative Electroacupuncture Can Accelerate the Recovery of Gastrointestinal Function in Cancer Patients Undergoing Pancreatectomy or Gastrectomy: A Randomized Controlled Trial | Mixed cancer population; gastric cancer-specific data not separable |
| 91 | Wu JG (1997) | Zhongguo Zhong Xi Yi Jie He Za Zhi | [Effect of acupoint irradiation with Q-wave millimeter microwave on peripheral white blood cells in post-operational treatment with chemotherapy in stomach and colorectal cancer patients] | Mixed cancer population; gastric cancer-specific data not separable |
| 92 | Wang J (2023) | Pain and therapy | Transcutaneous Electrical Acupoint Stimulation Improves Postoperative Sleep Quality in Patients Undergoing Laparoscopic Gastrointestinal Tumor Surgery: A Prospective, Randomized Controlled Trial | Mixed cancer population; gastric cancer-specific data not separable |
| 93 | Hou Y (2023) | Frontiers in psychiatry | Effects of electroacupuncture on perioperative anxiety and stress response in patients undergoing surgery for gastric or colorectal cancer: study protocol for a randomized controlled trial | Mixed cancer population; gastric cancer-specific data not separable |
| 94 | Zhan W (2020) | European journal of integrative medicine | Addition of transcutaneous electric acupoint stimulation to transverse abdominis plane block for postoperative analgesia in abdominal surgery: a randomized controlled trial | Mixed cancer population; gastric cancer-specific data not separable |
| 95 | Guo M (2022) | World journal of clinical cases | Effect of intradermal needle therapy at combined acupoints on patients’ gastrointestinal function following surgery for gastrointestinal tumors | Mixed cancer population; gastric cancer-specific data not separable |
| 96 | Chung SL (2024) | BMC Complementary Medicine and Therapies | Clinical study of electroacupuncture on the recovery of gastrointestinal dysfunction after laparoscopic surgery for gastrointestinal cancer - study protocol for a randomized controlled trial | Mixed cancer population; gastric cancer-specific data not separable |
| 97 | Bai YF (2020) | Trials | Transcutaneous electrical acupuncture stimulation (TEAS) for gastrointestinal dysfunction in adults undergoing abdominal surgery: Study protocol for a prospective randomized controlled trial | Mixed cancer population; gastric cancer-specific data not separable |
| 98 | Zhang B (2018) | American Journal of Gastroenterology | Needleless Transcutaneous Electrical Acustimulation: A Pilot Study Evaluating Improvement in Post-Operative Recovery | Mixed cancer population; gastric cancer-specific data not separable |
| 99 | Yuan Y (2023) | Zhonghua Zhong Yi Yao Za Zhi | 围手术期电针减少胃及胰腺癌患者术后止痛药物使用的随机对照研究 | Mixed cancer population; gastric cancer-specific data not separable |
| 100 | Pan H (2016) | Zhen Ci Yan Jiu | 电针足三里、内关对开腹术后胃肠功能障碍的影响:随机对照试验 | Mixed cancer population; gastric cancer-specific data not separable |
| 101 | Zhou P (2024) | Zhongguo Zhen Jiu | 电针预处理对腹腔镜胃肠道肿瘤手术患者苏醒质量的影响 | Mixed cancer population; gastric cancer-specific data not separable |
| 102 | Li L (2026) | Zhongguo Zhen Jiu | 电针对胃癌术后患者胃肠功能障碍及血清代谢物的影响 | Independence of the reported data could not be established |
| 103 | Han K (2025) | Jinzhou Yike Daxue Xuebao | 经皮穴位电刺激疗法联合PCIA对胃癌根治术后胃肠功能恢复及疼痛的影响 | Independence of the reported data could not be established |
| 104 | Ye Z (2024) | Xin Zhong Yi | 经皮穴位电刺激对胃癌术后疼痛及胃肠蠕动功能的影响 | Independence of the reported data could not be established |
| 105 | Wang L (2022) | Med Acupunct | Electroacupuncture Before Gastrectomy Accelerates Recovery from Gastrointestinal Dysfunction: A Feasibility Study | Intervention administered exclusively before or during surgery |
| 106 | Tang F (2020) | Zhongguo Yixue Chuangxin | 腹腔镜下胃癌根治术患者实施经皮穴位电刺激的效果探讨 | Intervention administered exclusively before or during surgery |
| 107 | Liu Z (2020) | Jiangxi Yiyao | 经皮穴位电刺激对腹腔镜下胃癌根治术患者免疫功能的影响 | Intervention administered exclusively before or during surgery |
| 108 | Chang X (2023) | Health Qual Life Outcomes | Electro-acupuncture for health-related quality of life and symptoms in patients with gastric cancer undergoing adjuvant chemotherapy (EAGER): a protocol for a multicenter randomized controlled trial | Published protocol or trial-registration record only |
| 109 | Wang XQ (2020) | European journal of integrative medicine | Perioperative auricular acupuncture technique via intradermal needling buried for postoperative movement-evoked pain after open radical gastrectomy: Study protocol for a pilot randomized controlled trial | Published protocol or trial-registration record only |
| 110 | Daegu Catholic University Medical Center (2015) | ClinicalTrials.gov, NCT02480361 | Effect of Acupuncture on Postoperative Ileus After Gastric Surgery (EAPIG) | Published protocol or trial-registration record only |
| 111 | Que H (2021) | Xin Zhong Yi | 电针仪穴位刺激联合情志护理对胃肿瘤围手术期患者自我效能、胃肠功能及生活质量的影响 | Intervention modality/protocol could not be adequately characterized |
| 112 | She J (2015) | Shaanxi Zhong Yi | 电针疗法促进全胃切除术后肠功能恢复的临床研究 | Intervention modality/protocol could not be adequately characterized |
| 113 | Suzuki M (2021) | J Clin Med | Pilot study of acupuncture’s antispasmodic effect on upper gastrointestinal tract during endoscopic submucosal dissection for early gastric cancer: Controlled clinical trial | Not an eligible population or procedure |
| 114 | Zhang S (2025) | Xin Zhong Yi | 经皮穴位电刺激联合加速康复外科治疗胃癌术后肠麻痹临床研究 | Treatment of an established postoperative gastrointestinal motility disorder |
| 115 | Hsieh YL (2016) | Acupuncture in Medicine | Laser acupuncture attenuates oxaliplatin-induced peripheral neuropathy in patients with gastrointestinal cancer: A pilot prospective cohort study | Not an eligible study design |

A total of 115 reports were excluded after full-text assessment. Reports that were additional publications of an already included study were not excluded; these are described in Supplementary Table S6. Seven records were identified as ongoing or awaiting results, two at title-and-abstract screening and five at full-text assessment; five were trial-registration records and two were published study protocols, and none reported trial results. These records were documented separately and were not counted as exclusions.

Chinese-language reports are listed with author names and journal titles in Hanyu Pinyin where applicable. Article titles are reproduced as recorded in the screening database: English translations supplied by bibliographic databases are retained, whereas records retrieved in Chinese are shown in the original language. Registry records are listed under the name of the register together with the registration identifier.

Three reports were excluded because the independence of the reported data could not be established from the published information.

Li et al. (2026) was conducted at one of the centers participating in the included multicenter trial by Pei et al., reported the same recruitment period, used a closely matching postoperative electroacupuncture schedule, and shared investigators with that trial. The report did not state whether its 60 participants were a subset of, or independent from, the Pei trial. Unlike the three included reports by Pei et al., Cai et al. and Guan et al., for which written correspondence from the investigators indicated that the randomized samples did not overlap, no such confirmation was available for this report.

Han et al. (2025) and Ye et al. (2024) described nominally separate cohorts recruited at different hospitals and during different periods, and used different comparator interventions. However, the two reports gave identical sex and disease-stage distributions and closely matching age summaries in both trial arms, together with highly similar postoperative recovery values: time to first flatus was 51.76 ± 4.65 versus 51.64 ± 4.32 hours in the control arms and 25.65 ± 2.54 versus 25.48 ± 2.61 hours in the intervention arms, and time to first defecation was 64.65 ± 11.76 versus 64.94 ± 11.08 hours and 42.65 ± 9.76 versus 42.28 ± 9.43 hours respectively. The published reports did not provide information sufficient to establish that the two datasets were independent, and both were therefore excluded.

These exclusions were based solely on the information reported in the publications. They do not imply any conclusion regarding the conduct of the studies concerned.

Two pairs of entries are separate reports of the same excluded study and were not treated as duplicate records: the trial-registration record sponsored by Daegu Catholic University (NCT02480361) corresponds to the pilot study reported by Chae et al. (2016), and the protocol published by Wang et al. (2020) corresponds to the randomized pilot trial reported by the same group in 2022.

Reports excluded because EAS was accompanied by another traditional or acupoint-based treatment prescribed separately from the EAS protocol were recorded under the broader category “Not an intervention of interest.”

Where a report met more than one exclusion criterion, a single representative reason was recorded; no fixed order of precedence among criteria was specified in advance.
