# Supplementary material for: Electrical acupoint stimulation for gastrointestinal recovery after gastrectomy for gastric cancer: a systematic review and meta-analysis
Source: Front Surg. 2026 Sep 16;13:1915755. doi: 10.3389/fsurg.2026.1915755 (PMC13625762; doi:10.3389/fsurg.2026.1915755)
Supplement: Supplementary file 10 [file Table9.docx]

**Supplementary Table S9. Complete leave-one-out analyses.**

**Panel A. Time to first flatus (13 trials)**

| **Trial omitted** | **MD (95% CI)** | **Model statistics** | **95% prediction interval** |
| --- | --- | --- | --- |
| *None (primary analysis)* | -9.61 (-12.81 to -6.42) | *k = 13; τ² = 16.29; I² = 82.6%; P* ***<*** *0.001* | -18.97 to -0.25 |
| Zheng et al. (2007) | -9.74 (-13.36 to -6.13) | *k = 12; τ² = 19.51; I² = 84.4%; P* ***<*** *0.001* | -20.12 to 0.63 |
| Chen et al. (2018) | -9.71 (-13.21 to -6.21) | *k = 12; τ² = 18.06; I² = 84.9%; P* ***<*** *0.001* | -19.69 to 0.28 |
| Gu et al. (2018) | -10.11 (-12.95 to -7.26) | *k = 12; τ² = 8.97; I² = 68.4%; P* ***<*** *0.001* | -17.29 to -2.93 |
| Gu et al. (2019) | -10.07 (-13.61 to -6.53) | *k = 12; τ² = 19.12; I² = 84.6%; P* ***<*** *0.001* | -20.32 to 0.19 |
| Zhou et al. (2021) | -8.99 (-12.17 to -5.81) | *k = 12; τ² = 13.63; I² = 80.9%; P* ***<*** *0.001* | -17.72 to -0.26 |
| Pei et al. (2025) | -9.38 (-12.85 to -5.90) | *k = 12; τ² = 16.97; I² = 83.8%; P* ***<*** *0.001* | -19.09 to 0.33 |
| Guan et al. (2025) | -9.70 (-13.17 to -6.23) | *k = 12; τ² = 17.72; I² = 84.8%; P* ***<*** *0.001* | -19.60 to 0.19 |
| Jung et al. (2017) | -9.36 (-12.73 to -5.99) | *k = 12; τ² = 16.14; I² = 83.5%; P* ***<*** *0.001* | -18.82 to 0.10 |
| Cai et al. (2025) | -9.62 (-13.17 to -6.08) | *k = 12; τ² = 18.43; I² = 84.9%; P* ***<*** *0.001* | -19.71 to 0.47 |
| Ding and Huang (2021) | -8.71 (-11.58 to -5.84) | *k = 12; τ² = 11.37; I² = 78.1%; P* ***<*** *0.001* | -16.67 to -0.76 |
| Wang et al. (2026) | -10.20 (-13.69 to -6.71) | *k = 12; τ² = 18.15; I² = 78.2%; P* ***<*** *0.001* | -20.21 to -0.20 |
| Huang and Li (2023) | -9.31 (-12.40 to -6.21) | *k = 12; τ² = 15.20; I² = 82.8%; P* ***<*** *0.001* | -18.43 to -0.19 |
| Shi et al. (2024) | -10.11 (-13.67 to -6.55) | *k = 12; τ² = 19.26; I² = 82.0%; P* ***<*** *0.001* | -20.40 to 0.18 |

**Panel B. Time to first defecation (9 trials)**

| **Trial omitted** | **MD (95% CI)** | **Model statistics** | **95% prediction interval** |
| --- | --- | --- | --- |
| *None (primary analysis)* | -15.88 (-23.31 to -8.46) | *k = 9; τ² = 78.13; I² = 90.3%; P = 0.001* | -37.58 to 5.81 |
| Gu et al. (2018) | -13.36 (-19.49 to -7.22) | *k = 8; τ² = 35.05; I² = 80.1%; P = 0.001* | -28.64 to 1.93 |
| Gu et al. (2019) | -17.21 (-25.13 to -9.28) | *k = 8; τ² = 74.85; I² = 90.3%; P = 0.001* | -39.14 to 4.73 |
| Zhou et al. (2021) | -14.86 (-23.09 to -6.62) | *k = 8; τ² = 82.05; I² = 90.9%; P = 0.004* | -37.80 to 8.09 |
| Pei et al. (2025) | -16.03 (-24.63 to -7.43) | *k = 8; τ² = 90.21; I² = 92.0%; P = 0.003* | -40.08 to 8.02 |
| Guan et al. (2025) | -16.51 (-24.61 to -8.41) | *k = 8; τ² = 81.63; I² = 91.6%; P = 0.002* | -39.36 to 6.34 |
| Jung et al. (2017) | -16.46 (-24.89 to -8.02) | *k = 8; τ² = 86.62; I² = 91.8%; P = 0.002* | -40.02 to 7.11 |
| Ding and Huang (2021) | -14.70 (-22.61 to -6.79) | *k = 8; τ² = 77.20; I² = 90.9%; P = 0.003* | -36.93 to 7.53 |
| Wang et al. (2026) | -16.79 (-25.25 to -8.34) | *k = 8; τ² = 85.24; I² = 85.9%; P = 0.002* | -40.20 to 6.62 |
| Shi et al. (2024) | -16.83 (-25.24 to -8.42) | *k = 8; τ² = 84.29; I² = 87.6%; P = 0.002* | -40.11 to 6.45 |

**Panel C. Length of hospital stay (7 trials)**

| **Trial omitted** | **MD (95% CI)** | **Model statistics** | **95% prediction interval** |
| --- | --- | --- | --- |
| *None (primary analysis)* | -0.63 (-1.36 to 0.10) | *k = 7; τ² = 0.25; I² = 57.0%; P = 0.079* | -2.07 to 0.80 |
| Chen et al. (2018) | -0.54 (-1.38 to 0.29) | *k = 6; τ² = 0.24; I² = 57.9%; P = 0.155* | -2.06 to 0.97 |
| Zhou et al. (2021) | -0.74 (-1.69 to 0.22) | *k = 6; τ² = 0.42; I² = 64.9%; P = 0.103* | -2.66 to 1.19 |
| Pei et al. (2025) | -0.76 (-1.47 to -0.06) | *k = 6; τ² = 0.16; I² = 46.0%; P = 0.038* | -2.01 to 0.48 |
| Jung et al. (2017) | -0.67 (-1.58 to 0.23) | *k = 6; τ² = 0.36; I² = 67.7%; P = 0.115* | -2.46 to 1.11 |
| Cai et al. (2025) | -0.75 (-1.71 to 0.21) | *k = 6; τ² = 0.43; I² = 61.6%; P = 0.102* | -2.69 to 1.19 |
| Ding and Huang (2021) | -0.49 (-1.10 to 0.12) | *k = 6; τ² = 0.12; I² = 42.1%; P = 0.094* | -1.57 to 0.60 |
| Huang and Li (2023) | -0.50 (-1.35 to 0.35) | *k = 6; τ² = 0.20; I² = 49.4%; P = 0.190* | -1.94 to 0.94 |

Abbreviations: CI, confidence interval; MD, mean difference; NC, not calculated.

P values are for the test of the pooled mean difference and are not the P values from Cochran’s Q test of heterogeneity.

Each row shows the pooled estimate obtained when the named trial was omitted and the remaining trials were reanalyzed using the primary model: restricted maximum likelihood with Hartung–Knapp–Sidik–Jonkman intervals and tests when τ² was greater than zero and more than two trials contributed, and Wald-type inference otherwise. Negative values favor electrical acupoint stimulation. Prediction intervals are shown for analyses to which at least five trials contributed and are otherwise marked NC.

A complete leave-one-out analysis was not applicable to time to first liquid intake, because only two trials contributed and omitting either leaves a single trial.
